# Supplementary material for: Development of an enhanced scoring system to predict ICU readmission or in-hospital death within 24 hours using routine patient data from two NHS Foundation Trusts
Source: BMJ Open. 2024 Apr 12;14(4):e074604. doi: 10.1136/bmjopen-2023-074604 (PMC11029184; doi:10.1136/bmjopen-2023-074604)
Supplement: Supplementary data [file bmjopen-2023-074604supp001.pdf]

Additional file 1 – Model development

We describe in more detail the methods used to develop the model in this section.

Predictors

Our proposed approach uses a conceptual model in which we estimate the risk of a patient experiencing an adverse event immediately after discharge from ICU. This risk score (RS_1) is estimated from variables recorded during their stay in the ICU. After discharge from ICU, the patient's risk of experiencing an adverse event within the next 24 hours is updated in a time-varying manner (RS_2), using those measurements of the vital signs that are routinely measured during subsequent ward care.

ICU-based feature representation:

To estimate the risk of a patient experiencing an adverse event immediately after discharge from ICU, we considered candidate variables that were available electronically in our databases. The final list of candidate variables included the following physiological variables:

- Heart rate (HR), measured in beats per minute
- Respiratory rate (RR), in breaths per minute
- Systolic (SBP), diastolic and mean blood pressure, in mmHg
- Temperature, in degrees Celsius
- Peripheral oxygen saturation (SpO_2), in %
- Level of consciousness, using the Glasgow Coma Scale (GCS)

The following laboratory test results were included in the list:

- Alveolar-arterial oxygen partial pressure gradient ($AaDO_2$), in kPa
- Albumin, in g/L
- Bilirubin (total), in μmol/L
- Calcium (adjusted), in mmol/L
- Creatinine, in mmol/L
- C-reactive protein (CRP), in mg/L
- Haematocrit (HCT), in %
- Haemoglobin (HGB), in g/dL
- Lactate, in mmol/L
- Mean corpuscular volume (MCV), in fL
- Ratio of partial pressure of oxygen : fraction of inspired oxygen (PaO_2/FiO_2), in kPa
- pH
- Platelet count, in 10^9 cells/L

- Potassium, in mmol/L
- Sodium, in mmol/L
- Urea, measured in mmol/L
- Urine output, in mL
- White blood cell count (WBC), in 10^9 cells/L

The list also included demographic and administrative-based variables, such as age at admission, gender, number of hours between hospital admission and ICU admission, and the Index of Multiple Deprivation score (1), derived for patients with a valid postcode. We included variables related to procedures and treatments, such as the number of vasoactive drugs administered, total fluid balance, administration of insulin, enteral and parenteral nutrition feeding, and the use of mechanical ventilation, tracheostomy and central venous catheters.

To determine the risk of future compound outcome after discharge from the ICU, we derived several features from all candidate variables acquired during the patients' stay in the ICU. These features are based on the extremes of the variables considered. We generated maximum, minimum and variability (as given by the standard deviation) features for the physiological variables and laboratory test results from different periods of the ICU stay (including the first 24 hours of the patient in the ICU, their last 72, 48, and 24 hours, and/or their entire ICU stay). Additional dichotomous variables were generated if the values were in the upper or lower 5th percentile of the observed corresponding ranges, in order to account for potential non-linear associations of the variables with the adverse outcomes. Procedure and treatment variables were converted to dichotomous features for indicating whether a given procedure was performed or not, or whether a given medication was administered or not, over the entire ICU stay or in the last 24 hours of the ICU stay. This procedure resulted in 161 candidate features (including features from demographic information) for building a prediction model.

Post-ICU feature representation:

All vital-sign observations performed after discharge from ICU, as part of routine patient monitoring on acute care wards and collected prospectively for this study, were considered for analysis. Each set of vital signs include heart rate, systolic blood pressure, respiratory rate, body temperature, neurological status assessment using the Alert-Verbal-Painful-Unresponsive (AVPU) scale, peripheral oxygen saturation from pulse oximetry (SpO_2), a record of whether the patient was receiving supplemental oxygen at the time of the SpO_2 measurement, and the date and time of the observation. Vital-sign measurements are typically recorded every 4 or 6 hours throughout the patient's stay on the ward. At each measurement timestamp, in case of an incomplete vital-sign observation set, we used the most recent value of each variable (i.e., by carrying forward the last measurement).

Pre-processing and missing data

We identified obvious deviations from expected distributions and ranges of the data features using frequency graphs for all numerical and dichotomous features. Possible physiological ranges for the numerical features were defined according to clinical review and expert panel expertise, and values outside these ranges were not included in the analyses. Missing values were imputed with median and mode values from the feature distributions of numerical and dichotomous features, respectively. While other methods were considered, such as multiple imputation, the use of the median and mode was simpler and was deemed sufficient for this work in which the amount of missing data was low.

All numerical features were then standardised using a zero-mean, unit-variance transformation (i.e., using the mean and standard deviations from the feature distributions). This prevents features with relatively small changes in their units of measurement (such as temperature) from being dominated by features with relatively large changes (such as blood pressure), thus ensuring that all features have similar dynamic ranges.

For both imputation and normalisation, the parameters' values (for the median, mode, mean and standard deviation) found for the development dataset were used.

ICU-based model (RS_1)

We used all candidate features derived from the variables acquired during the patients' stay in the ICU to build the first model. A L1-regularised logistic regression using the "glmnet" package in Matlab (Mathworks, Natick, Massachusetts, USA) for predicting the compound outcome (in-hospital death or re-admission to ICU). L1-regularisation shrinks the less important features' coefficients to zero thus effectively removing those features that are deemed to be uninformative to the outcome variable. We estimated the regularisation parameter using LASSO (Least Absolute Shrinkage and Selection Operator) regression in a 5-fold cross-validation on the development set.

Vital-sign based model (RS_2)

For the second scoring system, RS_2, we used the vital signs (heart rate, respiratory rate, systolic blood pressure, oxygen saturation, and temperature) recorded in hospital after discharge from the ICU. For this model, rather than a *supervised learning approach*, we considered a novelty detection method (or one-class classification method), which does not require an outcome variable for development (2). This is useful where event rates are extremely low, or total sample sizes are constrained. Similar approaches have been used in our previous research (3–6) and underpin a commercial, clinically-used system, described in (7).

We considered the construction of a multivariate, (possibly) multimodal model of normality, based on the vital-sign observation sets recorded during the 24 hours that preceded discharge home. If the patient had not died, was not re-admitted to the ICU and was not discharged by the 14th post-ICU day, we used recorded data on that day. These observation sets are thus assumed to contain the vital-sign values from the most stable period of the patient's hospital stay, because these data were acquired when patients are at their most "stable" after discharge from ICU. This set of "normal" data contains $N = 1{,}082$ 5-dimensional vital-sign vectors, $\mathbf{X} = \{\mathbf{x_1}, \dots, \mathbf{x_N}\} \in \mathbb{R}^5$, which were subsequently used for the construction of our model of normality.

A kernel density estimate, or KDE (2) was used to estimate the probability density function (pdf) of the set of five vital signs. This is in a non-parametric technique where no *a priori* assumptions about the form of the underlying probability distribution are made. Our notation follows that reported previously (5, 6). The data pdf $p(\mathbf{x})$ was estimated using the $N = 1{,}082$ set of observations as shown in (SM1-1).

$$p(\mathbf{x}|\mathbf{x}_i, \sigma) = \frac{1}{N(2\pi)^{D/2}\sigma^D} \sum_{i=1}^{N} \exp\left(\frac{-|\mathbf{x}-\mathbf{x}_i|^2}{2\sigma^2}\right) \qquad \text{(SM1-1)}$$

This is a weighted sum of Gaussian kernels, each with identical variance σ^2 (i.e., isotropic kernels), centred on the observation sets $\mathbf{x_1}, \dots, \mathbf{x_N}$.

The nearest-neighbour method was used to estimate the variance. Briefly, this method involves determining the squared Euclidean distance (Δ) for each observation i to its 10 nearest neighbours (*NNs*), as shown in (SM1-2).

$$\Delta_i = \frac{1}{10} \sum_{j \in \{NNs\}} \left\| \mathbf{x}_i - \mathbf{x}_j \right\| \qquad \text{(SM1-2)}$$

This quantity, Δ, is then used to estimate the variance σ^2, as shown in (SM1-3).

$$\sigma = \frac{1}{N} \sum_{i=1}^{N} \Delta_i \qquad \text{(SM1-3)}$$

Estimation of the underlying pdf of normal vital-sign data provides a means of quantifying the degree to which a given set of observations is abnormal. The likelihood $p(\mathbf{x}|\mathbf{x}_i, \sigma)$, a measure which represents the probability of observing a set of measurements given a pdf, can be used for this purpose. Thus, we define the novelty score as in (SM1-4).

$$z(\mathbf{x}) = -\log p(\mathbf{x}|\mathbf{x}_i, \sigma) \qquad \text{(SM1-4)}$$

For normal data, the new observation $\mathbf{x}$ will be similar to previously-seen normal observations $\mathbf{x}_i$, and so the likelihood will be high. Consequently, the negative log-likelihood will be low, and so the novelty score $z(\mathbf{x})$ will be low. Conversely, for abnormal data, the data will be dissimilar and the likelihood will be low, and consequently the novelty score $z(\mathbf{x})$ will be high. This procedure ensures that the

novelty score can be interpreted as most early warning scores (EWS), in that high scores are associated with higher abnormality of the vital signs.

In short, this model allows the generation of a score corresponding to an assessment of whether a vital-sign set should be deemed "stable" with respect to the development dataset (i.e., the observation sets used for training the model). By extension, it further allows the model to estimate the degree of abnormality of a given vital-sign observation set.

Risk Score Index (RSI)

An overall risk score, the risk score index (RSI), was subsequently determined using a simple time-dependent linear combination of the two constituent risk scores, such that:

$$\mathrm{RSI} = \beta\left[\left(1 - \frac{t}{Tmax}\right)\mathrm{RS}_1\right] + \left[\left(\frac{t}{Tmax}\right)\mathrm{RS}_2\right] \qquad \text{(Equation 1)}$$

where t corresponds to the elapsed time (in hours) since the patient was discharged from ICU and has a maximum value of $Tmax$ hours. The time-dependent weighting function allows the contribution of RS_1 to gradually decrease with time after discharge from the ICU. The patient's risk of future adverse events becomes increasingly determined by the values of the vital-sign measurements taken on the acute ward (i.e., RS_2). The parameter β is used to adjust the weight of RS_1 with respect to the time since discharge from ICU, and the parameter $Tmax$ corresponds to the time at which RS_1 no longer affects RSI; i.e., when $t \geq Tmax$, then $\mathrm{RSI} = \mathrm{RS}_2$. The parameters β and $Tmax$ were determined using a grid-search algorithm with 3-fold cross-validation, by defining a grid of possible values of $\beta = [0.1,\ 0.2,\ 0.3, \dots, 200]$ and $Tmax = [12, 24, 36, \dots, 336]$. We selected the values of β and $Tmax$ that corresponded to the highest mean area under the curve of the receiver operating characteristic (AUROC) value across all cross-validation folds, using the compound outcome of in-hospital death or ICU re-admission within the next 24 hours of a vital-sign observation.

During the development of RSI, values of $\beta = 100.2$ and $Tmax = 96$ hours, for the linear time-dependent weighting function (see Equation 1), were obtained and used to calculate the risk scoring index.

Model validation and statistical analysis

To validate the first model, RS_1, its discrimination and calibration were analysed. Discrimination is defined as the ability of the model to separate non-event patients from patients who had an adverse event after ICU discharge, and was assessed

using the AUROC metric. Calibration assesses the degree of correspondence between the estimated probability of occurrence of an adverse event and that actually observed. This was tested using a goodness of fit test, the Hosmer-Lemeshow "C" statistic (8). When the predicted probability of adverse events of the prognostic model differs significantly from the observed pattern, the calibration ability of the model is deemed to be poor. As the Hosmer-Lemeshow test does not measure the magnitude of miscalibration and is sensitive to sample size (9), calibration was also assessed with the Brier score and Cox's calibration regression. The latter assesses the degree of miscalibration by fitting a logistic regression of observed survival to the predicted log-odds of survival from the risk model (10). The performance of the first model was examined both for the compound outcome and each adverse event (in-hospital death and ICU re-admission) individually. The ability of the first model to predict future adverse events at increasing intervals from ICU discharge was also examined by calculating the AUROC for future events by day after discharge (up to 120 days).

The final model, RSI, was validated using the AUROC for the derived outcome of in-hospital death or ICU re-admission within the next *N* hours of a vital-sign observation/measurement performed after ICU discharge, in line with previous studies for evaluating EWS systems (11, 12). We evaluated the model for different values of *N*, with $N = [12, 24, 36, 48, 72]$ hours. We note that in this case the AUROC represents how well the scoring system RSI discriminates between observation sets followed by an adverse event and those with no subsequent adverse outcome within the next *N* hours. Therefore, the unit of analysis is a vital-sign set rather than a patient-admission, as performed for the validation of the first model.

We also considered each individual adverse event separately. To further understand the feasibility of implementation of the risk scoring systems in this setting, the burden of observation sets "triggered" for every correctly identified observation followed by an adverse event within 24 hours by the risk scoring system was also evaluated.

We report the cross-validation results using the development dataset. This gives an estimate of how our models perform on a random set of samples from the OUH Trust that were not used for developing the model. We also report the external validation results using data from the RBH Trust. Confidence intervals were estimated using bootstrap confidence intervals via percentiles with 500 samples (13).

References

1. GOV.UK (National Statistics): English indices of deprivation 2015. *Accessed from https//www.gov.uk/government/statistics/english-indices-of-deprivation-2015; Accessed January 2018*
2. Pimentel MAF, Clifton DA, Clifton L, et al.: A review of novelty detection. *Signal Processing* 2014; 99:215–249

3. Watkinson PJ, Barber VS, Price JD, et al.: A randomised controlled trial of the effect of continuous electronic physiological monitoring on the adverse event rate in high risk medical and surgical patients. *Anaesthesia* 2006;

4. Clifton DA, Wong D, Clifton L, et al.: A large-scale clinical validation of an integrated monitoring system in the Emergency Department. *IEEE J Biomed Heal Informatics* 2013;

5. Johnson AEW, Burgess J, Pimentel MAF, et al.: Physiological trajectory of patients pre and post ICU discharge. In: 2014 36th Annual International Conference of the IEEE Engineering in Medicine and Biology Society. IEEE; 2014. p. 3160–3163.

6. Pimentel MAF, Clifton DA, Clifton L, et al.: Modelling physiological deterioration in post-operative patient vital-sign data. *Med Biol Eng Comput* 2013;

7. Orphanidou C, Clifton D, Shahab Khan, et al.: Telemetry-based vital sign monitoring for ambulatory hospital patients. In: 2009 Annual International Conference of the IEEE Engineering in Medicine and Biology Society. IEEE; 2009. p. 4650–4653.

8. Lemeshow S, Hosmer DW: A review of goodness of fit statistics for use in the development of logistic regression models. *Am J Epidemiol* 1982;

9. Kramer AA, Zimmerman JE: Assessing the calibration of mortality benchmarks in critical care: The Hosmer-Lemeshow test revisited*. *Crit Care Med* 2007; 35:2052–2056

10. Cox DR: Two further applications of a model for binary regression. *Biometrika* 1958; 45:562–565

11. Prytherch DR, Smith GB, Schmidt PE, et al.: ViEWS-Towards a national early warning score for detecting adult inpatient deterioration. *Resuscitation* 2010; 81:932–937

12. Badriyah T, Briggs JS, Meredith P, et al.: Decision-tree early warning score (DTEWS) validates the design of the National Early Warning Score (NEWS). *Resuscitation* 2014; 85:418–423

13. DiCiccio TJ, Efron B: Bootstrap confidence intervals. *Stat Sci* 1996;
