# Supplementary material for: Development of an enhanced scoring system to predict ICU readmission or in-hospital death within 24 hours using routine patient data from two NHS Foundation Trusts
Source: BMJ Open. 2024 Apr 12;14(4):e074604. doi: 10.1136/bmjopen-2023-074604 (PMC11029184; doi:10.1136/bmjopen-2023-074604)
Supplement: Supplementary data [file bmjopen-2023-074604supp002.pdf]

Additional file 2 – Comparative scoring systems

In this section, we described the details of other scoring systems used in this study and how they were implemented using the information included in datasets. We also detail the components and weightings of the individual scores, as they were implemented in this work.

The early warning score (EWS) systems evaluated in this study include the modified EWS, or MEWS (1), the standardised EWS, or SEWS (2), the National EWS, or NEWS (3, 4) and the centile-based EWS, or CEWS (5). Their components and weightings are shown in tables SM2-1, SM2-2, SM2-3 and SM2-4, respectively. The core set of variables that are used in these scoring systems comprises heart rate (measured in beats per minute), respiratory rate (in breaths per minute), systolic and diastolic blood pressure (in mmHg), body temperature (in °C), oxygen saturation (denoted “SpO_2”, in %), an assessment of neurological status (using the Alert-Verbal-Painful-Unresponsive or AVPU scale), a flag for whether the patient was receiving supplementary oxygen at the time of the SpO_2 measurement (denoted “Inspired O_2”), and the age to the patient at admission to hospital. At each observation set (or measurement timestamp), in case of an incomplete vital-sign observation set, we used the most recent value of each variable. Remaining missing data were dealt with as with the proposed scoring system (see section 2 in the main manuscript).

Table SM2-1. Modified Early Warning Score (see main text for units of the variables included).

Table SM2-2. Standardised Early Warning Score (see main text for units of the variables included).

Table SM2-3. National Early Warning Score (see main text for units of the variables included).

Table SM2-4. Centile-based Early Warning Score (see main text for units of the variables included).

Table SM2-1.

Modified Early Warning Score (MEWS)

	Score						
Variable	3	2	1	0	1	2	3
Heart rate	≤ 39	40 – 50	51 – 59	60 – 100	101 – 110	111 – 129	≥ 130
Resp. rate		≤ 8		9 – 14	15 – 20	21 – 29	≥ 30
Temperature		≤ 35.0		35.1 – 38.0		38.1 – 39.5	≥ 39.6
Systolic BP	≤ 69	70 – 80	81 – 100	101 – 149	150 – 169	170 – 179	≥ 180
AVPU scale				A	V	P	U

Table SM2-2.

Standardised Early Warning Score (SEWS)

	Score						
Variable	3	2	1	0	1	2	3
Heart rate	≤ 29	30 – 39	40 – 49	50 – 99	100 – 109	110 – 129	≥ 130
Resp. rate	≤ 8			9 – 20	21 – 30	31 – 35	≥ 36
Temperature	≤ 33.9	34.0 – 34.9	35.0 – 35.9	36.0 – 37.9	38.0 – 38.9	≥ 39.0	
Systolic BP	≤ 69	70 – 79	80 – 99	100 – 199		≥ 200	
SpO_2	≤ 84	85 – 89	90 – 92	≥ 93			
AVPU scale				A	V	P	U

Table SM2-3.

National Early Warning Score (NEWS)

	Score						
Variable	3	2	1	0	1	2	3
Heart rate	≤ 40		41 – 50	51 – 90	91 – 110	111 – 130	≥ 131
Resp. rate	≤ 8		9 – 11	12 – 20		21 – 24	≥ 25
Temperature	≤ 35.0		35.1 – 36.0	36.1 – 38.0	38.1 – 39.0	≥ 39.1	
Systolic BP	≤ 90	91 – 100	101 – 110	111 – 219			≥ 220
SpO_2	≤ 91	92 – 93	94 – 95	≥ 96			
Inspired O_2		Any O_2		Air			
AVPU scale				A			V, P, U

Table SM2-4.

Centile-based Early Warning Score (CEWS)

	Score						
Variable	3	2	1	0	1	2	3
Heart rate	≤ 42	43 – 49	50 – 53	54 – 104	105 – 112	113 – 127	≥ 128
Resp. rate	≤ 7	8 – 10	11 – 12	13 – 21	22 – 23	24 – 28	≥ 29
Temperature	≤ 35.4		35.5 – 35.9	36.0 – 37.3	37.4 – 38.3		≥ 38.4
Systolic BP	≤ 83	84 – 90	91 – 100	101 – 157	158 – 167	168 – 184	≥ 185
SpO_2	≤ 84	85 – 90	91 – 93	≥ 94			
Inspired O_2		Any O_2		Air			
AVPU scale				A	V		P, U

References

1. Gardner-Thorpe J, Love N, Wrightson J, et al.: The value of Modified Early Warning Score (MEWS) in surgical in-patients: A prospective observational study. *Ann R Coll Surg Engl* 2006; 88:571–575
2. Paterson R, MacLeod DCD, Thetford D, et al.: Prediction of in-hospital mortality and

length of stay using an early warning scoring system: clinical audit. *Clin Med J R Coll Physicians London* 2006; 6:281–284

3. Smith GB, Prytherch DR, Meredith P, et al.: The ability of the National Early Warning Score (NEWS) to discriminate patients at risk of early cardiac arrest, unanticipated intensive care unit admission, and death. *Resuscitation* 2013; 84:465–470
4. Royal College of Physicians (RCP): National Early Warning Score (NEWS). *Stand Assess acute-illness Sev NHS* 2012; 47
5. Watkinson PJ, Pimentel MAF, Clifton DA, et al.: Manual centile-based early warning scores derived from statistical distributions of observational vital-sign data. *Resuscitation* 2018; 129:55–60
