# Supplementary material for: Development of an enhanced scoring system to predict ICU readmission or in-hospital death within 24 hours using routine patient data from two NHS Foundation Trusts
Source: BMJ Open. 2024 Apr 12;14(4):e074604. doi: 10.1136/bmjopen-2023-074604 (PMC11029184; doi:10.1136/bmjopen-2023-074604)
Supplement: Supplementary data [file bmjopen-2023-074604supp003.pdf]

Additional file 3 – Additional results

In this section, we describe in more detail some of the results of the study.

ICU-based model (RS_1)

The features computed from the data acquired during the patients' ICU stay that were selected for the first model are detailed in Table SM3-1. A total of 45 features were included in the model from the 161 candidate features.

Calibration plots of the model for both development and validation sets are shown in Figure SM3-1. Figure SM3-2 shows the AUROC values achieved by the first model (RS_1) for predicting the adverse event (combined outcome) within an increasingly high number of days after discharge from ICU. The curves of the AUROC values for each outcome, in-hospital death or ICU re-admission, separately, are also shown.

Table SM3-1. Features computed from information acquired during the patients' ICU stay used in the first model, and the corresponding odds ratio and 95% confidence interval (95% CI) for the odds ratio. The normalisation parameters for each feature (mean and standard deviation, SD) are also shown.

Figure SM3-1. Calibration curves for the first model (RS_1) for predicting a future adverse event after ICU discharge. The left-hand panel shows the calibration curve on the development dataset, the right-hand panel shows the calibration curve of the developed model on the validation dataset. The grey line depicts an ideal calibration, representing a perfect correspondence between the observed events and the predicted events for each decile of score.

Figure SM3-2. Performance of the first model (as given by the AUROC) for prediction of cumulative in-hospital deaths, cumulative ICU re-admissions, and cumulative adverse events up to a cut-off day after ICU discharge. The left-hand panel shows the results using the development dataset, and the right-hand panel shows the performance using the validation dataset. The bottom graphs show the proportion of each event (N) over time for the three outcomes considered.

Vital-sign based model (RS_2)

For the second scoring system, we considered the construction of a multivariate, (possibly) multimodal model of normality, based on the vital-sign observation sets recorded during the 24 hours that preceded discharge home or during the last post-ICU day with recorded data (which corresponds to the 14th day) if the patient had not

been discharged, died or re-admitted to the ICU. These observation sets contain the vital signs from the most stable period of the patient's stay, because these data were acquired when patients are at their most "normal" state after discharge from ICU. This set of "normal" data contains $N = 1{,}082$ 5-dimensional vital-sign vectors. Table SM3-2 gives the corresponding means and standard deviations of this subset of data, used to train the model.

Table SM3-2. Vital signs' mean and standard deviation (SD) values for observations recorded in during the most "stable" period considered.

Figure SM3-1.

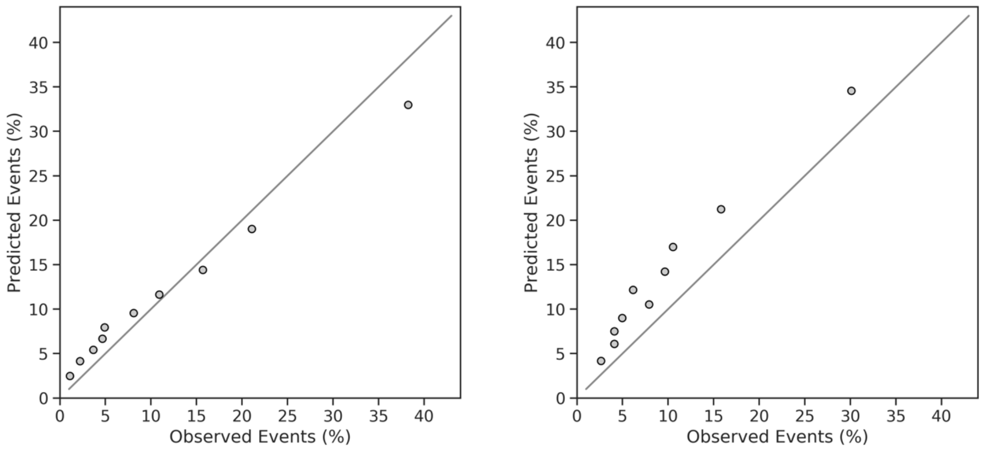
Predicted Events (%)
Observed Events (%)
0
5
10
15
20
25
30
35
40
Predicted Events (%)
Observed Events (%)

Figure SM3-2.

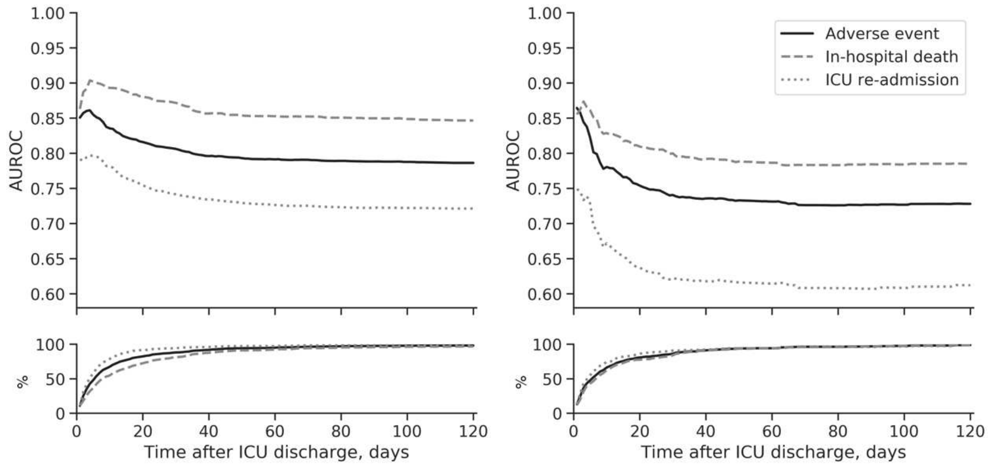
AUROC
%
Time after ICU discharge, days
Adverse event
In-hospital death
ICU re-admission

Table SM3-1.

Feature description	Mean (SD)		Odds ratio	95% CI
**Demographic and administrative information (**3 features**)**				
Age at admission to ICU	59	(17)	1.305	1.196 to 1.425
Time spend in the ICU, in days	4.3	(19.5)	1.134	1.072 to 1.199
Time from acute hospital admission and ICU admission, in days	4.2	(7.4)	1.038	0.964 to 1.119
**Vital signs related (**13 features**)**				
Last ventricular heart rate	86	(17)	1.135	1.024 to 1.259
Highest respiratory rate in the last 72 hours	26	(7)	1.129	1.033 to 1.235
Lowest ventricular heart rate in the last 72 hours	70	(16)	1.056	0.927 to 1.202
Highest ventricular heart rate in the first 24 hours	105	(22)	1.039	0.944 to 1.143
Last respiratory rate	18	(5)	1.026	0.943 to 1.116
Lowest ventricular heart rate in the first 24 hours	72	(17)	1.023	0.901 to 1.161
Highest SpO_2 recorded using pulse oximetry in the last 72 hours	100	(1)	1.016	0.944 to 1.094
Lowest respiratory rate in the first 24 hours	12	(3)	1.002	0.925 to 1.085
Lowest temperature in the first 24 hours	37.0	(0.7)	0.984	0.906 to 1.068
Highest temperature in the first 24 hours	35.5	(0.8)	0.981	0.889 to 1.083
Last mean arterial blood pressure	91	(16)	0.921	0.853 to 0.995
Lowest temperature in the last 72 hours	35.5	(0.7)	0.917	0.831 to 1.011
Last total GCS recorded	14	(1)	0.854	0.805 to 0.906
**Laboratory test results related (**24 features**)**				
Last plasma urea	9.1	(7.1)	1.313	1.186 to 1.453
Last plasma sodium	139	(4)	1.152	1.073 to 1.237
Highest INR in the first 24 hours	1.23	(0.48)	1.063	0.982 to 1.151
Last plasma bilirubin	12.8	(14.8)	1.052	0.984 to 1.124
Highest pH in the first 24 hours	7.43	(0.06)	1.051	0.971 to 1.138
Highest C-reactive protein in the first 24 hours	91	(55)	1.050	0.939 to 1.173
Last plasma lactate	1.16	(0.71)	1.028	0.957 to 1.103
Last INR	1.13	(0.35)	1.027	0.951 to 1.111
Last C-reactive protein	89	(54)	1.009	0.904 to 1.126
Highest platelet count in the first 24 hours	200	(109)	1.006	0.929 to 1.089
Last plasma sodium was in the extremes of the development set range (> or <)	-	-	1.003	0.831 to 1.211
Last plasma creatinine	116	(130)	0.993	0.735 to 1.342
The variability of $AaDO_2$ in the last 72 hours	6.1	(5.1)	0.990	0.908 to 1.080
Highest weight in the first 24 hours	77	(20)	0.989	0.909 to 1.076
Last haematocrit	32	(5)	0.989	0.881 to 1.110
Highest creatinine in the last 24 hours	124	(142)	0.975	0.744 to 1.278

Lowest haemoglobin in the first 24 hours	9.6	(2.0)	0.963	0.864 to 1.075
Highest white blood cell count in the first 24 hours	13.9	(7.0)	0.962	0.893 to 1.036
Lowest plasma potassium in the first 24 hours	3.8	(0.5)	0.951	0.879 to 1.030
Highest plasma creatinine in the last 72 hours	134	(153)	0.950	0.752 to 1.200
Total urine output in the first 24 hours	1772	(1260)	0.937	0.842 to 1.043
Last PaO_2/FiO_2 ratio	43.7	(15.8)	0.904	0.826 to 0.990
Last plasma albumin	28	(6)	0.870	0.798 to 0.949
Total urine output in the last 24 hours	2007	(1293)	0.781	0.703 to 0.867
**Procedures and treatments related (**5 features**)**				
If enteral feeding was used at any point during ICU stay	-	-	1.119	0.946 to 1.323
If a central venous catheter was present at ICU discharge	-	-	1.118	0.957 to 1.306
If an insulin infusion was used in the last 24 hours	-	-	1.055	0.894 to 1.246
If parenteral feeding was used at any point during ICU stay	-	-	1.044	0.799 to 1.363
A dysrhythmia at any point during ICU stay	-	-	1.001	0.999 to 1.003

Table SM3-2.

Vital sign	Mean (SD)
**Heart rate, beats per min.**	83 (14)
**Respiratory rate, breaths per min.**	17 (2)
**Systolic blood pressure, mmHg**	129 (19)
**SpO_2, %**	97 (3)
**Temperature, C**	36.3 (0.5)
