# Supplementary material for: Development of an enhanced scoring system to predict ICU readmission or in-hospital death within 24 hours using routine patient data from two NHS Foundation Trusts
Source: BMJ Open. 2024 Apr 12;14(4):e074604. doi: 10.1136/bmjopen-2023-074604 (PMC11029184; doi:10.1136/bmjopen-2023-074604)
Supplement: Author's manuscript [file bmjopen-2023-074604.draft_revisions.pdf]

BMJ Open is committed to open peer review. As part of this commitment we make the peer review history of every article we publish publicly available.

When an article is published we post the peer reviewers' comments and the authors' responses online. We also post the versions of the paper that were used during peer review. These are the versions that the peer review comments apply to.

The versions of the paper that follow are the versions that were submitted during the peer review process. They are not the versions of record or the final published versions. They should not be cited or distributed as the published version of this manuscript.

BMJ Open is an open access journal and the full, final, typeset and author-corrected version of record of the manuscript is available on our site with no access controls, subscription charges or pay-per-view fees (http://bmjopen.bmj.com).

If you have any questions on BMJ Open's open peer review process please email info.bmjopen@bmj.com

BMJ Open

Early warning score using electronically-held data for patients discharged from intensive care units

Journal:	*BMJ Open*
Manuscript ID	bmjopen-2023-074604
Article Type:	Original research
Date Submitted by the Author:	11-Apr-2023
Complete List of Authors:	Pimentel, Marco; University of Oxford, Department of Engineering Science<br>Johnson, Alistair; Massachusetts Institute of Technology, Institute of Medical Engineering & Science<br>Darbyshire, Julie; Oxford University, Department of Primary Care Health Sciences<br>Tarassenko, Lionel; University of Pittsburgh, Engineering Science<br>Clifton, David A.; Univ Oxford, Department of Engineering Science<br>Walden, Andrew; Royal Berkshire NHS Foundation Trust<br>Rechner, Ian; Royal Berkshire NHS Foundation Trust<br>Young, Duncan; Oxford University, Nuffield Department of Clinical Neurosciences<br>Watkinson, Peter; University of Oxford, Nuffield Department of Clinical Neurosciences
Keywords:	INTENSIVE & CRITICAL CARE, Clinical Decision-Making, Health informatics < BIOTECHNOLOGY & BIOINFORMATICS

SCHOLARONE™
Manuscripts

I, the Submitting Author has the right to grant and does grant on behalf of all authors of the Work (as defined in the below author licence), an exclusive licence and/or a non-exclusive licence for contributions from authors who are: i) UK Crown employees; ii) where BMJ has agreed a CC-BY licence shall apply, and/or iii) in accordance with the terms applicable for US Federal Government officers or employees acting as part of their official duties; on a worldwide, perpetual, irrevocable, royalty-free basis to BMJ Publishing Group Ltd ("BMJ") its licensees and where the relevant Journal is co-owned by BMJ to the co-owners of the Journal, to publish the Work in this journal and any other BMJ products and to exploit all rights, as set out in our licence.

The Submitting Author accepts and understands that any supply made under these terms is made by BMJ to the Submitting Author unless you are acting as an employee on behalf of your employer or a postgraduate student of an affiliated institution which is paying any applicable article publishing charge ("APC") for Open Access articles. Where the Submitting Author wishes to make the Work available on an Open Access basis (and intends to pay the relevant APC), the terms of reuse of such Open Access shall be governed by a Creative Commons licence – details of these licences and which Creative Commons licence will apply to this Work are set out in our licence referred to above.

Other than as permitted in any relevant BMJ Author's Self Archiving Policies, I confirm this Work has not been accepted for publication elsewhere, is not being considered for publication elsewhere and does not duplicate material already published. I confirm all authors consent to publication of this Work and authorise the granting of this licence.

For peer review only

Title page

Early warning score using electronically-held data for patients discharged from intensive care units

Authors:

Marco AF Pimentel, Institute of Biomedical Engineering, Department of Engineering Science, University of Oxford, Oxford, UK

Alistair Johnson, Institute of Medical Engineering & Science, Massachusetts Institute of Technology, Cambridge, Massachusetts, USA.

Julie L Darbyshire, Nuffield Department of Clinical Neurosciences, University of Oxford, Oxford, UK

Lionel Tarassenko, Institute of Biomedical Engineering, Department of Engineering Science, University of Oxford, Oxford UK

David A Clifton, Institute of Biomedical Engineering, Department of Engineering Science, University of Oxford, Oxford UK

Andrew Walden, Intensive Care Unit, Royal Berkshire NHS Foundation Trust, Reading, UK

Ian Rechner, Intensive Care Unit, Royal Berkshire NHS Foundation Trust, Reading, UK

J Duncan Young, Nuffield Department of Clinical Neurosciences, University of Oxford, Oxford, UK

Peter J Watkinson, Nuffield Department of Clinical Neurosciences, University of Oxford, Oxford, UK

Corresponding author:
Julie L Darbyshire
Nuffield Department of Clinical Neurosciences,
Kadoorie Centre for Critical Care Research & Education,
John Radcliff Hospital
Oxford
OX3 9DU
UK
julie.darbyshire@ndcn.ox.ac.uk

Running head:
Early warning scores after ICU

For peer review only

<u>Descriptor number:</u>
4.6

<u>Word count:</u>
Body: ~3,300
Abstract: 300
Tables: 3
Figures: 2

<u>At a Glance Commentary:</u>

Scientific Knowledge on the Subject
Early Warning Score (EWS) systems are used to identify hospitalised patients at risk of adverse events. These systems almost all use vital signs to determine this risk. Adding further data from electronic patient records may improve the performance of these systems. Patients discharged from an intensive care unit have both a comprehensive electronic patient record and a high risk of future adverse events, so they are an ideal patient group to test if additional data improves the system performance.

What This Study Adds to the Field
The study shows a scoring system incorporating data from a patient's stay in an intensive care unit has better performance than typically-used EWS systems based on vital signs alone. The score developed in this study uses a machine learning method to estimate future risk.

This article has additional files.

For peer review only

Abstract

Rationale. Intensive care units (ICUs) admit the most severely ill patients. Once these patients are discharged from the ICU to a step-down ward, they continue to have their vital signs monitored by nursing staff, with early warning score (EWS) systems being used to identify those at risk of deterioration.

Objectives. We report the development and validation of an enhanced continuous scoring system for predicting adverse events, which combines vital signs measured routinely on acute care wards (as used by most EWSs) with a risk score of a future adverse event calculated on discharge from ICU.

Methods. A modified Delphi process identified common, and candidate variables frequently collected and stored in electronic records as the basis for a 'static' score of the patient's condition immediately after discharge from the ICU. L1-regularised logistic regression was used to estimate the in-hospital risk of future adverse event. We then constructed a model of physiological normality using vital-sign data from the day of hospital discharge, which is combined with the static score and used continuously to quantify and update the patient's risk of deterioration throughout their hospital stay. Data from two NHS Foundation Trusts (UK) were used to develop and (externally) validate the model.

Results. A total of 12,394 vital-sign measurements were acquired from 273 patients after ICU discharge for the development set, and 4,831 from 136 patients in the validation cohort. Outcome validation of our model yielded an area under the receiver operating characteristic curve (AUROC) of 0.724 for predicting ICU re-admission or in-hospital death within 24h. It showed an improved performance with respect to other competitive risk scoring systems, including the National EWS (NEWS, 0.653).

Conclusions. We showed that a scoring system incorporating data from a patient's stay in ICU has better performance than commonly-used EWS systems based on vital signs alone.

Keywords: early warning score; intensive care; physiological deterioration; patient monitoring.

Strengths and Limitations

* Over 17,000 vital sign measurements from >400 patients included in the cohort

For peer review only - http://bmjopen.bmj.com/site/about/guidelines.xhtml

* Variable selection informed by systematic review and Delphi process prior to data extraction
* Data extraction variables limited to those available
* Performance assessed using common methology for early warning score systems
* Compound outcome (including in-hospital mortality and ICU re-admission) limits prediction of inevitable death and improves prediction of preventable deaths

For peer review only

Introduction

Patients hospitalised for acute conditions can suffer adverse events such as cardiac arrests or unplanned admission to a higher-acuity area. These events are usually preceded by changes in vital signs some hours before (1–5). Early detection of these changes might prevent some of the subsequent adverse events, so many simple Early Warning Score (EWS) systems have been developed and deployed clinically (6–8). EWS systems are now recommended to be used as part of routine care by the UK National Institute for Health and Clinical Excellence (9–11) and are widely used in other healthcare settings.

EWS systems initially used purpose-designed paper charts (12, 13). An increasing number of healthcare providers now use electronic systems (14, 15) to gather physiological data and calculate scores (16). With the increasing use of electronic patient records, there is scope to develop improved EWS systems that include many more variables than are currently used, potentially leading to increasing predictive accuracy by using more complex algorithms to calculate scores (17).

We hypothesise that an EWS system could better predict adverse events if it included variables from a patient's electronic medical record. As a proof of concept, we studied patients discharged to acute care wards from intensive care units (ICUs). These patients have a detailed electronic patient record (EPR) containing data about their ICU stay, and typically having a high rate of adverse events occurring after discharge to a ward. In addition, there is a large body of literature describing variables that are correlated with adverse events in these patients.

This study describes the development of an enhanced scoring system for predicting adverse events in patients discharged from ICUs which combines (i) routine vital signs measured on acute care wards (as used by most EWS systems) with (ii) a risk score of a future adverse event calculated on discharge from ICU. The study further validates the ability of the proposed scoring system and other published EWS systems to identify patients at risk of death or re-admission to ICU.

Materials and Methods

This study is reported in line with the TRIPOD statement (18).

Regulatory approvals

The project had UK Health Research Authority Confidentiality Advisory Group approval (ref: ECC 7-05(f)/2011), National Research Ethics Service Committee approval (ref: 11/SC/0440) for retrospective data collection and analysis, and National Research Ethics Service Committee approval (ref: 12/SC/0357) for prospective data collection and analysis.

For peer review only - http://bmjopen.bmj.com/site/about/guidelines.xhtml

Data sources

Data from two NHS Foundation Trusts (UK) were used in this study. The Oxford University Hospitals NHS Foundation Trust (OUH) has two adult general ICUs. The Royal Berkshire Hospital NHS Foundation Trust (RBH) in Reading, UK, has a single adult general ICU.

We used routinely-collected data, stored in the ICU computerised information systems (CIS). The OUH and RBH CIS contain all measurements of physiological status, and other relevant clinical information such as patient demographics; details of treatments and interventions; and laboratory test results recorded during a patient's ICU stay. An average of 655 data items are recorded daily for each patient. All the ICUs in this study use a Philips Healthcare CIS (Philips Healthcare, Eindhoven, Netherlands). In addition we used the linked datasets submitted to the UK national comparative audit for ICUs, or ICNARC CMP (19).

During the study period, neither organisation had electronic recording of vital signs for patients in acute care wards outside the ICUs. Vital-sign data were collected prospectively for recruited patients in both organisations for the first 14 days after discharge from ICU. The physiological data from each patient's paper observation chart were entered into an electronic database. Data were double-entered in 55% of patients to confirm the accuracy of data entry.

All analyses were conducted on anonymised data.

Participants

All completed adult admissions to OUH ICUs from June 2006 to December 2015 and to the RBH ICU from April 2010 to December 2015 were used to develop the ICU discharge score. Admissions were only considered when (i) patients were discharged alive from ICU and (ii) the discharge status of the patient episode (or hospital admission) was known (or recorded). We excluded patients discharged from ICU for palliative care or transferred to another organisation. Vital signs data on wards were acquired prospectively after discharge from ICU for patients in OUH between April 2013 and December 2014, and in RBH from October 2013 and December 2014.

After exclusion criteria were applied, we split the database into "development" and "validation" sets by organisation, such that (1) we developed and evaluated the model using data from all valida admissions within the OUH group using cross-validation methods, and (2) we performed an external validation of the model using data from all valid admissions within the RBH group.

For peer review only

Outcomes

The primary outcome was the first occurrence of either in-hospital death or re-admission to the ICU. For the evaluation of EWS systems, we considered the compound outcome of in-hospital death or ICU re-admission within the next *N* hours of a vital-sign observation, in line with previous studies (20, 21). For the primary outcome, we set $N = 24$ hours. We have also evaluated the systems for different values of *N* (as detailed below). Secondary outcomes were in-hospital death or re-admission to ICU, individually.

Predictors

We used a conceptual model in which we estimated the risk of a patient experiencing an adverse event at the point of discharge from ICU from variables recorded during their stay in the ICU. After discharge from ICU, the patient's risk of experiencing an adverse event within the following 24h was calculated using both ward-recorded vital signs and the risk calculated at ICU discharge.

<u>ICU-based feature representation</u>:
We used an evidence-based technique to select candidate variables to calculate the risk of deterioration at ICU discharge. We systematically reviewed of studies reporting a significant ($p < 0.05$) association between a variable recorded during an ICU stay and either in-hospital death or ICU re-admission. The resulting list of candidate variables was reviewed by a panel of five clinical experts in a modified Delphi process who added other variables they expected to be predictive of adverse events. Candidate variables were then limited to those available in our electronic databases. These were either "static" variables (mainly based on demographic information) or time-varying variables recorded repeatedly throughout the patient's stay in the ICU (see Figure 1). To determine the risk of future compound outcome after discharge from the ICU, we derived a total of 161 candidate features from all candidate variables, which were then used for building a prediction model.

<u>Post-ICU feature representation</u>:
All vital-sign observations recorded for 14 days after discharge from ICU were considered for analysis. Each set of vital signs includes heart rate, systolic blood pressure, respiratory rate, body temperature, neurological status assessment using the Alert-Verbal-Painful-Unresponsive (AVPU) scale, peripheral oxygen saturation (SpO_2), a record of whether the patient was receiving supplemental oxygen at the time of the SpO_2 measurement, and the date and time of the observation. Vital-sign measurements are typically recorded every 4 or 6h throughout the patient's stay on the ward (see Figure 1).

The final list of candidate variables and features, and procedures for pre-processing (including dealing with missing data) are further described [see Additional file 1].

For peer review only

Model development

To develop the risk scoring system, our approach assumes that at each vital-sign observation performed after discharge from ICU, the patient's current condition can be characterised (or represented) by a single risk estimate. Immediately after ICU discharge, the patient is assigned a risk score (RS_1) estimated from an ICU-based set of features, which is then updated using the abnormalities in their vital signs recorded during subsequent ward care (RS_2).

We therefore built the first model, RS_1, using the features derived from the variables acquired during the patients' stay in the ICU using the development set. We used a L1-regularised logistic regressor for predicting the compound outcome. For the second scoring system, RS_2, we applied a one-class classification method (22) using the vital-sign data recorded after discharge from the ICU, as described previously (23, 24). Further details of the model development are available [see Additional file 1].

An overall risk score, the risk score index (RSI), was subsequently determined using a simple time-dependent linear combination of the two constituent risk scores, such that:

$$\mathrm{RSI} = \beta\left[\left(1 - \frac{t}{Tmax}\right)\mathrm{RS}_1\right] + \left[\left(\frac{t}{Tmax}\right)\mathrm{RS}_2\right] \qquad \text{(Equation 1)}$$

where β is used to adjust the weight of RS_1 with respect to the time since discharge from ICU, t corresponds to the elapsed time (in hours) since the patient was discharged from ICU and has a maximum value of $Tmax$ hours. Further details of the model development and optimisation of the parameters are available [see Additional file 1].

Model validation and statistical analysis

The discrimination of the first model, RS_1, was assessed using the area under the curve of the receiver operating characteristic (AUROC) metric. Calibration was assessed using a goodness of fit test, the Hosmer-Lemeshow "C" statistic, the Brier score and Cox's calibration regression (25–27). The performance of this first model was examined both for the compound outcome and each adverse event (in-hospital death and ICU re-admission) individually. The ability of RS_1 to predict future adverse events at increasing intervals from ICU discharge was also examined by calculating the AUROC for future events by day after (ICU) discharge (up to 120 days).

The final model (RSI) was validated using the AUROC for the compound outcome of in-hospital death or ICU re-admission within the next *N* hours of a vital-sign measurement recorded after ICU discharge, in line with previous studies for

For peer review only - http://bmjopen.bmj.com/site/about/guidelines.xhtml

evaluating EWS systems (20, 21). We evaluated the model for different values of *N*, with $N = [12, 24, 36, 48, 72]$ hours. We note that in this case the AUROC represents how well the scoring system RSI discriminates between observation sets followed by an adverse event and those with no subsequent adverse outcome within the next *N* hours. Therefore, the unit of analysis is a vital-sign set rather than a patient-admission, as performed for the validation of the first model.

We also considered each individual adverse event separately. To understand better the feasibility of implementating the risk scoring systems in this setting, we also evaluated the burden of observation sets "triggered" by the risk scoring system for every correctly identified observation followed by an adverse event within 24h.

We report the cross-validation results using the development dataset. We also report the external validation results using data from the RBH Trust. Confidence intervals were estimated using bootstrap confidence intervals via percentiles, with 500 samples (28).

Comparison with published risk scoring systems

We compared the performance of our proposed risk scoring index, RSI, with that of each model individually (RS_1 and RS_2), and with that of a number of published and clinically-used EWS systems: the modified EWS, or MEWS (29), the standardised EWS, or SEWS (30), the National EWS, or NEWS (21), and our centile-based EWS, or CEWS (31). We detail the components and weightings of the individual EWS systems in Additional file 2.

Results

The application of inclusion and exclusion criteria to patient-admissions in order to derive the final cohorts in both organisations included in this study is shown in Figure SM3-1 [see Additional file 3]. A total of 8,162 admissions to ICU were included in the development dataset. Vital-sign observations were prospectively collected for 273 patients (from the 8,162 ICU admissions) after discharge from the ICU. The validation dataset included 3,421 ICU admissions, from which 136 had data acquired during the post-ICU period. Table 1 provides a summary of characteristics of the development and validation cohorts derived from the ICU admissions. Both cohorts were similar in terms of demographic and administrative data. The compound outcome rate was slightly higher in the development dataset than that in the validation dataset, considering either all ICU admissions or the sub-group of post-ICU admissions. We also note that the number of in-hospital deaths is higher than the number of re-admissions to ICU. In the development cohort, 12,394 vital-sign observation sets were acquired from the 273 patients after ICU discharge, and 4,831 from the 136 patients in the validation cohort.

For peer review only - http://bmjopen.bmj.com/site/about/guidelines.xhtml

For the first model (RS_1), which estimates the risk of future adverse events immediately after ICU discharge, 45 features from the 161 candidate features identified from the systematic review and expert opinion, were retained in the final model. These are listed Table SM3-1 [see Additional file 3]. They comprise largely measures of cardiac or respiratory physiology, renal and hepatic function, plasma electrolytes, measures of inflammation and measures of treatment intensity. Of note, artificial ventilation during the last 24h of ICU admission was associated with a lower risk of adverse outcome. This is because all the ICUs admit ventilated, elective, post-surgical admissions who have a low risk of adverse events and are discharged within 24h of extubation. Calibration plots of the model for both development and validation sets are shown in Figure SM3-2 [see Additional file 3]. Table 2 summarises the performance for the combined outcome and for either adverse event (in-hospital death or ICU re-admission) considered individually.

Table 3 shows the performance of RSI and other baseline scoring systems for predicting observation sets followed by in-hospital death or ICU re-admission within the following 24h. The proposed scoring system showed an increased discrimination ability to predict adverse events within 24h with respect to the other risk scoring systems considered in this study. Using the external validation dataset (RBH), RSI gave an AUROC of 0.724 (95% CI of 0.704-0.741), versus 0.653 (0.621-0.683) for NEWS, and 0.672 (0.648-0.695) for CEWS. Figure 2 shows the AUROC values of the risk scoring systems for predicting the compound outcome within 12, 24, 36, 48, and 72h of a vital-sign measurement. The proposed RSI system consistently shows superior discrimination for each derived outcome.

Discussion

There are many variants of the original EWS system (6–8), including systems designed for specific patient groups such as children (32, 33) and patients in high-dependency units (34). These systems typically use vital signs to determine the level of risk of an adverse event. Vital signs are used because they are easily-acquired variables, regularly measured in clinical practice, and have a long history of being used to track patients' progression over time. However, point estimates of the risk of future deterioration from single vital-sign measurement sets assume "normal" values that may not be appropriate for a hospital population, and ignore other data that can add to the precision and granularity of the risk estimate. The introduction of electronic recording of vital signs and electronic patient records means that the parsimony and simplicity required by paper-based systems are now less relevant.

Main findings and strengths

This was a proof-of-concept study for developing and validating an enhanced EWS

For peer review only - http://bmjopen.bmj.com/site/about/guidelines.xhtml

that uses many more electronically held variables than the conventional vital signs, and which combined dynamic and static methods of risk evaluation, as typically used in other prediction disciplines such as meteorology (35) and imaging (36). We chose to study post-ICU patients because they have a detailed electronic record generated during their ICU stay, a high adverse event rate after ICU discharge, and because a significant body of literature exists on variables associated with adverse outcomes. However, the design principles we used in this study can be applied to any acute care patient group where sufficient data are available electronically.

As part of this work we developed a new scoring system for predicting in-hospital mortality and re-admission to ICU from data collected during a patient's ICU stay. A systematic review in 2013 (37) identified seven different published scoring systems that quantify the risk of mortality or re-admission at varying intervals after ICU discharge. However, only two studies verified their systems with an external (independent) validation dataset (38, 39). They obtained AUROC values similar to those found in this study.

RSI is a dynamic system that computes an updated risk estimate every time a new vital sign is recorded. It combines this dynamic risk estimate derived from the routinely-measured vital signs on the acute ward (RS_2) with a "static" risk estimate that is computed immediately after discharge from ICU using data from the ICU CIS (RS_1). As the performance of RS_1 is expected to worsen as the time from discharge from ICU increases because patients' conditions change (see Figure SM3-3), we used a time-dependent function to reduce its contribution over time, with the patient's risk score becoming increasingly determined by vital-signs based RS_2. The combined risk estimates as given by RSI improved the predictive power of our scoring system when compared with RS_2.

In this study, a compound outcome of in-hospital mortality and ICU re-admission was used as opposed to only using in-hospital mortality (20). Patients discharged from ICU with curative intent may die in hospital in spite of full, timely and appropriate care. They may also die because they do not receive the care they need in a timely fashion. Developing a model using in-hospital mortality as the sole outcome would limit the power of such a model, as appropriately-treated survivors, with a re-admission to ICU, would not contribute to the adverse events. Hence, there would be a risk that the resulting model would predict inevitable, rather than preventable, deaths, as also noted by others (40, 41). Our main goal was to develop a system that would identify patients who were likely to respond to earlier intervention of higher acuity care. Whilst ICU re-admission does not capture all the appropriately treated survivors, there is no other marker readily available in hospital electronic records to identify these patients. A similar compound outcome has been used in validation studies of EWSs for the same reason (21, 31, 42). We further note that RSI was considerably better at predicting in-hospital mortality than ICU re-admission (see Table 3). This has also been noted in other studies reporting evaluation of EWS

For peer review only - http://bmjopen.bmj.com/site/about/guidelines.xhtml

systems (21, 42, 43), where ICU admissions are less reliably identified than in-hospital death.

Limitations

This work has a number of limitations. We studied a very specific group of patients who were at high risk of in-hospital deterioration and who had detailed records of their ICU stay before they were discharged to the ward. We could only study the variables available in the ICU electronic records; hence, some of the candidate variables identified in the systematic review could not be included in the model. In addition, as vital-sign data had to be prospectively collected from consenting patients and transcribed from paper charts, the number of patients in the study, and therefore the number of adverse events, was limited.

We assessed the performance of the combined scoring system developed using the methodology commonly used to assess that of EWS systems. This uses AUROC measures based on paired vital-sign recordings and events within fixed time periods; i.e., derived outcome of occurrence of an adverse event within (e.g.) 24h of a vital-sign measurement. Therefore, AUROC values represent the probability that any randomly-chosen observation followed within the chosen time period by in-hospital death or ICU re-admission has a higher risk score than any randomly-chosen observation not followed by an event in the same time period (44). That is, repeated measurements from the same patient were used for evaluating the performance of scoring systems, which assumes that the scores computed from each observation set for that patient are independent (which is the usual approach when evaluating EWS systems). However, this assumption may not hold in practice; i.e., a vital-sign measurement at one point in time is likely to be correlated with previous measurements. This, alongside the highly imbalanced dataset (in which the outcome occurs infrequently), gives rise to AUROCs with high values that are not truly comparable with AUROCs from single predictor/single outcome algorithms where the unit of analysis is a patient-admission (45).

An external (independent) dataset was used to validate the risk scoring systems. RSI did not perform as well as expected for the combined outcome. This was primarily because the combined system over-estimated the risk of adverse events in higher-risk patients, possibly reflecting a difference in the patient population at the two hospital sites (46). The ICUs at the OUH admit tertiary referral patients not seen in RBH; hence, risk associated with these patients captured in the scoring system built with the development dataset may not have added explanatory power for patient admissions in the validation dataset.

Finally, we note that the performance of our scoring system for predicting adverse events exceeds that of previously published EWS algorithms. This should, however, be interpreted with caution, as most of these systems were developed and validated

For peer review only

on all hospital admissions to acute care areas, and our system was developed on a very specific population.

Conclusions

Scoring systems, such as EWSs, are used to identify hospitalised patients at risk of adverse events. In this study, we developed a bipartite score based on machine learning that encompasses the patient state at the time of ICU discharge, as well as vital signs recorded on the wards at the time the risk score is calculated. We showed that a scoring system incorporating data from a patient's stay in an ICU has better performance than typically-used EWS systems based on vital signs alone.

For peer review only

Ethical Approval and Consent to participate

The project had UK Health Research Authority Confidentiality Advisory Group approval (ref: ECC 7-05(f)/2011), National Research Ethics Service Committee approval (ref: 11/SC/0440) for retrospective data collection and analysis, and National Research Ethics Service Committee approval (ref: 12/SC/0357) for prospective data collection and analysis.

Consent for publication

Not applicable.

Availability of data and materials

Fully anonymised individual participant data may be made available to researchers directly affiliated to an academic institution or charitable organisation who provide an independently-reviewed, methodologically sound proposal. The data will be available for a period starting six months after publication and ending one year later. Only data required to answer the primary question posed by the proposed research will be made available. Researchers should contact Professor Young in the first instance. Other data and outputs from the project (Health Innovation Challenge Fund ref: HICF-0510-006) may be available on request.

Competing interests

JDY, PJW, and LT report grants from Wellcome Trust/UK Department of Health. PJW and LT report grants from National Institute for Health Research, personal fees from Sensyne Health, outside the submitted work.

Funding

This study was supported by the Department of Health and Wellcome Trust through the Health Innovation Challenge Fund (ref: HICF-0510-006). The views expressed in this publication are those of the authors and not necessarily those of the Department of Health & Social Care or Wellcome Trust. PJW, DAC and LT are supported by the NIHR Biomedical Research Centre Oxford. MAFP is supported by a Drayson Research Fellowship.

For peer review only

Author contributions

David A Clifton: Substantial contributions to the conception and design of the work, and analysis and interpretation of data for the work. Revising this manuscript critically for important intellectual content. Final approval of the manuscript. Agreement to be accountable for all aspects of the work.

Julie L Darbyshire: Substantial contributions to the design of the work. Revising this manuscript critically for important intellectual content. Final approval of the manuscript. Agreement to be accountable for all aspects of the work.

Alistair Johnson: Substantial contributions to the acquisition and analysis of data for the work. Revising this manuscript critically for important intellectual content. Final approval of the manuscript. Agreement to be accountable for all aspects of the work.

Marco A F Pimentel: Substantial contributions to the acquisition, analysis and interpretation of data for the work. Revising this manuscript critically for important intellectual content. Final approval of the manuscript. Agreement to be accountable for all aspects of the work.

Ian Rechner: Substantial contributions to the acquisition of data for the work. Revising this manuscript critically for important intellectual content. Final approval of the manuscript. Agreement to be accountable for all aspects of the work.

Lionel Tarassenko: Substantial contributions to the conception and design of the work. Substantial contributions to the acquisition, analysis, and interpretation of data for the work. Drafting the work and revising it critically for important intellectual content. Final approval of the version to be published. Agreement to be accountable for all aspects of the work.

Andrew Walden: Substantial contributions to the acquisition of data for the work. Revising this manuscript critically for important intellectual content. Final approval of the manuscript. Agreement to be accountable for all aspects of the work.

Peter J Watkinson: Substantial contributions to the conception and design of the work. Substantial contributions to the acquisition, analysis, and interpretation of data for the work. Drafting the work and revising it critically for important intellectual content. Final approval of the version to be published. Agreement to be accountable for all aspects of the work.

J Duncan Young: Substantial contributions to the conception and design of the work. Substantial contributions to the acquisition, analysis, and interpretation of data for the work. Drafting the work and revising it critically for important intellectual content. Final approval of the version to be published. Agreement to be accountable for all aspects of the work.

For peer review only

Acknowledgements

Dr Jonathan Burgess, who sadly passed away before seeing the fruits of his labours, undertook much of the early work on the identification of candidate variables in this project.

For peer review only

References

1. Schein RMH, Hazday N, Pena M, et al.: Clinical Antecedents to In-Hospital Cardiopulmonary Arrest. *Chest* 1990; 98:1388–1392
2. McQuillan P, Pilkington S, Allan A, et al.: Confidential inquiry into quality of care before admission to intensive care. *BMJ* 1998; 316:1853–1858
3. Kause J, Smith G, Prytherch D, et al.: A comparison of Antecedents to Cardiac Arrests, Deaths and EMergency Intensive care Admissions in Australia and New Zealand, and the United Kingdom—the ACADEMIA study. *Resuscitation* 2004; 62:275–282
4. Churpek MM, Yuen TC, Huber MT, et al.: Predicting cardiac arrest on the wards: A nested case-control study. *Chest* 2012;
5. Berlot G, Pangher A, Petrucci L, et al.: Anticipating events of in-hospital cardiac arrest. *Eur J Emerg Med* 2004; 11:24–28
6. Morgan R, Williams F, Wright M: An early warning scoring system for detecting developing critical illness. *Clin Intensive Care* 1997; 8:100
7. Smith GB, Prytherch DR, Schmidt PE, et al.: Review and performance evaluation of aggregate weighted “track and trigger” systems. *Resuscitation* 2008;
8. Alam N, Hobbelink EL, van Tienhoven AJ, et al.: The impact of the use of the Early Warning Score (EWS) on patient outcomes: A systematic review. *Resuscitation* 2014; 85:587–594
9. National Institute for Health and Care Excellence (NICE): Acutely ill adults in hospital: recognising and responding to deterioration. *Clin Guidel* 2007;
10. Adam S, Odell M: An acute problem? A report of the National Confidential Enquiry into Patient Outcome and Death. *Nurs Crit Care* 2005; 10:225–227
11. National Institute for Health and Care Excellence (NICE): Sepsis: recognition, diagnosis and early management. *BJU Int* 2018; 121:497–514
12. Christofidis MJ, Hill A, Horswill MS, et al.: Observation charts with overlapping blood pressure and heart rate graphs do not yield the performance advantage that health professionals assume: an experimental study. *J Adv Nurs* 2014; 70:610–624
13. Elliott D, Allen E, Perry L, et al.: Clinical user experiences of observation and response charts: focus group findings of using a new format chart incorporating a track and trigger system. *BMJ Qual Saf* 2015; 24:65–75
14. Smith GB, Prytherch DR, Schmidt P, et al.: Hospital-wide physiological surveillance-A new approach to the early identification and management of the sick patient. *Resuscitation* 2006;
15. Wong D, Bonnici T, Knight J, et al.: SEND: a system for electronic notification and documentation of vital sign observations. *BMC Med Inform Decis Mak* 2015; 15:68
16. Bleyer AJ, Vidya S, Russell GB, et al.: Longitudinal analysis of one million vital

For peer review only

signs in patients in an academic medical center. *Resuscitation* 2011;

17. Green M, Lander H, Snyder A, et al.: Comparison of the Between the Flags calling criteria to the MEWS, NEWS and the electronic Cardiac Arrest Risk Triage (eCART) score for the identification of deteriorating ward patients. *Resuscitation* 2018; 123:86–91
18. Moons KGM, Altman DG, Reitsma JB, et al.: Transparent Reporting of a multivariable prediction model for Individual Prognosis Or Diagnosis (TRIPOD): Explanation and Elaboration. *Ann Intern Med* 2015; 162:W1
19. Intensive Care National Audit & Research Centre (ICNARC): About the CMP. *Accessed from https//www.icnarc.org/Our-Audit/Audits/Cmp/About; Accessed July 2020*
20. Prytherch DR, Smith GB, Schmidt PE, et al.: ViEWS-Towards a national early warning score for detecting adult inpatient deterioration. *Resuscitation* 2010; 81:932–937
21. Smith GB, Prytherch DR, Meredith P, et al.: The ability of the National Early Warning Score (NEWS) to discriminate patients at risk of early cardiac arrest, unanticipated intensive care unit admission, and death. *Resuscitation* 2013; 84:465–470
22. Pimentel MAF, Clifton DA, Clifton L, et al.: A review of novelty detection. *Signal Processing* 2014; 99:215–249
23. Johnson AEW, Burgess J, Pimentel MAF, et al.: Physiological trajectory of patients pre and post ICU discharge. In: 2014 36th Annual International Conference of the IEEE Engineering in Medicine and Biology Society. IEEE; 2014. p. 3160–3163.
24. Pimentel MAF, Clifton DA, Clifton L, et al.: Modelling physiological deterioration in post-operative patient vital-sign data. *Med Biol Eng Comput* 2013;
25. Lemeshow S, Hosmer DW: A review of goodness of fit statistics for use in the development of logistic regression models. *Am J Epidemiol* 1982;
26. Kramer AA, Zimmerman JE: Assessing the calibration of mortality benchmarks in critical care: The Hosmer-Lemeshow test revisited*. *Crit Care Med* 2007; 35:2052–2056
27. Cox DR: Two further applications of a model for binary regression. *Biometrika* 1958; 45:562–565
28. DiCiccio TJ, Efron B: Bootstrap confidence intervals. *Stat Sci* 1996;
29. Gardner-Thorpe J, Love N, Wrightson J, et al.: The value of Modified Early Warning Score (MEWS) in surgical in-patients: A prospective observational study. *Ann R Coll Surg Engl* 2006; 88:571–575
30. Paterson R, MacLeod DCD, Thetford D, et al.: Prediction of in-hospital mortality and length of stay using an early warning scoring system: clinical audit. *Clin Med J R Coll Physicians London* 2006; 6:281–284
31. Watkinson PJ, Pimentel MAF, Clifton DA, et al.: Manual centile-based early warning scores derived from statistical distributions of observational vital-sign data. *Resuscitation* 2018; 129:55–60

For peer review only

32. Akre M, Finkelstein M, Erickson M, et al.: Sensitivity of the Pediatric Early Warning Score to Identify Patient Deterioration. *Pediatrics* 2010; 125:e763–e769

33. Skaletzky SM, Raszynski A, Totapally BR: Validation of a Modified Pediatric Early Warning System Score. *Clin Pediatr (Phila)* 2012; 51:431–435

34. Hravnak M, Devita MA, Clontz A, et al.: Cardiorespiratory instability before and after implementing an integrated monitoring system. *Crit Care Med* 2011;

35. Gneiting T, Ranjan R: Combining predictive distributions. *Electron J Stat* 2013;

36. Akhondi-Asl A, Hoyte L, Lockhart ME, et al.: A logarithmic opinion pool based STAPLE algorithm for the fusion of segmentations with associated reliability weights. *IEEE Trans Med Imaging* 2014;

37. Hosein FS, Bobrovitz N, Berthelot S, et al.: A systematic review of tools for predicting severe adverse events following patient discharge from intensive care units. *Crit Care* 2013;

38. Daly K: Reduction in mortality after inappropriate early discharge from intensive care unit: logistic regression triage. *BMJ* 2001; 322:1274–1274

39. Fernandez R, Serrano JM, Umaran I, et al.: Ward mortality after ICU discharge: A multicenter validation of the Sabadell score. *Intensive Care Med* 2010;

40. Tarassenko L, Clifton DA, Pinsky MR, et al.: Centile-based early warning scores derived from statistical distributions of vital signs. *Resuscitation* 2011; 82:1013–1018

41. Churpek MM, Yuen TC, Edelson DP: Predicting clinical deterioration in the hospital: The impact of outcome selection. *Resuscitation* 2013; 84:564–568

42. Redfern OC, Pimentel MAF, Prytherch D, et al.: Predicting in-hospital mortality and unanticipated admissions to the intensive care unit using routinely collected blood tests and vital signs: Development and validation of a multivariable model. *Resuscitation* 2018; 133:75–81

43. Subbe CP: Validation of a modified Early Warning Score in medical admissions. *QJM* 2001; 94:521–526

44. Jarvis SW, Kovacs C, Briggs J, et al.: Are observation selection methods important when comparing early warning score performance? *Resuscitation* 2015; 90:1–6

45. Romero-Brufau S, Huddleston JM, Escobar GJ, et al.: Why the C-statistic is not informative to evaluate early warning scores and what metrics to use. *Crit Care* 2015; 19:285

46. Subbaswamy A, Schulam P, Saria S: Preventing Failures Due to Dataset Shift: Learning Predictive Models That Transport. *AISTATS 2019 - 22nd Int Conf Artif Intell Stat* 2018;

For peer review only

Additional files

Additional file 1

File name: Additional file 1

File format: .docx

Title: Model development

Description: Detailed description of the methods used to develop the models.

Additional file 2

File name: Additional file 2

File format: .docx

Title: Comparative scoring systems

Description: Description of the components, weightings and details of other scoring systems implemented in this study.

Additional file 3

File name: Additional file 3

File format: .docx

Title: Additional results

Description: Further results of the performance of the models.

For peer review only

Tables Legends

Table 1. Details of the ICU admissions and the subgroups of post-ICU admissions included in the development and validation cohorts. Continuous (numeric) variables are displayed as *median [interquartile range] (mean)*, and count variables are displayed as *counts (%)*. "OUH" refers to admissions from the Oxford University Hospitals NHS Foundation Trust's two adult ICUs. "RBH" refers to admissions from the Royal Berkshire NHS Foundation Trust's single adult ICU. "LoS" denotes length of stay.

Table 2. Performance of the first model (RS_1) on both development and validation datasets for the combined outcome (or adverse event) of in-hospital death or re-admission to ICU at any point after discharge from ICU. Performance for each event separately are also displayed. Cox's calibration regression: for a good calibration, α should be close to 0, and β should be close to 1. AUROC denotes area under the receiver operating characteristic curve, shown with mean (standard deviation, SD).

Table 3. Area under the receiver operating characteristic curve (AUROC), standard deviation and corresponding 95% confidence interval (CI) for the developed risk scoring index (RSI) and other competitive early warning score (EWS) systems, using adverse event (re-admission to ICU or in-hospital death) within 24h of an observation set as the (compound) outcome. Results are shown for each outcome independently using both development and validation datasets.

Figure Legends

Figure 1. Representation of a set of the variables acquired for an example patient included in our study pre (represented with circles) and post discharge from the ICU. Variables include vital signs (dark grey), laboratory tests (lighter grey) and interventions/treatments (white) performed. The grey vertical line marks the patient's ICU discharge timepoint. A more detailed electronic patient record is "generated" during the patient's ICU stay. We also note the different frequency of measurement of the vital signs in the ICU from that on the ward.

Figure 2. Performance of the developed risk scoring system (RSI) and the other competitive scoring systems considered for predicting an adverse event (either re-admission to ICU or in-hospital death) within 12, 24, 36, 48, and 72h of a vital-sign

For peer review only

observation set. The left-hand panel shows the performance on the development dataset, the right-hand panel shows the performance on the validation dataset. The performance is represented with the mean AUROC (area under the receiver operating characteristic curve) values.

For peer review only

Table 1.

	Development (OUH)	Validation (RBH)
**ICU admissions included, n**	**8,162**	**3,421**
Age, years	62 [47 – 72] (59)	65 [51 – 75] (62)
Females sex, n	3,381 (41.4%)	1,480 (43.3%)
LoS in ICU, days	1.9 [1.0 – 4.0] (4.2)	2.0 [1.0 – 4.5] (4.2)
Outcome:		
ICU re-admission, n	543 (6.7%)	154 (4.5%)
Time to ICU re-admission, hours	92 [37 – 205] (225)	101 [43 – 283] (340)
In-hospital death, n	462 (5.7%)	218 (6.4%)
Time to in-hospital death, hours	296 [84 – 781] (701)	204 [66 – 600] (540)
Adverse event, n	902 (11.1%)	329 (9.6%)
**Post-ICU admissions included, n**	**273**	**136**
Age, year	64 [51 – 72] (60)	66 [55 – 73] (63)
Females sex, n	85 (31.1)	61 (45)
LoS in ICU, days	1.9 [1.0 – 3.9] (3.5)	1.3 [0.9 – 3.0] (3.4)
Outcome:		
ICU re-admission, n	18 (6.6%)	9 (6.6%)
Time to ICU re-admission, hours	162 [73 – 220] (215)	108 [72 – 202] (153)
In-hospital death, n	6 (2.2%)	4 (2.9%)
Time to in-hospital death, hours	216 [66 – 514] (356)	322 [244 – 365] (288)
Adverse event, n	23 (8.4%)	11 (8.1%)

For peer review only - http://bmjopen.bmj.com/site/about/guidelines.xhtml

Table 2.

	Development dataset		
	**Adverse event**	**In-hospital death**	**ICU re-admission**
Hosmer-Lemeshow statistic	50.81	339.07	224.94
Brier score	0.087	0.050	0.065
Cox's calibration:			
α	-0.009	-0.037	0.013
β	0.988	1.104	0.472
AUROC (SD)	0.782 (0.022)	0.843 (0.022)	0.716 (0.022)
	**Validation dataset**		
Hosmer-Lemeshow statistic	57.35	175.68	275.08
Brier score	0.081	0.058	0.058
Cox's calibration:			
α	0.017	-0.012	0.033
β	0.627	0.475	0.268
AUROC (SD)	0.723 (0.014)	0.780 (0.017)	0.623 (0.019)

For peer review only

Table 3.

	Adverse event	In-hospital death	ICU re-admission
	**Development dataset**		
MEWS	0.749 (0.022) [0.734 to 0.764]	0.752 (0.055) [0.717 to 0.789]	0.747 (0.021) [0.733 to 0.761]
SEWS	0.754 (0.019) [0.741 to 0.765]	0.785 (0.050) [0.753 to 0.819]	0.744 (0.022) [0.730 to 0.759]
NEWS	0.757 (0.020) [0.742 to 0.771]	0.889 (0.030) [0.867 to 0.912]	0.737 (0.022) [0.721 to 0.753]
CEWS	0.761 (0.021) [0.748 to 0.775]	0.887 (0.029) [0.866 to 0.908]	0.740 (0.022) [0.727 to 0.754]
RS_2	0.767 (0.019) [0.755 to 0.780]	0.859 (0.041) [0.836 to 0.889]	0.745 (0.020) [0.732 to 0.758]
**RSI**	**0.782 (0.018) [0.770 to 0.794]**	**0.931 (0.007) [0.926 to 0.936]**	**0.753 (0.017) [0.743 to 0.764]**
	**Validation dataset**		
MEWS	0.632 (0.032) [0.610 to 0.655]	0.697 (0.054) [0.661 to 0.733]	0.632 (0.033) [0.610 to 0.655]
SEWS	0.678 (0.037) [0.653 to 0.703]	0.678 (0.057) [0.642 to 0.717]	0.681 (0.038) [0.657 to 0.706]
NEWS	0.653 (0.044) [0.621 to 0.683]	0.675 (0.063) [0.630 to 0.719]	0.649 (0.043) [0.622 to 0.679]
CEWS	0.672 (0.036) [0.648 to 0.695]	0.685 (0.061) [0.643 to 0.728]	0.670 (0.036) [0.645 to 0.596]
RS_2	0.693 (0.040) [0.669 to 0.718]	0.681 (0.059) [0.644 to 0.722]	0.693 (0.041) [0.666 to 0.721]
**RSI**	**0.724 (0.027) [0.704 to 0.741]**	**0.725 (0.045) [0.694 to 0.757]**	**0.722 (0.028) [0.702 to 0.741]**

For peer review only

Figure 1.

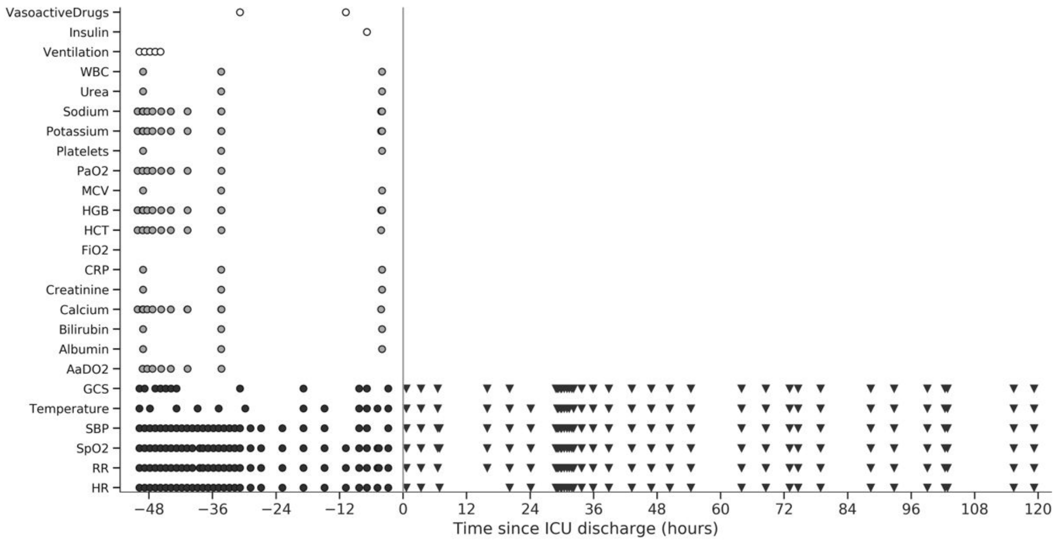

For peer review only

Figure 2.

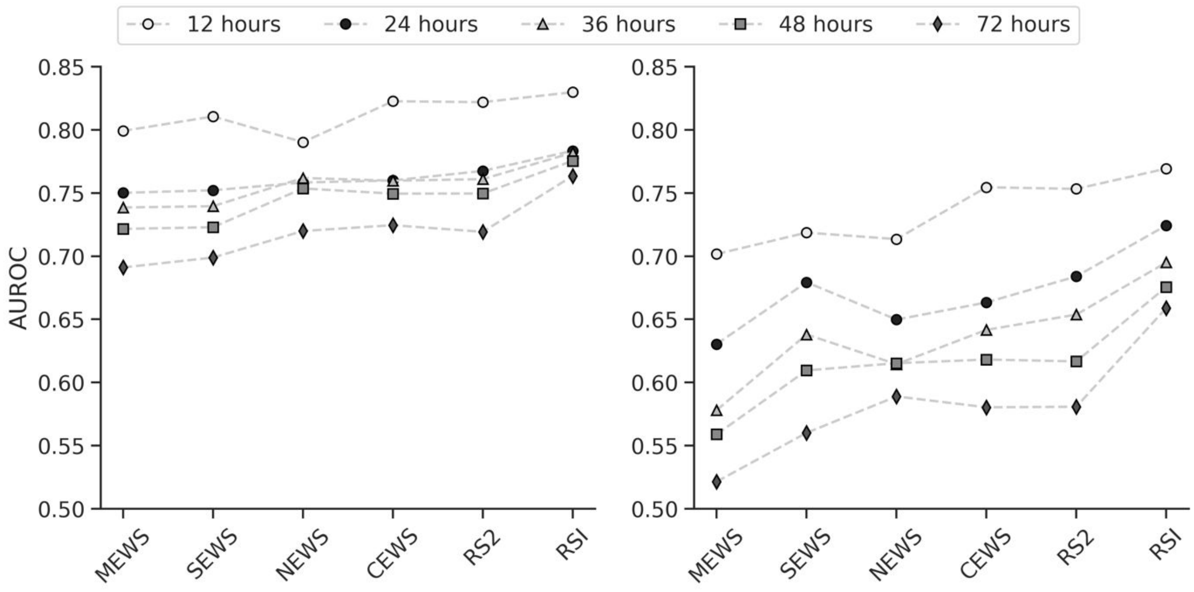

peer review only

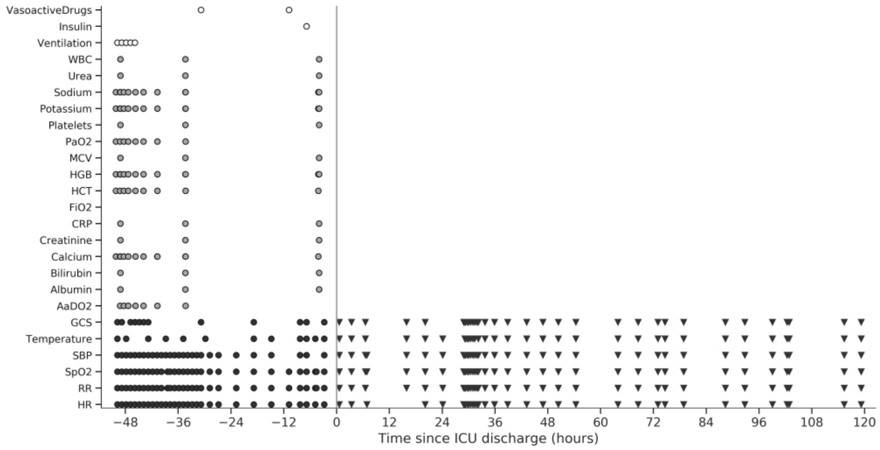

Figure 1: Representation of a set of the variables acquired for an example patient included in our study pre (represented with circles) and post discharge from the ICU. Variables include vital signs (dark grey), laboratory tests (lighter grey) and interventions/treatments (white) performed. The grey vertical line marks the patient's ICU discharge timepoint. A more detailed electronic patient record is "generated" during the patient's ICU stay. We also note the different frequency of measurement of the vital signs in the ICU from that on the ward.

966x515mm (138 x 138 DPI)

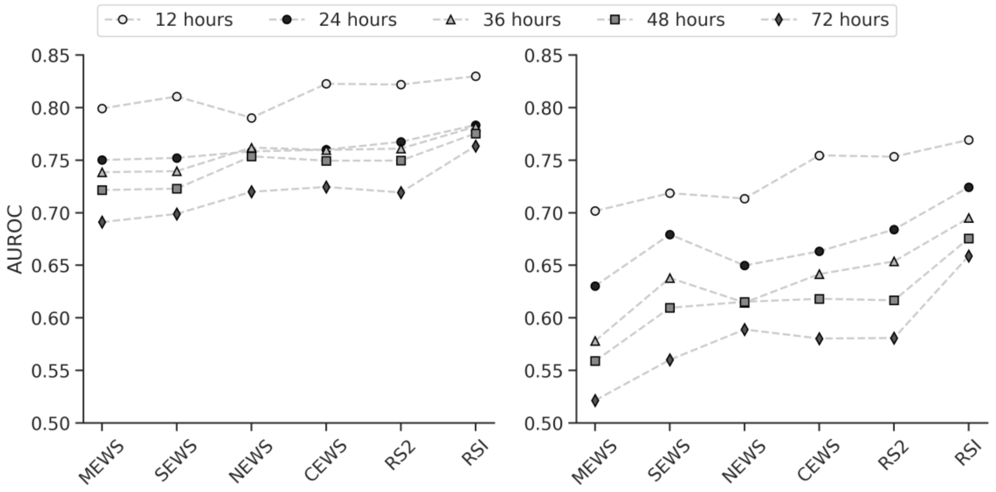

Figure 2: Performance of the developed risk scoring system (RSI) and the other competitive scoring systems considered for predicting an adverse event (either re-admission to ICU or in-hospital death) within 12, 24, 36, 48, and 72h of a vital-sign observation set. The left-hand panel shows the performance on the development dataset, the right-hand panel shows the performance on the validation dataset. The performance is represented with the mean AUROC (area under the receiver operating characteristic curve) values

662x331mm (138 x 138 DPI)

Additional file 1 – Model development

We describe in more detail the methods used to develop the model in this section.

Predictors

Our proposed approach uses a conceptual model in which we estimate the risk of a patient experiencing an adverse event immediately after discharge from ICU. This risk score (RS_1) is estimated from variables recorded during their stay in the ICU. After discharge from ICU, the patient's risk of experiencing an adverse event within the next 24 hours is updated in a time-varying manner (RS_2), using those measurements of the vital signs that are routinely measured during subsequent ward care.

ICU-based feature representation:

To estimate the risk of a patient experiencing an adverse event immediately after discharge from ICU, we considered candidate variables that were available electronically in our databases. The final list of candidate variables included the following physiological variables:

- Heart rate (HR), measured in beats per minute
- Respiratory rate (RR), in breaths per minute
- Systolic (SBP), diastolic and mean blood pressure, in mmHg
- Temperature, in degrees Celsius
- Peripheral oxygen saturation (SpO_2), in %
- Level of consciousness, using the Glasgow Coma Scale (GCS)

The following laboratory test results were included in the list:

- Alveolar-arterial oxygen partial pressure gradient ($AaDO_2$), in kPa
- Albumin, in g/L
- Bilirubin (total), in μmol/L
- Calcium (adjusted), in mmol/L
- Creatinine, in mmol/L
- C-reactive protein (CRP), in mg/L
- Haematocrit (HCT), in %
- Haemoglobin (HGB), in g/dL
- Lactate, in mmol/L
- Mean corpuscular volume (MCV), in fL
- Ratio of partial pressure of oxygen : fraction of inspired oxygen (PaO_2/FiO_2), in kPa
- pH
- Platelet count, in 10^9 cells/L

For peer review only

- Potassium, in mmol/L
- Sodium, in mmol/L
- Urea, measured in mmol/L
- Urine output, in mL
- White blood cell count (WBC), in 10^9 cells/L

The list also included demographic and administrative-based variables, such as age at admission, gender, number of hours between hospital admission and ICU admission, and the Index of Multiple Deprivation score (1), derived for patients with a valid postcode. We included variables related to procedures and treatments, such as the number of vasoactive drugs administered, total fluid balance, administration of insulin, enteral and parenteral nutrition feeding, and the use of mechanical ventilation, tracheostomy and central venous catheters.

To determine the risk of future compound outcome after discharge from the ICU, we derived several features from all candidate variables acquired during the patients' stay in the ICU. These features are based on the extremes of the variables considered. We generated maximum, minimum and variability (as given by the standard deviation) features for the physiological variables and laboratory test results from different periods of the ICU stay (including the first 24 hours of the patient in the ICU, their last 72, 48, and 24 hours, and/or their entire ICU stay). Additional dichotomous variables were generated if the values were in the upper or lower 5th percentile of the observed corresponding ranges, in order to account for potential non-linear associations of the variables with the adverse outcomes. Procedure and treatment variables were converted to dichotomous features for indicating whether a given procedure was performed or not, or whether a given medication was administered or not, over the entire ICU stay or in the last 24 hours of the ICU stay. This procedure resulted in 161 candidate features (including features from demographic information) for building a prediction model.

Post-ICU feature representation:

All vital-sign observations performed after discharge from ICU, as part of routine patient monitoring on acute care wards and collected prospectively for this study, were considered for analysis. Each set of vital signs include heart rate, systolic blood pressure, respiratory rate, body temperature, neurological status assessment using the Alert-Verbal-Painful-Unresponsive (AVPU) scale, peripheral oxygen saturation from pulse oximetry (SpO_2), a record of whether the patient was receiving supplemental oxygen at the time of the SpO_2 measurement, and the date and time of the observation. Vital-sign measurements are typically recorded every 4 or 6 hours throughout the patient's stay on the ward. At each measurement timestamp, in case of an incomplete vital-sign observation set, we used the most recent value of each variable (i.e., by carrying forward the last measurement).

For peer review only

Pre-processing and missing data

We identified obvious deviations from expected distributions and ranges of the data features using frequency graphs for all numerical and dichotomous features. Possible physiological ranges for the numerical features were defined according to clinical review and expert panel expertise, and values outside these ranges were not included in the analyses. Missing values were imputed with median and mode values from the feature distributions of numerical and dichotomous features, respectively. While other methods were considered, such as multiple imputation, the use of the median and mode was simpler and was deemed sufficient for this work in which the amount of missing data was low.

All numerical features were then standardised using a zero-mean, unit-variance transformation (i.e., using the mean and standard deviations from the feature distributions). This prevents features with relatively small changes in their units of measurement (such as temperature) from being dominated by features with relatively large changes (such as blood pressure), thus ensuring that all features have similar dynamic ranges.

For both imputation and normalisation, the parameters' values (for the median, mode, mean and standard deviation) found for the development dataset were used.

ICU-based model (RS_1)

We used all candidate features derived from the variables acquired during the patients' stay in the ICU to build the first model. A L1-regularised logistic regression using the "glmnet" package in Matlab (Mathworks, Natick, Massachusetts, USA) for predicting the compound outcome (in-hospital death or re-admission to ICU). L1-regularisation shrinks the less important features' coefficients to zero thus effectively removing those features that are deemed to be uninformative to the outcome variable. We estimated the regularisation parameter using LASSO (Least Absolute Shrinkage and Selection Operator) regression in a 5-fold cross-validation on the development set.

Vital-sign based model (RS_2)

For the second scoring system, RS_2, we used the vital signs (heart rate, respiratory rate, systolic blood pressure, oxygen saturation, and temperature) recorded in hospital after discharge from the ICU. For this model, rather than a *supervised learning approach*, we considered a novelty detection method (or one-class classification method), which does not require an outcome variable for development (2). This is useful where event rates are extremely low, or total sample sizes are constrained. Similar approaches have been used in our previous research (3–6) and underpin a commercial, clinically-used system, described in (7).

For peer review only

We considered the construction of a multivariate, (possibly) multimodal model of normality, based on the vital-sign observation sets recorded during the 24 hours that preceded discharge home. If the patient had not died, was not re-admitted to the ICU and was not discharged by the 14th post-ICU day, we used recorded data on that day. These observation sets are thus assumed to contain the vital-sign values from the most stable period of the patient's hospital stay, because these data were acquired when patients are at their most "stable" after discharge from ICU. This set of "normal" data contains $N = 1{,}082$ 5-dimensional vital-sign vectors, $\mathbf{X} = \{\mathbf{x_1},\ldots,\mathbf{x}_N\} \in \mathbb{R}^5$, which were subsequently used for the construction of our model of normality.

A kernel density estimate, or KDE (2) was used to estimate the probability density function (pdf) of the set of five vital signs. This is in a non-parametric technique where no *a priori* assumptions about the form of the underlying probability distribution are made. Our notation follows that reported previously (5, 6). The data pdf $p(\mathbf{x})$ was estimated using the $N = 1{,}082$ set of observations as shown in (SM1-1).

$$p(\mathbf{x}|\mathbf{x}_i,\sigma) = \frac{1}{N(2\pi)^{D/2}\sigma^D}\sum_{i=1}^{N}\exp\left(\frac{-|\mathbf{x}-\mathbf{x}_i|^2}{2\sigma^2}\right) \quad \text{(SM1-1)}$$

This is a weighted sum of Gaussian kernels, each with identical variance σ^2 (i.e., isotropic kernels), centred on the observation sets $\mathbf{x_1},\ldots,\mathbf{x}_N$.

The nearest-neighbour method was used to estimate the variance. Briefly, this method involves determining the squared Euclidean distance (Δ) for each observation i to its 10 nearest neighbours (*NNs*), as shown in (SM1-2).

$$\Delta_i = \frac{1}{10}\sum_{j\in\{NNs\}}\|\mathbf{x}_i - \mathbf{x}_j\| \quad \text{(SM1-2)}$$

This quantity, Δ, is then used to estimate the variance σ^2, as shown in (SM1-3).

$$\sigma = \frac{1}{N}\sum_{i=1}^{N}\Delta_i \quad \text{(SM1-3)}$$

Estimation of the underlying pdf of normal vital-sign data provides a means of quantifying the degree to which a given set of observations is abnormal. The likelihood $p(\mathbf{x}|\mathbf{x}_i,\sigma)$, a measure which represents the probability of observing a set of measurements given a pdf, can be used for this purpose. Thus, we define the novelty score as in (SM1-4).

$$z(\mathbf{x}) = -\log p(\mathbf{x}|\mathbf{x}_i,\sigma) \quad \text{(SM1-4)}$$

For normal data, the new observation $\mathbf{x}$ will be similar to previously-seen normal observations $\mathbf{x}_i$, and so the likelihood will be high. Consequently, the negative log-likelihood will be low, and so the novelty score $z(\mathbf{x})$ will be low. Conversely, for abnormal data, the data will be dissimilar and the likelihood will be low, and consequently the novelty score $z(\mathbf{x})$ will be high. This procedure ensures that the novelty score can be interpreted as most early warning scores (EWS), in that high scores are associated with higher abnormality of the vital signs.

For peer review only

In short, this model allows the generation of a score corresponding to an assessment of whether a vital-sign set should be deemed "stable" with respect to the development dataset (i.e., the observation sets used for training the model). By extension, it further allows the model to estimate the degree of abnormality of a given vital-sign observation set.

Risk Score Index (RSI)

An overall risk score, the risk score index (RSI), was subsequently determined using a simple time-dependent linear combination of the two constituent risk scores, such that:

$$\mathrm{RSI} = \beta\left[\left(1 - \frac{t}{Tmax}\right)\mathrm{RS}_1\right] + \left[\left(\frac{t}{Tmax}\right)\mathrm{RS}_2\right] \qquad \text{(Equation 1)}$$

where t corresponds to the elapsed time (in hours) since the patient was discharged from ICU and has a maximum value of $Tmax$ hours. The time-dependent weighting function allows the contribution of RS_1 to gradually decrease with time after discharge from the ICU. The patient's risk of future adverse events becomes increasingly determined by the values of the vital-sign measurements taken on the acute ward (i.e., RS_2). The parameter β is used to adjust the weight of RS_1 with respect to the time since discharge from ICU, and the parameter $Tmax$ corresponds to the time at which RS_1 no longer affects RSI; i.e., when $t \geq Tmax$, then $\mathrm{RSI} = RS_2$. The parameters β and $Tmax$ were determined using a grid-search algorithm with 3-fold cross-validation, by defining a grid of possible values of $\beta = [0.1,\ 0.2,\ 0.3, \ldots, 200]$ and $Tmax = [12, 24, 36, \ldots, 336]$. We selected the values of β and $Tmax$ that corresponded to the highest mean area under the curve of the receiver operating characteristic (AUROC) value across all cross-validation folds, using the compound outcome of in-hospital death or ICU re-admission within the next 24 hours of a vital-sign observation.

During the development of RSI, values of $\beta = 100.2$ and $Tmax = 96$ hours, for the linear time-dependent weighting function (see Equation 1), were obtained and used to calculate the risk scoring index.

Model validation and statistical analysis

To validate the first model, RS_1, its discrimination and calibration were analysed. Discrimination is defined as the ability of the model to separate non-event patients from patients who had an adverse event after ICU discharge, and was assessed using the AUROC metric. Calibration assesses the degree of correspondence between the estimated probability of occurrence of an adverse event and that

For peer review only

actually observed. This was tested using a goodness of fit test, the Hosmer-Lemeshow "C" statistic (8). When the predicted probability of adverse events of the prognostic model differs significantly from the observed pattern, the calibration ability of the model is deemed to be poor. As the Hosmer-Lemeshow test does not measure the magnitude of miscalibration and is sensitive to sample size (9), calibration was also assessed with the Brier score and Cox's calibration regression. The latter assesses the degree of miscalibration by fitting a logistic regression of observed survival to the predicted log-odds of survival from the risk model (10). The performance of the first model was examined both for the compound outcome and each adverse event (in-hospital death and ICU re-admission) individually. The ability of the first model to predict future adverse events at increasing intervals from ICU discharge was also examined by calculating the AUROC for future events by day after discharge (up to 120 days).

The final model, RSI, was validated using the AUROC for the derived outcome of in-hospital death or ICU re-admission within the next *N* hours of a vital-sign observation/measurement performed after ICU discharge, in line with previous studies for evaluating EWS systems (11, 12). We evaluated the model for different values of *N*, with $N = [12, 24, 36, 48, 72]$ hours. We note that in this case the AUROC represents how well the scoring system RSI discriminates between observation sets followed by an adverse event and those with no subsequent adverse outcome within the next *N* hours. Therefore, the unit of analysis is a vital-sign set rather than a patient-admission, as performed for the validation of the first model.

We also considered each individual adverse event separately. To further understand the feasibility of implementation of the risk scoring systems in this setting, the burden of observation sets "triggered" for every correctly identified observation followed by an adverse event within 24 hours by the risk scoring system was also evaluated.

We report the cross-validation results using the development dataset. This gives an estimate of how our models perform on a random set of samples from the OUH Trust that were not used for developing the model. We also report the external validation results using data from the RBH Trust. Confidence intervals were estimated using bootstrap confidence intervals via percentiles with 500 samples (13).

For peer review only

References

1. GOV.UK (National Statistics): English indices of deprivation 2015. *Accessed from https//www.gov.uk/government/statistics/english-indices-of-deprivation-2015; Accessed January 2018*
2. Pimentel MAF, Clifton DA, Clifton L, et al.: A review of novelty detection. *Signal Processing* 2014; 99:215–249
3. Watkinson PJ, Barber VS, Price JD, et al.: A randomised controlled trial of the effect of continuous electronic physiological monitoring on the adverse event

rate in high risk medical and surgical patients. *Anaesthesia* 2006;

4. Clifton DA, Wong D, Clifton L, et al.: A large-scale clinical validation of an integrated monitoring system in the Emergency Department. *IEEE J Biomed Heal Informatics* 2013;

5. Johnson AEW, Burgess J, Pimentel MAF, et al.: Physiological trajectory of patients pre and post ICU discharge. In: 2014 36th Annual International Conference of the IEEE Engineering in Medicine and Biology Society. IEEE; 2014. p. 3160–3163.

6. Pimentel MAF, Clifton DA, Clifton L, et al.: Modelling physiological deterioration in post-operative patient vital-sign data. *Med Biol Eng Comput* 2013;

7. Orphanidou C, Clifton D, Shahab Khan, et al.: Telemetry-based vital sign monitoring for ambulatory hospital patients. In: 2009 Annual International Conference of the IEEE Engineering in Medicine and Biology Society. IEEE; 2009. p. 4650–4653.

8. Lemeshow S, Hosmer DW: A review of goodness of fit statistics for use in the development of logistic regression models. *Am J Epidemiol* 1982;

9. Kramer AA, Zimmerman JE: Assessing the calibration of mortality benchmarks in critical care: The Hosmer-Lemeshow test revisited*. *Crit Care Med* 2007; 35:2052–2056

10. Cox DR: Two further applications of a model for binary regression. *Biometrika* 1958; 45:562–565

11. Prytherch DR, Smith GB, Schmidt PE, et al.: ViEWS-Towards a national early warning score for detecting adult inpatient deterioration. *Resuscitation* 2010; 81:932–937

12. Badriyah T, Briggs JS, Meredith P, et al.: Decision-tree early warning score (DTEWS) validates the design of the National Early Warning Score (NEWS). *Resuscitation* 2014; 85:418–423

13. DiCiccio TJ, Efron B: Bootstrap confidence intervals. *Stat Sci* 1996;

For peer review only

Additional file 2 – Comparative scoring systems

In this section, we described the details of other scoring systems used in this study and how they were implemented using the information included in datasets. We also detail the components and weightings of the individual scores, as they were implemented in this work.

The early warning score (EWS) systems evaluated in this study include the modified EWS, or MEWS (1), the standardised EWS, or SEWS (2), the National EWS, or NEWS (3, 4) and the centile-based EWS, or CEWS (5). Their components and weightings are shown in tables SM2-1, SM2-2, SM2-3 and SM2-4, respectively. The core set of variables that are used in these scoring systems comprises heart rate (measured in beats per minute), respiratory rate (in breaths per minute), systolic and diastolic blood pressure (in mmHg), body temperature (in °C), oxygen saturation (denoted "SpO_2", in %), an assessment of neurological status (using the Alert-Verbal-Painful-Unresponsive or AVPU scale), a flag for whether the patient was receiving supplementary oxygen at the time of the SpO_2 measurement (denoted "Inspired O_2"), and the age to the patient at admission to hospital. At each observation set (or measurement timestamp), in case of an incomplete vital-sign observation set, we used the most recent value of each variable. Remaining missing data were dealt with as with the proposed scoring system (see section 2 in the main manuscript).

Table SM2-1. Modified Early Warning Score (see main text for units of the variables included).

Table SM2-2. Standardised Early Warning Score (see main text for units of the variables included).

Table SM2-3. National Early Warning Score (see main text for units of the variables included).

Table SM2-4. Centile-based Early Warning Score (see main text for units of the variables included).

For peer review only

Table SM2-1.

Modified Early Warning Score (MEWS)

	Score						
Variable	3	2	1	0	1	2	3
Heart rate	≤ 39	40 – 50	51 – 59	60 – 100	101 – 110	111 – 129	≥ 130
Resp. rate		≤ 8		9 – 14	15 – 20	21 – 29	≥ 30
Temperature		≤ 35.0		35.1 – 38.0		38.1 – 39.5	≥ 39.6
Systolic BP	≤ 69	70 – 80	81 – 100	101 – 149	150 – 169	170 – 179	≥ 180
AVPU scale				A	V	P	U

Table SM2-2.

Standardised Early Warning Score (SEWS)

	Score						
Variable	3	2	1	0	1	2	3
Heart rate	≤ 29	30 – 39	40 – 49	50 – 99	100 – 109	110 – 129	≥ 130
Resp. rate	≤ 8			9 – 20	21 – 30	31 – 35	≥ 36
Temperature	≤ 33.9	34.0 – 34.9	35.0 – 35.9	36.0 – 37.9	38.0 – 38.9	≥ 39.0	
Systolic BP	≤ 69	70 – 79	80 – 99	100 – 199		≥ 200	
SpO_2	≤ 84	85 – 89	90 – 92	≥ 93			
AVPU scale				A	V	P	U

Table SM2-3.

National Early Warning Score (NEWS)

	Score						
Variable	3	2	1	0	1	2	3
Heart rate	≤ 40		41 – 50	51 – 90	91 – 110	111 – 130	≥ 131
Resp. rate	≤ 8		9 – 11	12 – 20		21 – 24	≥ 25
Temperature	≤ 35.0		35.1 – 36.0	36.1 – 38.0	38.1 – 39.0	≥ 39.1	
Systolic BP	≤ 90	91 – 100	101 – 110	111 – 219			≥ 220
SpO_2	≤ 91	92 – 93	94 – 95	≥ 96			
Inspired O_2		Any O_2		Air			
AVPU scale				A			V, P, U

Table SM2-4.

Centile-based Early Warning Score (CEWS)

	Score						
Variable	3	2	1	0	1	2	3
Heart rate	≤ 42	43 – 49	50 – 53	54 – 104	105 – 112	113 – 127	≥ 128
Resp. rate	≤ 7	8 – 10	11 – 12	13 – 21	22 – 23	24 – 28	≥ 29
Temperature	≤ 35.4		35.5 – 35.9	36.0 – 37.3	37.4 – 38.3		≥ 38.4
Systolic BP	≤ 83	84 – 90	91 – 100	101 – 157	158 – 167	168 – 184	≥ 185
SpO_2	≤ 84	85 – 90	91 – 93	≥ 94			
Inspired O_2		Any O_2		Air			
AVPU scale				A	V		P, U

References

1. Gardner-Thorpe J, Love N, Wrightson J, et al.: The value of Modified Early Warning Score (MEWS) in surgical in-patients: A prospective observational study. *Ann R Coll Surg Engl* 2006; 88:571–575
2. Paterson R, MacLeod DCD, Thetford D, et al.: Prediction of in-hospital mortality and

For peer review only

length of stay using an early warning scoring system: clinical audit. *Clin Med J R Coll Physicians London* 2006; 6:281–284

3. Smith GB, Prytherch DR, Meredith P, et al.: The ability of the National Early Warning Score (NEWS) to discriminate patients at risk of early cardiac arrest, unanticipated intensive care unit admission, and death. *Resuscitation* 2013; 84:465–470
4. Royal College of Physicians (RCP): National Early Warning Score (NEWS). *Stand Assess acute-illness Sev NHS* 2012; 47
5. Watkinson PJ, Pimentel MAF, Clifton DA, et al.: Manual centile-based early warning scores derived from statistical distributions of observational vital-sign data. *Resuscitation* 2018; 129:55–60

For peer review only

Additional file 3 – Additional results

In this section, we describe in more detail some of the results of the study.

Participants

Figure SM3-1 shows the application of inclusion/exclusion criteria to patient-admissions in order to derive the final cohorts in both organisations included in this study.

Figure SM3-1. Diagram showing the construction of the study cohorts from both organisations (OUH and RBH) included in the study by applying inclusion/exclusion criteria.

ICU-based model (RS_1)

The features computed from the data acquired during the patients' ICU stay that were selected for the first model are detailed in Table SM3-1. A total of 45 features were included in the model from the 161 candidate features.

Calibration plots of the model for both development and validation sets are shown in Figure SM3-2. Figure SM3-3 shows the AUROC values achieved by the first model (RS_1) for predicting the adverse event (combined outcome) within an increasingly high number of days after discharge from ICU. The curves of the AUROC values for each outcome, in-hospital death or ICU re-admission, separately, are also shown.

Table SM3-1. Features computed from information acquired during the patients' ICU stay used in the first model, and the corresponding odds ratio and 95% confidence interval (95% CI) for the odds ratio. The normalisation parameters for each feature (mean and standard deviation, SD) are also shown.

Figure SM3-2. Calibration curves for the first model (RS_1) for predicting a future adverse event after ICU discharge. The left-hand panel shows the calibration curve on the development dataset, the right-hand panel shows the calibration curve of the developed model on the validation dataset. The grey line depicts an ideal calibration, representing a perfect correspondence between the observed events and the predicted events for each decile of score.

Figure SM3-3. Performance of the first model (as given by the AUROC) for prediction of cumulative in-hospital deaths, cumulative ICU re-admissions, and cumulative adverse events up to a cut-off day after ICU discharge. The left-hand panel shows the results using

For peer review only

the development dataset, and the right-hand panel shows the performance using the validation dataset. The bottom graphs show the proportion of each event (N) over time for the three outcomes considered.

Vital-sign based model (RS_2)

For the second scoring system, we considered the construction of a multivariate, (possibly) multimodal model of normality, based on the vital-sign observation sets recorded during the 24 hours that preceded discharge home or during the last post-ICU day with recorded data (which corresponds to the 14th day) if the patient had not been discharged, died or re-admitted to the ICU. These observation sets contain the vital signs from the most stable period of the patient's stay, because these data were acquired when patients are at their most "normal" state after discharge from ICU. This set of "normal" data contains $N = 1{,}082$ 5-dimensional vital-sign vectors. Table SM3-2 gives the corresponding means and standard deviations of this subset of data, used to train the model.

Table SM3-2. Vital signs' mean and standard deviation (SD) values for observations recorded in during the most "stable" period considered.

For peer review only

Figure SM3-1.

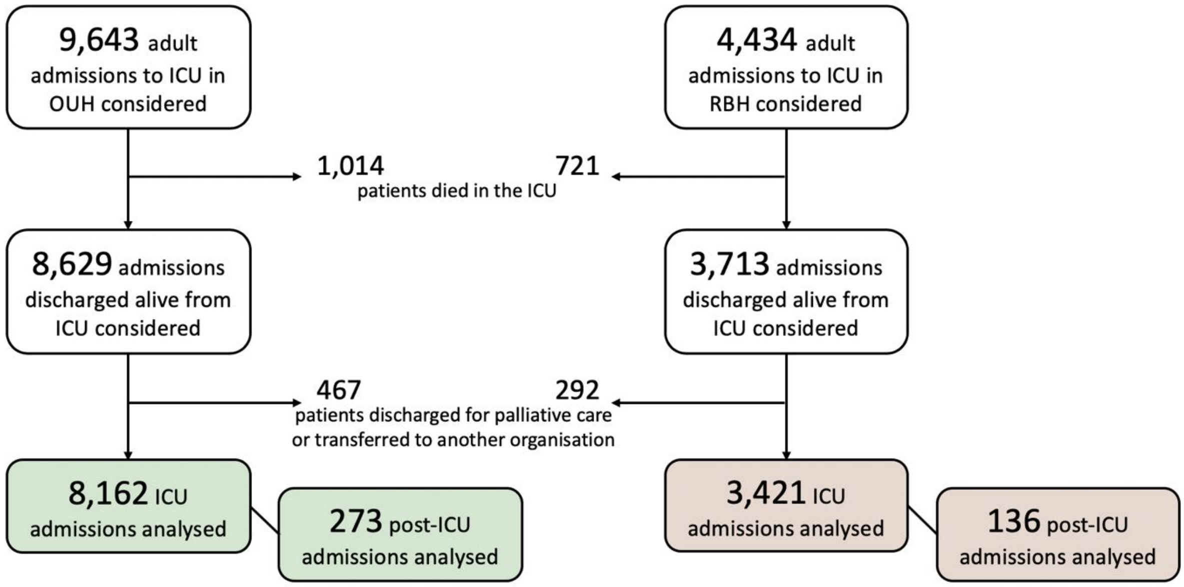

For peer review only

Figure SM3-2.

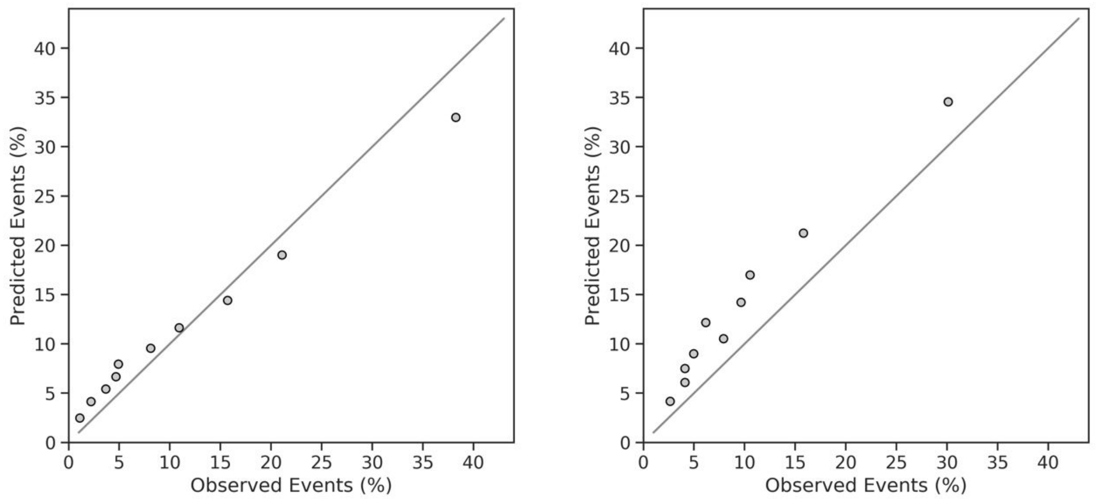

peer review only

Figure SM3-3.

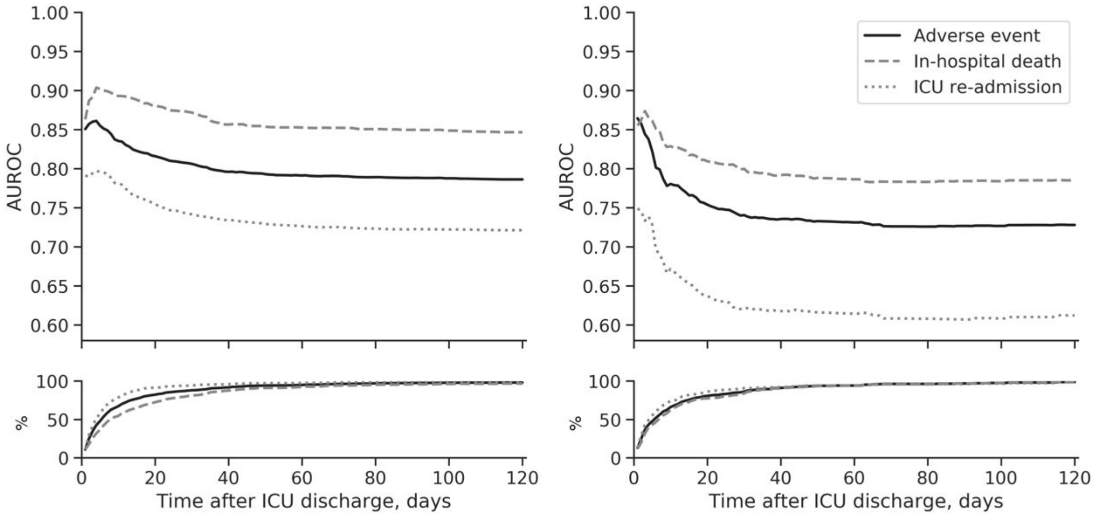
Adverse event
In-hospital death
ICU re-admission
AUROC
%
1.00
0.95
0.90
0.85
0.80
0.75
0.70
0.65
0.60
100
50
0
0
20
40
60
80
100
120
Time after ICU discharge, days

Table SM3-1.

Feature description	Mean	(SD)	Odds ratio	95% CI
**Demographic and administrative information** (3 features)				
Age at admission to ICU	59	(17)	1.305	1.196 to 1.425
Time spend in the ICU, in days	4.3	(19.5)	1.134	1.072 to 1.199
Time from acute hospital admission and ICU admission, in days	4.2	(7.4)	1.038	0.964 to 1.119
**Vital signs related** (13 features)				
Last ventricular heart rate	86	(17)	1.135	1.024 to 1.259
Highest respiratory rate in the last 72 hours	26	(7)	1.129	1.033 to 1.235
Lowest ventricular heart rate in the last 72 hours	70	(16)	1.056	0.927 to 1.202
Highest ventricular heart rate in the first 24 hours	105	(22)	1.039	0.944 to 1.143
Last respiratory rate	18	(5)	1.026	0.943 to 1.116
Lowest ventricular heart rate in the first 24 hours	72	(17)	1.023	0.901 to 1.161
Highest SpO_2 recorded using pulse oximetry in the last 72 hours	100	(1)	1.016	0.944 to 1.094
Lowest respiratory rate in the first 24 hours	12	(3)	1.002	0.925 to 1.085
Lowest temperature in the first 24 hours	37.0	(0.7)	0.984	0.906 to 1.068
Highest temperature in the first 24 hours	35.5	(0.8)	0.981	0.889 to 1.083
Last mean arterial blood pressure	91	(16)	0.921	0.853 to 0.995
Lowest temperature in the last 72 hours	35.5	(0.7)	0.917	0.831 to 1.011
Last total GCS recorded	14	(1)	0.854	0.805 to 0.906
**Laboratory test results related** (24 features)				
Last plasma urea	9.1	(7.1)	1.313	1.186 to 1.453
Last plasma sodium	139	(4)	1.152	1.073 to 1.237
Highest INR in the first 24 hours	1.23	(0.48)	1.063	0.982 to 1.151
Last plasma bilirubin	12.8	(14.8)	1.052	0.984 to 1.124
Highest pH in the first 24 hours	7.43	(0.06)	1.051	0.971 to 1.138
Highest C-reactive protein in the first 24 hours	91	(55)	1.050	0.939 to 1.173
Last plasma lactate	1.16	(0.71)	1.028	0.957 to 1.103
Last INR	1.13	(0.35)	1.027	0.951 to 1.111
Last C-reactive protein	89	(54)	1.009	0.904 to 1.126
Highest platelet count in the first 24 hours	200	(109)	1.006	0.929 to 1.089
Last plasma sodium was in the extremes of the development set range (> or <)	-	-	1.003	0.831 to 1.211
Last plasma creatinine	116	(130)	0.993	0.735 to 1.342
The variability of $AaDO_2$ in the last 72 hours	6.1	(5.1)	0.990	0.908 to 1.080
Highest weight in the first 24 hours	77	(20)	0.989	0.909 to 1.076
Last haematocrit	32	(5)	0.989	0.881 to 1.110
Highest creatinine in the last 24 hours	124	(142)	0.975	0.744 to 1.278

For peer review only

Lowest haemoglobin in the first 24 hours	9.6	(2.0)	0.963	0.864 to 1.075
Highest white blood cell count in the first 24 hours	13.9	(7.0)	0.962	0.893 to 1.036
Lowest plasma potassium in the first 24 hours	3.8	(0.5)	0.951	0.879 to 1.030
Highest plasma creatinine in the last 72 hours	134	(153)	0.950	0.752 to 1.200
Total urine output in the first 24 hours	1772	(1260)	0.937	0.842 to 1.043
Last PaO_2/FiO_2 ratio	43.7	(15.8)	0.904	0.826 to 0.990
Last plasma albumin	28	(6)	0.870	0.798 to 0.949
Total urine output in the last 24 hours	2007	(1293)	0.781	0.703 to 0.867
**Procedures and treatments related (**5 features**)**				
If enteral feeding was used at any point during ICU stay	-	-	1.119	0.946 to 1.323
If a central venous catheter was present at ICU discharge	-	-	1.118	0.957 to 1.306
If an insulin infusion was used in the last 24 hours	-	-	1.055	0.894 to 1.246
If parenteral feeding was used at any point during ICU stay	-	-	1.044	0.799 to 1.363
A dysrhythmia at any point during ICU stay	-	-	1.001	0.999 to 1.003

For peer review only

Table SM3-2.

Vital sign	Mean (SD)
**Heart rate, beats per min.**	83 (14)
**Respiratory rate, breaths per min.**	17 (2)
**Systolic blood pressure, mmHg**	129 (19)
**SpO_2, %**	97 (3)
**Temperature, C**	36.3 (0.5)

For peer review only

Reporting checklist for prediction model development/validation.

Based on the TRIPOD guidelines.

Instructions to authors

Complete this checklist by entering the page numbers from your manuscript where readers will find each of the items listed below.

Your article may not currently address all the items on the checklist. Please modify your text to include the missing information. If you are certain that an item does not apply, please write "n/a" and provide a short explanation.

Upload your completed checklist as an extra file when you submit to a journal.

In your methods section, say that you used the TRIPODreporting guidelines, and cite them as:

Collins GS, Reitsma JB, Altman DG, Moons KG. Transparent reporting of a multivariable prediction model for individual prognosis or diagnosis (TRIPOD): The TRIPOD statement.

		Reporting Item	Page Number
**Title**			
	#1	Identify the study as developing and / or validating a multivariable prediction model, the target population, and the outcome to be predicted.	1
**Abstract**			

For peer review only

	#2	Provide a summary of objectives, study design, setting, participants, sample size, predictors, outcome, statistical analysis, results, and conclusions.	3
**Introduction**			
	#3a	Explain the medical context (including whether diagnostic or prognostic) and rationale for developing or validating the multivariable prediction model, including references to existing models.	4
	#3b	Specify the objectives, including whether the study describes the development or validation of the model or both.	4-8
**Methods**			
Source of data	#4a	Describe the study design or source of data (e.g., randomized trial, cohort, or registry data), separately for the development and validation data sets, if applicable.	5
Source of data	#4b	Specify the key study dates, including start of accrual; end of accrual; and, if applicable, end of follow-up.	5
Participants	#5a	Specify key elements of the study setting (e.g., primary care, secondary care, general population) including number and location of centres.	5
Participants	#5b	Describe eligibility criteria for participants.	5
Participants	#5c	Give details of treatments received, if relevant	N/A

For peer review only

Outcome	#6a	Clearly define the outcome that is predicted by the prediction model, including how and when assessed.	6
Outcome	#6b	Report any actions to blind assessment of the outcome to be predicted.	N/A
Predictors	#7a	Clearly define all predictors used in developing or validating the multivariable prediction model, including how and when they were measured	6
Predictors	#7b	Report any actions to blind assessment of predictors for the outcome and other predictors.	N/A
Sample size	#8	Explain how the study size was arrived at.	N/A
Missing data	#9	Describe how missing data were handled (e.g., complete-case analysis, single imputation, multiple imputation) with details of any imputation method.	5
Statistical analysis methods	#10a	If you are developing a prediction model describe how predictors were handled in the analyses.	7-8
Statistical analysis methods	#10b	If you are developing a prediction model, specify type of model, all model-building procedures (including any predictor selection), and method for internal validation.	7-8
Statistical analysis methods	#10c	If you are validating a prediction model, describe how the predictions were calculated.	7-8
Statistical analysis methods	#10d	Specify all measures used to assess model performance and, if relevant, to compare multiple models.	7-8

For peer review only

Statistical analysis methods	#10e	If you are validating a prediction model, describe any model updating (e.g., recalibration) arising from the validation, if done	7-8
Risk groups	#11	Provide details on how risk groups were created, if done.	N/A
Development vs. validation	#12	For validation, identify any differences from the development data in setting, eligibility criteria, outcome, and predictors.	5
**Results**			
Participants	#13a	Describe the flow of participants through the study, including the number of participants with and without the outcome and, if applicable, a summary of the follow-up time. A diagram may be helpful.	5
Participants	#13b	Describe the characteristics of the participants (basic demographics, clinical features, available predictors), including the number of participants with missing data for predictors and outcome.	Table 1
Participants	#13c	For validation, show a comparison with the development data of the distribution of important variables (demographics, predictors and outcome).	Table 1
Model development	#14a	If developing a model, specify the number of participants and outcome events in each analysis.	8
Model development	#14b	If developing a model, report the unadjusted association, if calculated between each candidate predictor and outcome.	9

For peer review only

Model specification	#15a	If developing a model, present the full prediction model to allow predictions for individuals (i.e., all regression coefficients, and model intercept or baseline survival at a given time point).	9
Model specification	#15b	If developing a prediction model, explain how to the use it.	N/A
Model performance	#16	Report performance measures (with CIs) for the prediction model.	9
Model-updating	#17	If validating a model, report the results from any model updating, if done (i.e., model specification, model performance).	N/A
**Discussion**			
Limitations	#18	Discuss any limitations of the study (such as nonrepresentative sample, few events per predictor, missing data).	11
Interpretation	#19a	For validation, discuss the results with reference to performance in the development data, and any other validation data	9-11
Interpretation	#19b	Give an overall interpretation of the results, considering objectives, limitations, results from similar studies, and other relevant evidence.	9-11
Implications	#20	Discuss the potential clinical use of the model and implications for future research	12

For peer review only

Other information

Supplementary information	#21	Provide information about the availability of supplementary resources, such as study protocol, Web calculator, and data sets.	19
Funding	#22	Give the source of funding and the role of the funders for the present study.	13

The TRIPOD checklist is distributed under the terms of the Creative Commons Attribution License CC-BY. This checklist was completed on 11. April 2023 using https://www.goodreports.org/, a tool made by the EQUATOR Network in collaboration with Penelope.ai

For peer review only

Development of an enhanced scoring system to predict ICU re-admission or in-hospital death within 24h using routine patient data from two NHS Foundation Trusts

Journal:	*BMJ Open*
Manuscript ID	bmjopen-2023-074604.R1
Article Type:	Original research
Date Submitted by the Author:	02-Nov-2023

Complete List of Authors:	Pimentel, Marco; University of Oxford, Department of Engineering Science<br>Johnson, Alistair; Massachusetts Institute of Technology, Institute of Medical Engineering & Science<br>Darbyshire, Julie; Oxford University, Department of Primary Care Health Sciences<br>Tarassenko, Lionel; University of Pittsburgh, Engineering Science<br>Clifton, David A.; Univ Oxford, Department of Engineering Science<br>Walden, Andrew; Royal Berkshire NHS Foundation Trust<br>Rechner, Ian; Royal Berkshire NHS Foundation Trust<br>Young, Duncan; Oxford University, Nuffield Department of Clinical Neurosciences<br>Watkinson, Peter; University of Oxford, Nuffield Department of Clinical Neurosciences

**Primary Subject Heading**:	Intensive care
Secondary Subject Heading:	Health informatics
Keywords:	Clinical Decision-Making, Health informatics < BIOTECHNOLOGY & BIOINFORMATICS, Adult intensive & critical care < INTENSIVE & CRITICAL CARE, STATISTICS & RESEARCH METHODS, CLINICAL PHYSIOLOGY

SCHOLARONE™ Manuscripts

I, the Submitting Author has the right to grant and does grant on behalf of all authors of the Work (as defined in the below author licence), an exclusive licence and/or a non-exclusive licence for contributions from authors who are: i) UK Crown employees; ii) where BMJ has agreed a CC-BY licence shall apply, and/or iii) in accordance with the terms applicable for US Federal Government officers or employees acting as part of their official duties; on a worldwide, perpetual, irrevocable, royalty-free basis to BMJ Publishing Group Ltd ("BMJ") its licensees and where the relevant Journal is co-owned by BMJ to the co-owners of the Journal, to publish the Work in this journal and any other BMJ products and to exploit all rights, as set out in our licence.

The Submitting Author accepts and understands that any supply made under these terms is made by BMJ to the Submitting Author unless you are acting as an employee on behalf of your employer or a postgraduate student of an affiliated institution which is paying any applicable article publishing charge ("APC") for Open Access articles. Where the Submitting Author wishes to make the Work available on an Open Access basis (and intends to pay the relevant APC), the terms of reuse of such Open Access shall be governed by a Creative Commons licence – details of these licences and which Creative Commons licence will apply to this Work are set out in our licence referred to above.

Other than as permitted in any relevant BMJ Author's Self Archiving Policies, I confirm this Work has not been accepted for publication elsewhere, is not being considered for publication elsewhere and does not duplicate material already published. I confirm all authors consent to publication of this Work and authorise the granting of this licence.

For peer review only

Title page

Development of an enhanced scoring system to predict ICU re-admission or in-hospital death within 24h using routine patient data from two NHS Foundation Trusts

Authors:

Marco AF Pimentel, Institute of Biomedical Engineering, Department of Engineering Science, University of Oxford, Oxford, UK

Alistair Johnson, Institute of Medical Engineering & Science, Massachusetts Institute of Technology, Cambridge, Massachusetts, USA.

Julie L Darbyshire, Nuffield Department of Clinical Neurosciences, University of Oxford, Oxford, UK

Lionel Tarassenko, Institute of Biomedical Engineering, Department of Engineering Science, University of Oxford, Oxford UK

David A Clifton, Institute of Biomedical Engineering, Department of Engineering Science, University of Oxford, Oxford UK

Andrew Walden, Intensive Care Unit, Royal Berkshire NHS Foundation Trust, Reading, UK

Ian Rechner, Intensive Care Unit, Royal Berkshire NHS Foundation Trust, Reading, UK

J Duncan Young, Nuffield Department of Clinical Neurosciences, University of Oxford, Oxford, UK

Peter J Watkinson, Nuffield Department of Clinical Neurosciences, University of Oxford, Oxford, UK

Corresponding author:
Julie L Darbyshire
Nuffield Department of Primary Care Health Sciences, University of Oxford
Oxford
OX2 6GG
UK
julie.darbyshire@phc.ox.ac.uk

Running head:
Early warning scores after ICU

Descriptor number:
4.6

For peer review only

Word count:
Body: ~3,300
Abstract: 300
Tables: 3
Figures: 2

At a Glance Commentary:

Scientific Knowledge on the Subject

Early Warning Score (EWS) systems are used to identify hospitalised patients at risk of adverse events. These systems almost all use vital signs to determine this risk. Adding further data from electronic patient records may improve the performance of these systems. Patients discharged from an intensive care unit have both a comprehensive electronic patient record and a high risk of future adverse events, so they are an ideal patient group to test if additional data improves the system performance.

What This Study Adds to the Field

The study shows a scoring system incorporating data from a patient's stay in an intensive care unit has better performance than typically-used EWS systems based on vital signs alone. The score developed in this study uses a machine learning method to estimate future risk.

This article has additional files.

For peer review only

Abstract

Rationale. Intensive care units (ICUs) admit the most severely ill patients. Once these patients are discharged from the ICU to a step-down ward, they continue to have their vital signs monitored by nursing staff, with early warning score (EWS) systems being used to identify those at risk of deterioration.

Objectives. We report the development and validation of an enhanced continuous scoring system for predicting adverse events, which combines vital signs measured routinely on acute care wards (as used by most EWSs) with a risk score of a future adverse event calculated on discharge from ICU.

Design. A modified Delphi process identified candidate variables commonly available in electronic records as the basis for a ‘static’ score of the patient’s condition immediately after discharge from the ICU. L1-regularised logistic regression was used to estimate the in-hospital risk of future adverse event. We then constructed a model of physiological normality using vital-sign data from the day of hospital discharge. This is combined with the static score and used continuously to quantify and update the patient’s risk of deterioration throughout their hospital stay.

Setting: Data from two NHS Foundation Trusts (UK) were used to develop and (externally) validate the model.

Participants: A total of 12,394 vital-sign measurements were acquired from 273 patients after ICU discharge for the development set, and 4,831 from 136 patients in the validation cohort.

Results. Outcome validation of our model yielded an area under the receiver operating characteristic curve (AUROC) of 0.724 for predicting ICU re-admission or in-hospital death within 24h. It showed an improved performance with respect to other competitive risk scoring systems, including the National EWS (NEWS, 0.653).

Conclusions. We showed that a scoring system incorporating data from a patient’s stay in ICU has better performance than commonly-used EWS systems based on vital signs alone.

Keywords: early warning score; intensive care; physiological deterioration; patient monitoring.

Strengths and Limitations

* Over 17,000 vital sign measurements from >400 patients included in the cohort

For peer review only - http://bmjopen.bmj.com/site/about/guidelines.xhtml

* Variable selection informed by systematic review and Delphi process prior to data extraction
* Data extraction variables limited to those available
* Performance assessed using common methology for early warning score systems
* Compound outcome (including in-hospital mortality and ICU re-admission) limits prediction of inevitable death and improves prediction of preventable deaths

For peer review only

Introduction

Patients hospitalised for acute conditions can suffer adverse events such as cardiac arrests or unplanned admission to a higher-acuity area. These events are usually preceded by changes in vital signs some hours before (1–5). Early detection of these changes might prevent some of the subsequent adverse events, so many simple Early Warning Score (EWS) systems have been developed and deployed clinically (6–8). EWS systems are now recommended to be used as part of routine care by the UK National Institute for Health and Clinical Excellence (9–11) and are widely used in other healthcare settings.

EWS systems initially used purpose-designed paper charts (12, 13). An increasing number of healthcare providers now use electronic systems (14, 15) to gather physiological data and calculate scores (16). With the increasing use of electronic patient records, there is scope to develop improved EWS systems that include many more variables than are currently used, potentially leading to increasing predictive accuracy by using more complex algorithms to calculate scores (17).

We hypothesise that an EWS system could better predict adverse events if it included variables from a patient's electronic medical record. As a proof of concept, we studied patients discharged to acute care wards from intensive care units (ICUs). These patients have a detailed electronic patient record (EPR) containing data about their ICU stay, and typically having a high rate of adverse events occurring after discharge to a ward. In addition, there is a large body of literature describing variables that are correlated with adverse events in these patients.

This study describes the development of an enhanced scoring system for predicting adverse events in patients discharged from ICUs which combines (i) routine vital signs measured on acute care wards (as used by most EWS systems) with (ii) a risk score of a future adverse event calculated on discharge from ICU. The study further validates the ability of the proposed scoring system and other published EWS systems to identify patients at risk of death or re-admission to ICU.

Materials and Methods

This study is reported in line with the TRIPOD statement (18).

Regulatory approvals

The project had UK Health Research Authority Confidentiality Advisory Group approval (ref: ECC 7-05(f)/2011), National Research Ethics Service Committee approval (ref: 11/SC/0440) for retrospective data collection and analysis, and National Research Ethics Service Committee approval (ref: 12/SC/0357) for prospective data collection and analysis.

For peer review only - http://bmjopen.bmj.com/site/about/guidelines.xhtml

Data sources

Data from two NHS Foundation Trusts (UK) were used in this study. The Oxford University Hospitals NHS Foundation Trust (OUH) has two adult general ICUs. The Royal Berkshire Hospital NHS Foundation Trust (RBH) in Reading, UK, has a single adult general ICU.

We used routinely-collected data, stored in the ICU computerised information systems (CIS). The OUH and RBH CIS contain all measurements of physiological status, and other relevant clinical information such as patient demographics; details of treatments and interventions; and laboratory test results recorded during a patient's ICU stay. An average of 655 data items are recorded daily for each patient. All the ICUs in this study use a Philips Healthcare CIS (Philips Healthcare, Eindhoven, Netherlands). In addition we used the linked datasets submitted to the UK national comparative audit for ICUs, or ICNARC CMP (19).

During the study period, neither organisation had electronic recording of vital signs for patients in acute care wards outside the ICUs. Vital-sign data were collected prospectively for recruited patients in both organisations for the first 14 days after discharge from ICU. The physiological data from each patient's paper observation chart were entered into an electronic database. Data were double-entered in 55% of patients to confirm the accuracy of data entry.

All analyses were conducted on anonymised data.

Participants

All completed adult admissions to OUH ICUs from June 2006 to December 2015 and to the RBH ICU from April 2010 to December 2015 were used to develop the ICU discharge score. Admissions were only considered when (i) patients were discharged alive from ICU and (ii) the discharge status of the patient episode (or hospital admission) was known (or recorded). We excluded patients discharged from ICU for palliative care or transferred to another organisation. Vital signs data on wards were acquired prospectively after discharge from ICU for patients in OUH between April 2013 and December 2014, and in RBH from October 2013 and December 2014.

After exclusion criteria were applied, we split the database into “development” and “validation” sets by organisation, such that (1) we developed and evaluated the model using data from all valida admissions within the OUH group using cross-validation methods, and (2) we performed an external validation of the model using data from all valid admissions within the RBH group.

For peer review only - http://bmjopen.bmj.com/site/about/guidelines.xhtml

Outcomes

The primary outcome was the first occurrence of either in-hospital death or re-admission to the ICU. For the evaluation of EWS systems, we considered the compound outcome of in-hospital death or ICU re-admission within the next *N* hours of a vital-sign observation, in line with previous studies (20, 21). For the primary outcome, we set *N = 24* hours. We have also evaluated the systems for different values of *N* (as detailed below). Secondary outcomes were in-hospital death or re-admission to ICU, individually.

Predictors

We used a conceptual model in which we estimated the risk of a patient experiencing an adverse event at the point of discharge from ICU from variables recorded during their stay in the ICU. After discharge from ICU, the patient's risk of experiencing an adverse event within the following 24h was calculated using both ward-recorded vital signs and the risk calculated at ICU discharge.

ICU-based feature representation:
We used an evidence-based technique to select candidate variables to calculate the risk of deterioration at ICU discharge. We systematically reviewed of studies reporting a significant ($p < 0.05$) association between a variable recorded during an ICU stay and either in-hospital death or ICU re-admission. The resulting list of candidate variables was reviewed by a panel of five clinical experts in a modified Delphi process who added other variables they expected to be predictive of adverse events. Candidate variables were then limited to those available in our electronic databases. These were either "static" variables (mainly based on demographic information) or time-varying variables recorded repeatedly throughout the patient's stay in the ICU (see Figure 1). To determine the risk of future compound outcome after discharge from the ICU, we derived a total of 161 candidate features from all candidate variables, which were then used for building a prediction model.

Post-ICU feature representation:
All vital-sign observations recorded for 14 days after discharge from ICU were considered for analysis. Each set of vital signs includes heart rate, systolic blood pressure, respiratory rate, body temperature, neurological status assessment using the Alert-Verbal-Painful-Unresponsive (AVPU) scale, peripheral oxygen saturation (SpO_2), a record of whether the patient was receiving supplemental oxygen at the time of the SpO_2 measurement, and the date and time of the observation. Vital-sign measurements are typically recorded every 4 or 6h throughout the patient's stay on the ward (see Figure 1).

The final list of candidate variables and features, and procedures for pre-processing (including dealing with missing data) are further described [see Additional file 1].

For peer review only

Model development

To develop the risk scoring system, our approach assumes that at each vital-sign observation performed after discharge from ICU, the patient's current condition can be characterised (or represented) by a single risk estimate. Immediately after ICU discharge, the patient is assigned a risk score (RS_1) estimated from an ICU-based set of features, which is then updated using the abnormalities in their vital signs recorded during subsequent ward care (RS_2).

We therefore built the first model, RS_1, using the features derived from the variables acquired during the patients' stay in the ICU using the development set. We used a L1-regularised logistic regressor for predicting the compound outcome. For the second scoring system, RS_2, we applied a one-class classification method (22) using the vital-sign data recorded after discharge from the ICU, as described previously (23, 24). Further details of the model development are available [see Additional file 1].

An overall risk score, the risk score index (RSI), was subsequently determined using a simple time-dependent linear combination of the two constituent risk scores, such that:

$$\mathrm{RSI} = \beta\left[\left(1 - \frac{t}{Tmax}\right)\mathrm{RS}_1\right] + \left[\left(\frac{t}{Tmax}\right)\mathrm{RS}_2\right] \quad \text{(Equation 1)}$$

where β is used to adjust the weight of RS_1 with respect to the time since discharge from ICU, t corresponds to the elapsed time (in hours) since the patient was discharged from ICU and has a maximum value of $Tmax$ hours. Further details of the model development and optimisation of the parameters are available [see Additional file 1].

Model validation and statistical analysis

The discrimination of the first model, RS_1, was assessed using the area under the curve of the receiver operating characteristic (AUROC) metric. Calibration was assessed using a goodness of fit test, the Hosmer-Lemeshow "C" statistic, the Brier score and Cox's calibration regression (25–27). The performance of this first model was examined both for the compound outcome and each adverse event (in-hospital death and ICU re-admission) individually. The ability of RS_1 to predict future adverse events at increasing intervals from ICU discharge was also examined by calculating the AUROC for future events by day after (ICU) discharge (up to 120 days).

The final model (RSI) was validated using the AUROC for the compound outcome of in-hospital death or ICU re-admission within the next *N* hours of a vital-sign measurement recorded after ICU discharge, in line with previous studies for

For peer review only - http://bmjopen.bmj.com/site/about/guidelines.xhtml

evaluating EWS systems (20, 21). We evaluated the model for different values of *N*, with $N = [12, 24, 36, 48, 72]$ hours. We note that in this case the AUROC represents how well the scoring system RSI discriminates between observation sets followed by an adverse event and those with no subsequent adverse outcome within the next *N* hours. Therefore, the unit of analysis is a vital-sign set rather than a patient-admission, as performed for the validation of the first model.

We also considered each individual adverse event separately. To understand better the feasibility of implementating the risk scoring systems in this setting, we also evaluated the burden of observation sets “triggered” by the risk scoring system for every correctly identified observation followed by an adverse event within 24h.

We report the cross-validation results using the development dataset. We also report the external validation results using data from the RBH Trust. Confidence intervals were estimated using bootstrap confidence intervals via percentiles, with 500 samples (28).

Comparison with published risk scoring systems

We compared the performance of our proposed risk scoring index, RSI, with that of each model individually (RS_1 and RS_2), and with that of a number of published and clinically-used EWS systems: the modified EWS, or MEWS (29), the standardised EWS, or SEWS (30), the National EWS, or NEWS (21), and our centile-based EWS, or CEWS (31). We detail the components and weightings of the individual EWS systems in Additional file 2.

Patient and Public Involvement statement

The PICRAM study (2011 – 2015) had no embedded PPI in either conceptualisation or delivery of the work.

Results

The application of inclusion and exclusion criteria to patient-admissions in order to derive the final cohorts in both organisations included in this study is shown in Figure SM3-1 [see Additional file 3]. A total of 8,162 admissions to ICU were included in the development dataset. Vital-sign observations were prospectively collected for 273 patients (from the 8,162 ICU admissions) after discharge from the ICU. The validation dataset included 3,421 ICU admissions, from which 136 had data acquired during the post-ICU period. Table 1 provides a summary of characteristics of the development and validation cohorts derived from the ICU admissions. Both cohorts were similar in terms of demographic and administrative data. The compound outcome rate was slightly higher in the development dataset than that in the validation dataset, considering either all ICU admissions or the sub-group of post-ICU admissions. We also note that the number of in-hospital deaths is higher than

For peer review only - http://bmjopen.bmj.com/site/about/guidelines.xhtml

the number of re-admissions to ICU. In the development cohort, 12,394 vital-sign observation sets were acquired from the 273 patients after ICU discharge, and 4,831 from the 136 patients in the validation cohort.

For the first model (RS_1), which estimates the risk of future adverse events immediately after ICU discharge, 45 features from the 161 candidate features identified from the systematic review and expert opinion, were retained in the final model. These are listed Table SM3-1 [see Additional file 3]. They comprise largely measures of cardiac or respiratory physiology, renal and hepatic function, plasma electrolytes, measures of inflammation and measures of treatment intensity. Of note, artificial ventilation during the last 24h of ICU admission was associated with a lower risk of adverse outcome. This is because all the ICUs admit ventilated, elective, post-surgical admissions who have a low risk of adverse events and are discharged within 24h of extubation. Calibration plots of the model for both development and validation sets are shown in Figure SM3-2 [see Additional file 3]. Table 2 summarises the performance for the combined outcome and for either adverse event (in-hospital death or ICU re-admission) considered individually.

Table 3 shows the performance of RSI and other baseline scoring systems for predicting observation sets followed by in-hospital death or ICU re-admission within the following 24h. The proposed scoring system showed an increased discrimination ability to predict adverse events within 24h with respect to the other risk scoring systems considered in this study. Using the external validation dataset (RBH), RSI gave an AUROC of 0.724 (95% CI of 0.704-0.741), versus 0.653 (0.621-0.683) for NEWS, and 0.672 (0.648-0.695) for CEWS. Figure 2 shows the AUROC values of the risk scoring systems for predicting the compound outcome within 12, 24, 36, 48, and 72h of a vital-sign measurement. The proposed RSI system consistently shows superior discrimination for each derived outcome.

Discussion

There are many variants of the original EWS system (6–8), including systems designed for specific patient groups such as children (32, 33) and patients in high-dependency units (34). These systems typically use vital signs to determine the level of risk of an adverse event. Vital signs are used because they are easily-acquired variables, regularly measured in clinical practice, and have a long history of being used to track patients' progression over time. However, point estimates of the risk of future deterioration from single vital-sign measurement sets assume "normal" values that may not be appropriate for a hospital population, and ignore other data that can add to the precision and granularity of the risk estimate. The introduction of electronic recording of vital signs and electronic patient records means that the parsimony and simplicity required by paper-based systems are now less relevant.

For peer review only - http://bmjopen.bmj.com/site/about/guidelines.xhtml

Main findings and strengths

This was a proof-of-concept study for developing and validating an enhanced EWS that uses many more electronically held variables than the conventional vital signs, and which combined dynamic and static methods of risk evaluation, as typically used in other prediction disciplines such as meteorology (35) and imaging (36). We chose to study post-ICU patients because they have a detailed electronic record generated during their ICU stay, a high adverse event rate after ICU discharge, and because a significant body of literature exists on variables associated with adverse outcomes. However, the design principles we used in this study can be applied to any acute care patient group where sufficient data are available electronically.

As part of this work we developed a new scoring system for predicting in-hospital mortality and re-admission to ICU from data collected during a patient's ICU stay. A systematic review in 2013 (37) identified seven different published scoring systems that quantify the risk of mortality or re-admission at varying intervals after ICU discharge. However, only two studies verified their systems with an external (independent) validation dataset (38, 39). They obtained AUROC values similar to those found in this study.

RSI is a dynamic system that computes an updated risk estimate every time a new vital sign is recorded. It combines this dynamic risk estimate derived from the routinely-measured vital signs on the acute ward (RS_2) with a "static" risk estimate that is computed immediately after discharge from ICU using data from the ICU CIS (RS_1). As the performance of RS_1 is expected to worsen as the time from discharge from ICU increases because patients' conditions change (see Figure SM3-3), we used a time-dependent function to reduce its contribution over time, with the patient's risk score becoming increasingly determined by vital-signs based RS_2. The combined risk estimates as given by RSI improved the predictive power of our scoring system when compared with RS_2.

In this study, a compound outcome of in-hospital mortality and ICU re-admission was used as opposed to only using in-hospital mortality (20). Patients discharged from ICU with curative intent may die in hospital in spite of full, timely and appropriate care. They may also die because they do not receive the care they need in a timely fashion. Developing a model using in-hospital mortality as the sole outcome would limit the power of such a model, as appropriately-treated survivors, with a re-admission to ICU, would not contribute to the adverse events. Hence, there would be a risk that the resulting model would predict inevitable, rather than preventable, deaths, as also noted by others (40, 41). Our main goal was to develop a system that would identify patients who were likely to respond to earlier intervention of higher acuity care. Whilst ICU re-admission does not capture all the appropriately treated survivors, there is no other marker readily available in hospital electronic

For peer review only

records to identify these patients. A similar compound outcome has been used in validation studies of EWSs for the same reason (21, 31, 42). We further note that RSI was considerably better at predicting in-hospital mortality than ICU re-admission (see Table 3). This has also been noted in other studies reporting evaluation of EWS systems (21, 42, 43), where ICU admissions are less reliably identified than in-hospital death.

Limitations

This work has a number of limitations. We studied a very specific group of patients who were at high risk of in-hospital deterioration and who had detailed records of their ICU stay before they were discharged to the ward. We could only study the variables available in the ICU electronic records; hence, some of the candidate variables identified in the systematic review could not be included in the model. In addition, as vital-sign data had to be prospectively collected from consenting patients and transcribed from paper charts, the number of patients in the study, and therefore the number of adverse events, was limited.

We assessed the performance of the combined scoring system developed using the methodology commonly used to assess that of EWS systems. This uses AUROC measures based on paired vital-sign recordings and events within fixed time periods; i.e., derived outcome of occurrence of an adverse event within (e.g.) 24h of a vital-sign measurement. Therefore, AUROC values represent the probability that any randomly-chosen observation followed within the chosen time period by in-hospital death or ICU re-admission has a higher risk score than any randomly-chosen observation not followed by an event in the same time period (44). That is, repeated measurements from the same patient were used for evaluating the performance of scoring systems, which assumes that the scores computed from each observation set for that patient are independent (which is the usual approach when evaluating EWS systems). However, this assumption may not hold in practice; i.e., a vital-sign measurement at one point in time is likely to be correlated with previous measurements. This, alongside the highly imbalanced dataset (in which the outcome occurs infrequently), gives rise to AUROCs with high values that are not truly comparable with AUROCs from single predictor/single outcome algorithms where the unit of analysis is a patient-admission (45).

An external (independent) dataset was used to validate the risk scoring systems. RSI did not perform as well as expected for the combined outcome. This was primarily because the combined system over-estimated the risk of adverse events in higher-risk patients, possibly reflecting a difference in the patient population at the two hospital sites (46). The ICUs at the OUH admit tertiary referral patients not seen in RBH; hence, risk associated with these patients captured in the scoring system built with the development dataset may not have added explanatory power for patient

For peer review only - http://bmjopen.bmj.com/site/about/guidelines.xhtml

admissions in the validation dataset.

Finally, we note that the performance of our scoring system for predicting adverse events exceeds that of previously published EWS algorithms. This should, however, be interpreted with caution, as most of these systems were developed and validated on all hospital admissions to acute care areas, and our system was developed on a very specific population.

Conclusions

Scoring systems, such as EWSs, are used to identify hospitalised patients at risk of adverse events. In this study, we developed a bipartite score based on machine learning that encompasses the patient state at the time of ICU discharge, as well as vital signs recorded on the wards at the time the risk score is calculated. We showed that a scoring system incorporating data from a patient’s stay in an ICU has better performance than typically-used EWS systems based on vital signs alone.

For peer review only

Ethical Approval and Consent to participate

The project had UK Health Research Authority Confidentiality Advisory Group approval (ref: ECC 7-05(f)/2011), National Research Ethics Service Committee approval (ref: 11/SC/0440) for retrospective data collection and analysis, and National Research Ethics Service Committee approval (ref: 12/SC/0357) for prospective data collection and analysis.

Consent for publication

Not applicable.

Availability of data and materials

Fully anonymised individual participant data may be made available to researchers directly affiliated to an academic institution or charitable organisation who provide an independently-reviewed, methodologically sound proposal. The data will be available for a period starting six months after publication and ending one year later. Only data required to answer the primary question posed by the proposed research will be made available. Researchers should contact Professor Young in the first instance. Other data and outputs from the project (Health Innovation Challenge Fund ref: HICF-0510-006) may be available on request.

Competing interests

JDY, PJW, and LT report grants from Wellcome Trust/UK Department of Health. PJW and LT report grants from National Institute for Health Research, personal fees from Sensyne Health, outside the submitted work.

Funding

This study was supported by the Department of Health and Wellcome Trust through the Health Innovation Challenge Fund (ref: HICF-0510-006). The views expressed in this publication are those of the authors and not necessarily those of the Department of Health & Social Care or Wellcome Trust. PJW, DAC and LT are supported by the NIHR Biomedical Research Centre Oxford. MAFP is supported by a Drayson Research Fellowship.

For peer review only

Author contributions

David A Clifton: Substantial contributions to the conception and design of the work, and analysis and interpretation of data for the work. Revising this manuscript critically for important intellectual content. Final approval of the manuscript. Agreement to be accountable for all aspects of the work.

Julie L Darbyshire: Substantial contributions to the design of the work. Revising this manuscript critically for important intellectual content. Final approval of the manuscript. Agreement to be accountable for all aspects of the work.

Alistair Johnson: Substantial contributions to the acquisition and analysis of data for the work. Revising this manuscript critically for important intellectual content. Final approval of the manuscript. Agreement to be accountable for all aspects of the work.

Marco A F Pimentel: Substantial contributions to the acquisition, analysis and interpretation of data for the work. Revising this manuscript critically for important intellectual content. Final approval of the manuscript. Agreement to be accountable for all aspects of the work.

Ian Rechner: Substantial contributions to the acquisition of data for the work. Revising this manuscript critically for important intellectual content. Final approval of the manuscript. Agreement to be accountable for all aspects of the work.

Lionel Tarassenko: Substantial contributions to the conception and design of the work. Substantial contributions to the acquisition, analysis, and interpretation of data for the work. Drafting the work and revising it critically for important intellectual content. Final approval of the version to be published. Agreement to be accountable for all aspects of the work.

Andrew Walden: Substantial contributions to the acquisition of data for the work. Revising this manuscript critically for important intellectual content. Final approval of the manuscript. Agreement to be accountable for all aspects of the work.

Peter J Watkinson: Substantial contributions to the conception and design of the work. Substantial contributions to the acquisition, analysis, and interpretation of data for the work. Drafting the work and revising it critically for important intellectual content. Final approval of the version to be published. Agreement to be accountable for all aspects of the work.

J Duncan Young: Substantial contributions to the conception and design of the work. Substantial contributions to the acquisition, analysis, and interpretation of data for the work. Drafting the work and revising it critically for important intellectual content. Final approval of the version to be published. Agreement to be accountable for all aspects of the work.

For peer review only

Acknowledgements

Dr Jonathan Burgess, who sadly passed away before seeing the fruits of his labours, undertook much of the early work on the identification of candidate variables in this project.

For peer review only

References

1. Schein RMH, Hazday N, Pena M, et al.: Clinical Antecedents to In-Hospital Cardiopulmonary Arrest. *Chest* 1990; 98:1388–1392

2. McQuillan P, Pilkington S, Allan A, et al.: Confidential inquiry into quality of care before admission to intensive care. *BMJ* 1998; 316:1853–1858

3. Kause J, Smith G, Prytherch D, et al.: A comparison of Antecedents to Cardiac Arrests, Deaths and EMergency Intensive care Admissions in Australia and New Zealand, and the United Kingdom—the ACADEMIA study. *Resuscitation* 2004; 62:275–282

4. Churpek MM, Yuen TC, Huber MT, et al.: Predicting cardiac arrest on the wards: A nested case-control study. *Chest* 2012;

5. Berlot G, Pangher A, Petrucci L, et al.: Anticipating events of in-hospital cardiac arrest. *Eur J Emerg Med* 2004; 11:24–28

6. Morgan R, Williams F, Wright M: An early warning scoring system for detecting developing critical illness. *Clin Intensive Care* 1997; 8:100

7. Smith GB, Prytherch DR, Schmidt PE, et al.: Review and performance evaluation of aggregate weighted “track and trigger” systems. *Resuscitation* 2008;

8. Alam N, Hobbelink EL, van Tienhoven AJ, et al.: The impact of the use of the Early Warning Score (EWS) on patient outcomes: A systematic review. *Resuscitation* 2014; 85:587–594

9. National Institute for Health and Care Excellence (NICE): Acutely ill adults in hospital: recognising and responding to deterioration. *Clin Guidel* 2007;

10. Adam S, Odell M: An acute problem? A report of the National Confidential Enquiry into Patient Outcome and Death. *Nurs Crit Care* 2005; 10:225–227

11. National Institute for Health and Care Excellence (NICE): Sepsis: recognition, diagnosis and early management. *BJU Int* 2018; 121:497–514

12. Christofidis MJ, Hill A, Horswill MS, et al.: Observation charts with overlapping blood pressure and heart rate graphs do not yield the performance advantage that health professionals assume: an experimental study. *J Adv Nurs* 2014; 70:610–624

13. Elliott D, Allen E, Perry L, et al.: Clinical user experiences of observation and response charts: focus group findings of using a new format chart incorporating a track and trigger system. *BMJ Qual Saf* 2015; 24:65–75

14. Smith GB, Prytherch DR, Schmidt P, et al.: Hospital-wide physiological surveillance-A new approach to the early identification and management of the sick patient. *Resuscitation* 2006;

15. Wong D, Bonnici T, Knight J, et al.: SEND: a system for electronic notification and documentation of vital sign observations. *BMC Med Inform Decis Mak* 2015; 15:68

16. Bleyer AJ, Vidya S, Russell GB, et al.: Longitudinal analysis of one million vital

For peer review only - http://bmjopen.bmj.com/site/about/guidelines.xhtml

signs in patients in an academic medical center. *Resuscitation* 2011;

17. Green M, Lander H, Snyder A, et al.: Comparison of the Between the Flags calling criteria to the MEWS, NEWS and the electronic Cardiac Arrest Risk Triage (eCART) score for the identification of deteriorating ward patients. *Resuscitation* 2018; 123:86–91
18. Moons KGM, Altman DG, Reitsma JB, et al.: Transparent Reporting of a multivariable prediction model for Individual Prognosis Or Diagnosis (TRIPOD): Explanation and Elaboration. *Ann Intern Med* 2015; 162:W1
19. Intensive Care National Audit & Research Centre (ICNARC): About the CMP. *Accessed from https//www.icnarc.org/Our-Audit/Audits/Cmp/About; Accessed July 2020*
20. Prytherch DR, Smith GB, Schmidt PE, et al.: ViEWS-Towards a national early warning score for detecting adult inpatient deterioration. *Resuscitation* 2010; 81:932–937
21. Smith GB, Prytherch DR, Meredith P, et al.: The ability of the National Early Warning Score (NEWS) to discriminate patients at risk of early cardiac arrest, unanticipated intensive care unit admission, and death. *Resuscitation* 2013; 84:465–470
22. Pimentel MAF, Clifton DA, Clifton L, et al.: A review of novelty detection. *Signal Processing* 2014; 99:215–249
23. Johnson AEW, Burgess J, Pimentel MAF, et al.: Physiological trajectory of patients pre and post ICU discharge. In: 2014 36th Annual International Conference of the IEEE Engineering in Medicine and Biology Society. IEEE; 2014. p. 3160–3163.
24. Pimentel MAF, Clifton DA, Clifton L, et al.: Modelling physiological deterioration in post-operative patient vital-sign data. *Med Biol Eng Comput* 2013;
25. Lemeshow S, Hosmer DW: A review of goodness of fit statistics for use in the development of logistic regression models. *Am J Epidemiol* 1982;
26. Kramer AA, Zimmerman JE: Assessing the calibration of mortality benchmarks in critical care: The Hosmer-Lemeshow test revisited*. *Crit Care Med* 2007; 35:2052–2056
27. Cox DR: Two further applications of a model for binary regression. *Biometrika* 1958; 45:562–565
28. DiCiccio TJ, Efron B: Bootstrap confidence intervals. *Stat Sci* 1996;
29. Gardner-Thorpe J, Love N, Wrightson J, et al.: The value of Modified Early Warning Score (MEWS) in surgical in-patients: A prospective observational study. *Ann R Coll Surg Engl* 2006; 88:571–575
30. Paterson R, MacLeod DCD, Thetford D, et al.: Prediction of in-hospital mortality and length of stay using an early warning scoring system: clinical audit. *Clin Med J R Coll Physicians London* 2006; 6:281–284
31. Watkinson PJ, Pimentel MAF, Clifton DA, et al.: Manual centile-based early warning scores derived from statistical distributions of observational vital-sign data. *Resuscitation* 2018; 129:55–60

For peer review only

32. Akre M, Finkelstein M, Erickson M, et al.: Sensitivity of the Pediatric Early Warning Score to Identify Patient Deterioration. *Pediatrics* 2010; 125:e763–e769

33. Skaletzky SM, Raszynski A, Totapally BR: Validation of a Modified Pediatric Early Warning System Score. *Clin Pediatr (Phila)* 2012; 51:431–435

34. Hravnak M, Devita MA, Clontz A, et al.: Cardiorespiratory instability before and after implementing an integrated monitoring system. *Crit Care Med* 2011;

35. Gneiting T, Ranjan R: Combining predictive distributions. *Electron J Stat* 2013;

36. Akhondi-Asl A, Hoyte L, Lockhart ME, et al.: A logarithmic opinion pool based STAPLE algorithm for the fusion of segmentations with associated reliability weights. *IEEE Trans Med Imaging* 2014;

37. Hosein FS, Bobrovitz N, Berthelot S, et al.: A systematic review of tools for predicting severe adverse events following patient discharge from intensive care units. *Crit Care* 2013;

38. Daly K: Reduction in mortality after inappropriate early discharge from intensive care unit: logistic regression triage. *BMJ* 2001; 322:1274–1274

39. Fernandez R, Serrano JM, Umaran I, et al.: Ward mortality after ICU discharge: A multicenter validation of the Sabadell score. *Intensive Care Med* 2010;

40. Tarassenko L, Clifton DA, Pinsky MR, et al.: Centile-based early warning scores derived from statistical distributions of vital signs. *Resuscitation* 2011; 82:1013–1018

41. Churpek MM, Yuen TC, Edelson DP: Predicting clinical deterioration in the hospital: The impact of outcome selection. *Resuscitation* 2013; 84:564–568

42. Redfern OC, Pimentel MAF, Prytherch D, et al.: Predicting in-hospital mortality and unanticipated admissions to the intensive care unit using routinely collected blood tests and vital signs: Development and validation of a multivariable model. *Resuscitation* 2018; 133:75–81

43. Subbe CP: Validation of a modified Early Warning Score in medical admissions. *QJM* 2001; 94:521–526

44. Jarvis SW, Kovacs C, Briggs J, et al.: Are observation selection methods important when comparing early warning score performance? *Resuscitation* 2015; 90:1–6

45. Romero-Brufau S, Huddleston JM, Escobar GJ, et al.: Why the C-statistic is not informative to evaluate early warning scores and what metrics to use. *Crit Care* 2015; 19:285

46. Subbaswamy A, Schulam P, Saria S: Preventing Failures Due to Dataset Shift: Learning Predictive Models That Transport. *AISTATS 2019 - 22nd Int Conf Artif Intell Stat* 2018;

For peer review only

Additional files

Additional file 1

File name: Additional file 1

File format: .docx

Title: Model development

Description: Detailed description of the methods used to develop the models.

Additional file 2

File name: Additional file 2

File format: .docx

Title: Comparative scoring systems

Description: Description of the components, weightings and details of other scoring systems implemented in this study.

Additional file 3

File name: Additional file 3

File format: .docx

Title: Additional results

Description: Further results of the performance of the models.

For peer review only

Tables Legends

Table 1. Details of the ICU admissions and the subgroups of post-ICU admissions included in the development and validation cohorts. Continuous (numeric) variables are displayed as *median [interquartile range] (mean)*, and count variables are displayed as *counts (%)*. “OUH” refers to admissions from the Oxford University Hospitals NHS Foundation Trust’s two adult ICUs. “RBH” refers to admissions from the Royal Berkshire NHS Foundation Trust’s single adult ICU. “LoS” denotes length of stay.

Table 2. Performance of the first model (RS_1) on both development and validation datasets for the combined outcome (or adverse event) of in-hospital death or re-admission to ICU at any point after discharge from ICU. Performance for each event separately are also displayed. Cox’s calibration regression: for a good calibration, α should be close to 0, and β should be close to 1. AUROC denotes area under the receiver operating characteristic curve, shown with mean (standard deviation, SD).

Table 3. Area under the receiver operating characteristic curve (AUROC), standard deviation and corresponding 95% confidence interval (CI) for the developed risk scoring index (RSI) and other competitive early warning score (EWS) systems, using adverse event (re-admission to ICU or in-hospital death) within 24h of an observation set as the (compound) outcome. Results are shown for each outcome independently using both development and validation datasets.

Figure Legends

Figure 1. Representation of a set of the variables acquired for an example patient included in our study pre (represented with circles) and post discharge from the ICU. Variables include vital signs (dark grey), laboratory tests (lighter grey) and interventions/treatments (white) performed. The grey vertical line marks the patient’s ICU discharge timepoint. A more detailed electronic patient record is “generated” during the patient’s ICU stay. We also note the different frequency of measurement of the vital signs in the ICU from that on the ward.

Figure 2. Performance of the developed risk scoring system (RSI) and the other competitive scoring systems considered for predicting an adverse event (either re-admission to ICU or in-hospital death) within 12, 24, 36, 48, and 72h of a vital-sign

For peer review only

observation set. The left-hand panel shows the performance on the development dataset, the right-hand panel shows the performance on the validation dataset. The performance is represented with the mean AUROC (area under the receiver operating characteristic curve) values.

For peer review only

Table 1.

	Development (OUH)	Validation (RBH)
**ICU admissions included, n**	**8,162**	**3,421**
Age, years	62 [47 – 72] (59)	65 [51 – 75] (62)
Females sex, n	3,381 (41.4%)	1,480 (43.3%)
LoS in ICU, days	1.9 [1.0 – 4.0] (4.2)	2.0 [1.0 – 4.5] (4.2)
Outcome:		
ICU re-admission, n	543 (6.7%)	154 (4.5%)
Time to ICU re-admission, hours	92 [37 – 205] (225)	101 [43 – 283] (340)
In-hospital death, n	462 (5.7%)	218 (6.4%)
Time to in-hospital death, hours	296 [84 – 781] (701)	204 [66 – 600] (540)
Adverse event, n	902 (11.1%)	329 (9.6%)
**Post-ICU admissions included, n**	**273**	**136**
Age, year	64 [51 – 72] (60)	66 [55 – 73] (63)
Females sex, n	85 (31.1)	61 (45)
LoS in ICU, days	1.9 [1.0 – 3.9] (3.5)	1.3 [0.9 – 3.0] (3.4)
Outcome:		
ICU re-admission, n	18 (6.6%)	9 (6.6%)
Time to ICU re-admission, hours	162 [73 – 220] (215)	108 [72 – 202] (153)
In-hospital death, n	6 (2.2%)	4 (2.9%)
Time to in-hospital death, hours	216 [66 – 514] (356)	322 [244 – 365] (288)
Adverse event, n	23 (8.4%)	11 (8.1%)

w only

Table 2.

	Development dataset		
	**Adverse event**	**In-hospital death**	**ICU re-admission**
Hosmer-Lemeshow statistic	50.81	339.07	224.94
Brier score	0.087	0.050	0.065
Cox's calibration:			
α	-0.009	-0.037	0.013
β	0.988	1.104	0.472
AUROC (SD)	0.782 (0.022)	0.843 (0.022)	0.716 (0.022)
	**Validation dataset**		
Hosmer-Lemeshow statistic	57.35	175.68	275.08
Brier score	0.081	0.058	0.058
Cox's calibration:			
α	0.017	-0.012	0.033
β	0.627	0.475	0.268
AUROC (SD)	0.723 (0.014)	0.780 (0.017)	0.623 (0.019)

For peer review only

Table 3.

	Development dataset		
	Adverse event	In-hospital death	ICU re-admission
MEWS	0.749 (0.022) [0.734 to 0.764]	0.752 (0.055) [0.717 to 0.789]	0.747 (0.021) [0.733 to 0.761]
SEWS	0.754 (0.019) [0.741 to 0.765]	0.785 (0.050) [0.753 to 0.819]	0.744 (0.022) [0.730 to 0.759]
NEWS	0.757 (0.020) [0.742 to 0.771]	0.889 (0.030) [0.867 to 0.912]	0.737 (0.022) [0.721 to 0.753]
CEWS	0.761 (0.021) [0.748 to 0.775]	0.887 (0.029) [0.866 to 0.908]	0.740 (0.022) [0.727 to 0.754]
RS_2	0.767 (0.019) [0.755 to 0.780]	0.859 (0.041) [0.836 to 0.889]	0.745 (0.020) [0.732 to 0.758]
**RSI**	**0.782 (0.018) [0.770 to 0.794]**	**0.931 (0.007) [0.926 to 0.936]**	**0.753 (0.017) [0.743 to 0.764]**
	**Validation dataset**		
MEWS	0.632 (0.032) [0.610 to 0.655]	0.697 (0.054) [0.661 to 0.733]	0.632 (0.033) [0.610 to 0.655]
SEWS	0.678 (0.037) [0.653 to 0.703]	0.678 (0.057) [0.642 to 0.717]	0.681 (0.038) [0.657 to 0.706]
NEWS	0.653 (0.044) [0.621 to 0.683]	0.675 (0.063) [0.630 to 0.719]	0.649 (0.043) [0.622 to 0.679]
CEWS	0.672 (0.036) [0.648 to 0.695]	0.685 (0.061) [0.643 to 0.728]	0.670 (0.036) [0.645 to 0.596]
RS_2	0.693 (0.040) [0.669 to 0.718]	0.681 (0.059) [0.644 to 0.722]	0.693 (0.041) [0.666 to 0.721]
**RSI**	**0.724 (0.027) [0.704 to 0.741]**	**0.725 (0.045) [0.694 to 0.757]**	**0.722 (0.028) [0.702 to 0.741]**

For peer review only

For peer review only

For peer review only

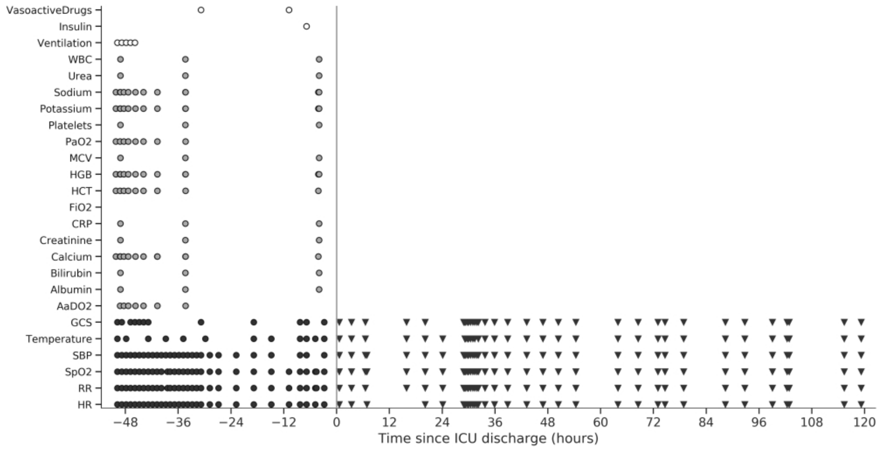

Figure 1: Representation of a set of the variables acquired for an example patient included in our study pre (represented with circles) and post discharge from the ICU. Variables include vital signs (dark grey), laboratory tests (lighter grey) and interventions/treatments (white) performed. The grey vertical line marks the patient’s ICU discharge timepoint. A more detailed electronic patient record is “generated” during the patient’s ICU stay. We also note the different frequency of measurement of the vital signs in the ICU from that on the ward.

966x515mm (138 x 138 DPI)

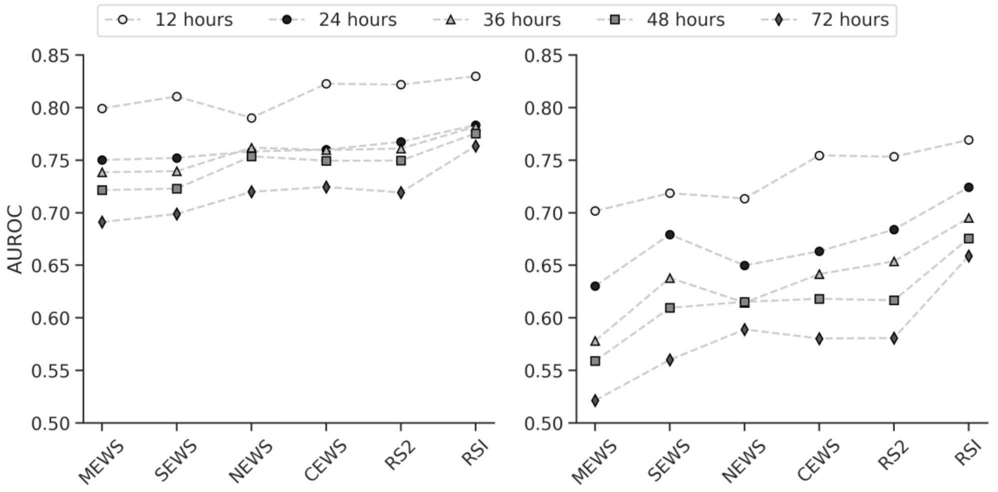

Figure 2: Performance of the developed risk scoring system (RSI) and the other competitive scoring systems considered for predicting an adverse event (either re-admission to ICU or in-hospital death) within 12, 24, 36, 48, and 72h of a vital-sign observation set. The left-hand panel shows the performance on the development dataset, the right-hand panel shows the performance on the validation dataset. The performance is represented with the mean AUROC (area under the receiver operating characteristic curve) values

662x331mm (138 x 138 DPI)

Additional file 1 – Model development

We describe in more detail the methods used to develop the model in this section.

Predictors

Our proposed approach uses a conceptual model in which we estimate the risk of a patient experiencing an adverse event immediately after discharge from ICU. This risk score (RS_1) is estimated from variables recorded during their stay in the ICU. After discharge from ICU, the patient's risk of experiencing an adverse event within the next 24 hours is updated in a time-varying manner (RS_2), using those measurements of the vital signs that are routinely measured during subsequent ward care.

<u>ICU-based feature representation</u>:

To estimate the risk of a patient experiencing an adverse event immediately after discharge from ICU, we considered candidate variables that were available electronically in our databases. The final list of candidate variables included the following physiological variables:

- Heart rate (HR), measured in beats per minute
- Respiratory rate (RR), in breaths per minute
- Systolic (SBP), diastolic and mean blood pressure, in mmHg
- Temperature, in degrees Celsius
- Peripheral oxygen saturation (SpO_2), in %
- Level of consciousness, using the Glasgow Coma Scale (GCS)

The following laboratory test results were included in the list:

- Alveolar-arterial oxygen partial pressure gradient ($AaDO_2$), in kPa
- Albumin, in g/L
- Bilirubin (total), in μmol/L
- Calcium (adjusted), in mmol/L
- Creatinine, in mmol/L
- C-reactive protein (CRP), in mg/L
- Haematocrit (HCT), in %
- Haemoglobin (HGB), in g/dL
- Lactate, in mmol/L
- Mean corpuscular volume (MCV), in fL
- Ratio of partial pressure of oxygen : fraction of inspired oxygen (PaO_2/FiO_2), in kPa
- pH
- Platelet count, in 10^9 cells/L

For peer review only

- Potassium, in mmol/L
- Sodium, in mmol/L
- Urea, measured in mmol/L
- Urine output, in mL
- White blood cell count (WBC), in 10^9 cells/L

The list also included demographic and administrative-based variables, such as age at admission, gender, number of hours between hospital admission and ICU admission, and the Index of Multiple Deprivation score (1), derived for patients with a valid postcode. We included variables related to procedures and treatments, such as the number of vasoactive drugs administered, total fluid balance, administration of insulin, enteral and parenteral nutrition feeding, and the use of mechanical ventilation, tracheostomy and central venous catheters.

To determine the risk of future compound outcome after discharge from the ICU, we derived several features from all candidate variables acquired during the patients' stay in the ICU. These features are based on the extremes of the variables considered. We generated maximum, minimum and variability (as given by the standard deviation) features for the physiological variables and laboratory test results from different periods of the ICU stay (including the first 24 hours of the patient in the ICU, their last 72, 48, and 24 hours, and/or their entire ICU stay). Additional dichotomous variables were generated if the values were in the upper or lower 5th percentile of the observed corresponding ranges, in order to account for potential non-linear associations of the variables with the adverse outcomes. Procedure and treatment variables were converted to dichotomous features for indicating whether a given procedure was performed or not, or whether a given medication was administered or not, over the entire ICU stay or in the last 24 hours of the ICU stay. This procedure resulted in 161 candidate features (including features from demographic information) for building a prediction model.

<u>Post-ICU feature representation</u>:

All vital-sign observations performed after discharge from ICU, as part of routine patient monitoring on acute care wards and collected prospectively for this study, were considered for analysis. Each set of vital signs include heart rate, systolic blood pressure, respiratory rate, body temperature, neurological status assessment using the Alert-Verbal-Painful-Unresponsive (AVPU) scale, peripheral oxygen saturation from pulse oximetry (SpO_2), a record of whether the patient was receiving supplemental oxygen at the time of the SpO_2 measurement, and the date and time of the observation. Vital-sign measurements are typically recorded every 4 or 6 hours throughout the patient's stay on the ward. At each measurement timestamp, in case of an incomplete vital-sign observation set, we used the most recent value of each variable (i.e., by carrying forward the last measurement).

For peer review only

Pre-processing and missing data

We identified obvious deviations from expected distributions and ranges of the data features using frequency graphs for all numerical and dichotomous features. Possible physiological ranges for the numerical features were defined according to clinical review and expert panel expertise, and values outside these ranges were not included in the analyses. Missing values were imputed with median and mode values from the feature distributions of numerical and dichotomous features, respectively. While other methods were considered, such as multiple imputation, the use of the median and mode was simpler and was deemed sufficient for this work in which the amount of missing data was low.

All numerical features were then standardised using a zero-mean, unit-variance transformation (i.e., using the mean and standard deviations from the feature distributions). This prevents features with relatively small changes in their units of measurement (such as temperature) from being dominated by features with relatively large changes (such as blood pressure), thus ensuring that all features have similar dynamic ranges.

For both imputation and normalisation, the parameters' values (for the median, mode, mean and standard deviation) found for the development dataset were used.

ICU-based model (RS_1)

We used all candidate features derived from the variables acquired during the patients' stay in the ICU to build the first model. A L1-regularised logistic regression using the "glmnet" package in Matlab (Mathworks, Natick, Massachusetts, USA) for predicting the compound outcome (in-hospital death or re-admission to ICU). L1-regularisation shrinks the less important features' coefficients to zero thus effectively removing those features that are deemed to be uninformative to the outcome variable. We estimated the regularisation parameter using LASSO (Least Absolute Shrinkage and Selection Operator) regression in a 5-fold cross-validation on the development set.

Vital-sign based model (RS_2)

For the second scoring system, RS_2, we used the vital signs (heart rate, respiratory rate, systolic blood pressure, oxygen saturation, and temperature) recorded in hospital after discharge from the ICU. For this model, rather than a *supervised learning approach*, we considered a novelty detection method (or one-class classification method), which does not require an outcome variable for development (2). This is useful where event rates are extremely low, or total sample sizes are constrained. Similar approaches have been used in our previous research (3–6) and underpin a commercial, clinically-used system, described in (7).

For peer review only

We considered the construction of a multivariate, (possibly) multimodal model of normality, based on the vital-sign observation sets recorded during the 24 hours that preceded discharge home. If the patient had not died, was not re-admitted to the ICU and was not discharged by the 14th post-ICU day, we used recorded data on that day. These observation sets are thus assumed to contain the vital-sign values from the most stable period of the patient's hospital stay, because these data were acquired when patients are at their most "stable" after discharge from ICU. This set of "normal" data contains $N = 1{,}082$ 5-dimensional vital-sign vectors, $\mathbf{X} = \{\mathbf{x_1},\ldots,\mathbf{x_N}\} \in \mathbb{R}^5$, which were subsequently used for the construction of our model of normality.

A kernel density estimate, or KDE (2) was used to estimate the probability density function (pdf) of the set of five vital signs. This is in a non-parametric technique where no *a priori* assumptions about the form of the underlying probability distribution are made. Our notation follows that reported previously (5, 6). The data pdf $p(\mathbf{x})$ was estimated using the $N = 1{,}082$ set of observations as shown in (SM1-1).

$$p(\mathbf{x}|\mathbf{x}_i,\sigma) = \frac{1}{N(2\pi)^{D/2}\sigma^D}\sum_{i=1}^{N}\exp\left(\frac{-|\mathbf{x}-\mathbf{x}_i|^2}{2\sigma^2}\right) \qquad \text{(SM1-1)}$$

This is a weighted sum of Gaussian kernels, each with identical variance σ^2 (i.e., isotropic kernels), centred on the observation sets $\mathbf{x_1},\ldots,\mathbf{x_N}$.

The nearest-neighbour method was used to estimate the variance. Briefly, this method involves determining the squared Euclidean distance (Δ) for each observation i to its 10 nearest neighbours (*NNs*), as shown in (SM1-2).

$$\Delta_i = \frac{1}{10}\sum_{j \in \{NNs\}}\|\mathbf{x}_i - \mathbf{x}_j\| \qquad \text{(SM1-2)}$$

This quantity, Δ, is then used to estimate the variance σ^2, as shown in (SM1-3).

$$\sigma = \frac{1}{N}\sum_{i=1}^{N}\Delta_i \qquad \text{(SM1-3)}$$

Estimation of the underlying pdf of normal vital-sign data provides a means of quantifying the degree to which a given set of observations is abnormal. The likelihood $p(\mathbf{x}|\mathbf{x}_i,\sigma)$, a measure which represents the probability of observing a set of measurements given a pdf, can be used for this purpose. Thus, we define the novelty score as in (SM1-4).

$$z(\mathbf{x}) = -\log p(\mathbf{x}|\mathbf{x}_i,\sigma) \qquad \text{(SM1-4)}$$

For normal data, the new observation $\mathbf{x}$ will be similar to previously-seen normal observations $\mathbf{x}_i$, and so the likelihood will be high. Consequently, the negative log-likelihood will be low, and so the novelty score $z(\mathbf{x})$ will be low. Conversely, for abnormal data, the data will be dissimilar and the likelihood will be low, and consequently the novelty score $z(\mathbf{x})$ will be high. This procedure ensures that the novelty score can be interpreted as most early warning scores (EWS), in that high scores are associated with higher abnormality of the vital signs.

For peer review only

In short, this model allows the generation of a score corresponding to an assessment of whether a vital-sign set should be deemed "stable" with respect to the development dataset (i.e., the observation sets used for training the model). By extension, it further allows the model to estimate the degree of abnormality of a given vital-sign observation set.

Risk Score Index (RSI)

An overall risk score, the risk score index (RSI), was subsequently determined using a simple time-dependent linear combination of the two constituent risk scores, such that:

$$\mathrm{RSI} = \beta\left[\left(1 - \frac{t}{Tmax}\right)\mathrm{RS}_1\right] + \left[\left(\frac{t}{Tmax}\right)\mathrm{RS}_2\right] \quad \text{(Equation 1)}$$

where t corresponds to the elapsed time (in hours) since the patient was discharged from ICU and has a maximum value of $Tmax$ hours. The time-dependent weighting function allows the contribution of RS_1 to gradually decrease with time after discharge from the ICU. The patient's risk of future adverse events becomes increasingly determined by the values of the vital-sign measurements taken on the acute ward (i.e., RS_2). The parameter β is used to adjust the weight of RS_1 with respect to the time since discharge from ICU, and the parameter $Tmax$ corresponds to the time at which RS_1 no longer affects RSI; i.e., when $t \geq Tmax$, then $\mathrm{RSI} = RS_2$. The parameters β and $Tmax$ were determined using a grid-search algorithm with 3-fold cross-validation, by defining a grid of possible values of $\beta = [0.1,\ 0.2,\ 0.3, \ldots, 200]$ and $Tmax = [12, 24, 36, \ldots, 336]$. We selected the values of β and $Tmax$ that corresponded to the highest mean area under the curve of the receiver operating characteristic (AUROC) value across all cross-validation folds, using the compound outcome of in-hospital death or ICU re-admission within the next 24 hours of a vital-sign observation.

During the development of RSI, values of $\beta = 100.2$ and $Tmax = 96$ hours, for the linear time-dependent weighting function (see Equation 1), were obtained and used to calculate the risk scoring index.

Model validation and statistical analysis

To validate the first model, RS_1, its discrimination and calibration were analysed. Discrimination is defined as the ability of the model to separate non-event patients from patients who had an adverse event after ICU discharge, and was assessed using the AUROC metric. Calibration assesses the degree of correspondence between the estimated probability of occurrence of an adverse event and that

For peer review only

actually observed. This was tested using a goodness of fit test, the Hosmer-Lemeshow "C" statistic (8). When the predicted probability of adverse events of the prognostic model differs significantly from the observed pattern, the calibration ability of the model is deemed to be poor. As the Hosmer-Lemeshow test does not measure the magnitude of miscalibration and is sensitive to sample size (9), calibration was also assessed with the Brier score and Cox's calibration regression. The latter assesses the degree of miscalibration by fitting a logistic regression of observed survival to the predicted log-odds of survival from the risk model (10). The performance of the first model was examined both for the compound outcome and each adverse event (in-hospital death and ICU re-admission) individually. The ability of the first model to predict future adverse events at increasing intervals from ICU discharge was also examined by calculating the AUROC for future events by day after discharge (up to 120 days).

The final model, RSI, was validated using the AUROC for the derived outcome of in-hospital death or ICU re-admission within the next *N* hours of a vital-sign observation/measurement performed after ICU discharge, in line with previous studies for evaluating EWS systems (11, 12). We evaluated the model for different values of *N*, with $N = [12, 24, 36, 48, 72]$ hours. We note that in this case the AUROC represents how well the scoring system RSI discriminates between observation sets followed by an adverse event and those with no subsequent adverse outcome within the next *N* hours. Therefore, the unit of analysis is a vital-sign set rather than a patient-admission, as performed for the validation of the first model.

We also considered each individual adverse event separately. To further understand the feasibility of implementation of the risk scoring systems in this setting, the burden of observation sets "triggered" for every correctly identified observation followed by an adverse event within 24 hours by the risk scoring system was also evaluated.

We report the cross-validation results using the development dataset. This gives an estimate of how our models perform on a random set of samples from the OUH Trust that were not used for developing the model. We also report the external validation results using data from the RBH Trust. Confidence intervals were estimated using bootstrap confidence intervals via percentiles with 500 samples (13).

References

1. GOV.UK (National Statistics): English indices of deprivation 2015. *Accessed from https//www.gov.uk/government/statistics/english-indices-of-deprivation-2015; Accessed January 2018*

2. Pimentel MAF, Clifton DA, Clifton L, et al.: A review of novelty detection. *Signal Processing* 2014; 99:215–249

3. Watkinson PJ, Barber VS, Price JD, et al.: A randomised controlled trial of the effect of continuous electronic physiological monitoring on the adverse event

For peer review only

rate in high risk medical and surgical patients. *Anaesthesia* 2006;

4. Clifton DA, Wong D, Clifton L, et al.: A large-scale clinical validation of an integrated monitoring system in the Emergency Department. *IEEE J Biomed Heal Informatics* 2013;

5. Johnson AEW, Burgess J, Pimentel MAF, et al.: Physiological trajectory of patients pre and post ICU discharge. In: 2014 36th Annual International Conference of the IEEE Engineering in Medicine and Biology Society. IEEE; 2014. p. 3160–3163.

6. Pimentel MAF, Clifton DA, Clifton L, et al.: Modelling physiological deterioration in post-operative patient vital-sign data. *Med Biol Eng Comput* 2013;

7. Orphanidou C, Clifton D, Shahab Khan, et al.: Telemetry-based vital sign monitoring for ambulatory hospital patients. In: 2009 Annual International Conference of the IEEE Engineering in Medicine and Biology Society. IEEE; 2009. p. 4650–4653.

8. Lemeshow S, Hosmer DW: A review of goodness of fit statistics for use in the development of logistic regression models. *Am J Epidemiol* 1982;

9. Kramer AA, Zimmerman JE: Assessing the calibration of mortality benchmarks in critical care: The Hosmer-Lemeshow test revisited*. *Crit Care Med* 2007; 35:2052–2056

10. Cox DR: Two further applications of a model for binary regression. *Biometrika* 1958; 45:562–565

11. Prytherch DR, Smith GB, Schmidt PE, et al.: ViEWS-Towards a national early warning score for detecting adult inpatient deterioration. *Resuscitation* 2010; 81:932–937

12. Badriyah T, Briggs JS, Meredith P, et al.: Decision-tree early warning score (DTEWS) validates the design of the National Early Warning Score (NEWS). *Resuscitation* 2014; 85:418–423

13. DiCiccio TJ, Efron B: Bootstrap confidence intervals. *Stat Sci* 1996;

For peer review only

Additional file 2 – Comparative scoring systems

In this section, we described the details of other scoring systems used in this study and how they were implemented using the information included in datasets. We also detail the components and weightings of the individual scores, as they were implemented in this work.

The early warning score (EWS) systems evaluated in this study include the modified EWS, or MEWS (1), the standardised EWS, or SEWS (2), the National EWS, or NEWS (3, 4) and the centile-based EWS, or CEWS (5). Their components and weightings are shown in tables SM2-1, SM2-2, SM2-3 and SM2-4, respectively. The core set of variables that are used in these scoring systems comprises heart rate (measured in beats per minute), respiratory rate (in breaths per minute), systolic and diastolic blood pressure (in mmHg), body temperature (in °C), oxygen saturation (denoted "SpO_2", in %), an assessment of neurological status (using the Alert-Verbal-Painful-Unresponsive or AVPU scale), a flag for whether the patient was receiving supplementary oxygen at the time of the SpO_2 measurement (denoted "Inspired O_2"), and the age to the patient at admission to hospital. At each observation set (or measurement timestamp), in case of an incomplete vital-sign observation set, we used the most recent value of each variable. Remaining missing data were dealt with as with the proposed scoring system (see section 2 in the main manuscript).

Table SM2-1. Modified Early Warning Score (see main text for units of the variables included).

Table SM2-2. Standardised Early Warning Score (see main text for units of the variables included).

Table SM2-3. National Early Warning Score (see main text for units of the variables included).

Table SM2-4. Centile-based Early Warning Score (see main text for units of the variables included).

For peer review only

Table SM2-1.

Modified Early Warning Score (MEWS)

	Score						
Variable	3	2	1	0	1	2	3
Heart rate	≤ 39	40 – 50	51 – 59	60 – 100	101 – 110	111 – 129	≥ 130
Resp. rate		≤ 8		9 – 14	15 – 20	21 – 29	≥ 30
Temperature		≤ 35.0		35.1 – 38.0		38.1 – 39.5	≥ 39.6
Systolic BP	≤ 69	70 – 80	81 – 100	101 – 149	150 – 169	170 – 179	≥ 180
AVPU scale				A	V	P	U

Table SM2-2.

Standardised Early Warning Score (SEWS)

	Score						
Variable	3	2	1	0	1	2	3
Heart rate	≤ 29	30 – 39	40 – 49	50 – 99	100 – 109	110 – 129	≥ 130
Resp. rate	≤ 8			9 – 20	21 – 30	31 – 35	≥ 36
Temperature	≤ 33.9	34.0 – 34.9	35.0 – 35.9	36.0 – 37.9	38.0 – 38.9	≥ 39.0	
Systolic BP	≤ 69	70 – 79	80 – 99	100 – 199		≥ 200	
SpO$_2$	≤ 84	85 – 89	90 – 92	≥ 93			
AVPU scale				A	V	P	U

Table SM2-3.

National Early Warning Score (NEWS)

	Score						
Variable	3	2	1	0	1	2	3
Heart rate	≤ 40		41 – 50	51 – 90	91 – 110	111 – 130	≥ 131
Resp. rate	≤ 8		9 – 11	12 – 20		21 – 24	≥ 25
Temperature	≤ 35.0		35.1 – 36.0	36.1 – 38.0	38.1 – 39.0	≥ 39.1	
Systolic BP	≤ 90	91 – 100	101 – 110	111 – 219			≥ 220
SpO$_2$	≤ 91	92 – 93	94 – 95	≥ 96			
Inspired O$_2$		Any O$_2$		Air			
AVPU scale				A			V, P, U

Table SM2-4.

Centile-based Early Warning Score (CEWS)

	Score						
Variable	3	2	1	0	1	2	3
Heart rate	≤ 42	43 – 49	50 – 53	54 – 104	105 – 112	113 – 127	≥ 128
Resp. rate	≤ 7	8 – 10	11 – 12	13 – 21	22 – 23	24 – 28	≥ 29
Temperature	≤ 35.4		35.5 – 35.9	36.0 – 37.3	37.4 – 38.3		≥ 38.4
Systolic BP	≤ 83	84 – 90	91 – 100	101 – 157	158 – 167	168 – 184	≥ 185
SpO$_2$	≤ 84	85 – 90	91 – 93	≥ 94			
Inspired O$_2$		Any O$_2$		Air			
AVPU scale				A	V		P, U

References

1. Gardner-Thorpe J, Love N, Wrightson J, et al.: The value of Modified Early Warning Score (MEWS) in surgical in-patients: A prospective observational study. *Ann R Coll Surg Engl* 2006; 88:571–575
2. Paterson R, MacLeod DCD, Thetford D, et al.: Prediction of in-hospital mortality and

For peer review only

length of stay using an early warning scoring system: clinical audit. *Clin Med J R Coll Physicians London* 2006; 6:281–284

3. Smith GB, Prytherch DR, Meredith P, et al.: The ability of the National Early Warning Score (NEWS) to discriminate patients at risk of early cardiac arrest, unanticipated intensive care unit admission, and death. *Resuscitation* 2013; 84:465–470
4. Royal College of Physicians (RCP): National Early Warning Score (NEWS). *Stand Assess acute-illness Sev NHS* 2012; 47
5. Watkinson PJ, Pimentel MAF, Clifton DA, et al.: Manual centile-based early warning scores derived from statistical distributions of observational vital-sign data. *Resuscitation* 2018; 129:55–60

For peer review only

Additional file 3 – Additional results

In this section, we describe in more detail some of the results of the study.

Participants

Figure SM3-1 shows the application of inclusion/exclusion criteria to patient-admissions in order to derive the final cohorts in both organisations included in this study.

Figure SM3-1. Diagram showing the construction of the study cohorts from both organisations (OUH and RBH) included in the study by applying inclusion/exclusion criteria.

ICU-based model (RS_1)

The features computed from the data acquired during the patients' ICU stay that were selected for the first model are detailed in Table SM3-1. A total of 45 features were included in the model from the 161 candidate features.

Calibration plots of the model for both development and validation sets are shown in Figure SM3-2. Figure SM3-3 shows the AUROC values achieved by the first model (RS_1) for predicting the adverse event (combined outcome) within an increasingly high number of days after discharge from ICU. The curves of the AUROC values for each outcome, in-hospital death or ICU re-admission, separately, are also shown.

Table SM3-1. Features computed from information acquired during the patients' ICU stay used in the first model, and the corresponding odds ratio and 95% confidence interval (95% CI) for the odds ratio. The normalisation parameters for each feature (mean and standard deviation, SD) are also shown.

Figure SM3-2. Calibration curves for the first model (RS_1) for predicting a future adverse event after ICU discharge. The left-hand panel shows the calibration curve on the development dataset, the right-hand panel shows the calibration curve of the developed model on the validation dataset. The grey line depicts an ideal calibration, representing a perfect correspondence between the observed events and the predicted events for each decile of score.

Figure SM3-3. Performance of the first model (as given by the AUROC) for prediction of cumulative in-hospital deaths, cumulative ICU re-admissions, and cumulative adverse events up to a cut-off day after ICU discharge. The left-hand panel shows the results using

For peer review only

the development dataset, and the right-hand panel shows the performance using the validation dataset. The bottom graphs show the proportion of each event (N) over time for the three outcomes considered.

Vital-sign based model (RS_2)

For the second scoring system, we considered the construction of a multivariate, (possibly) multimodal model of normality, based on the vital-sign observation sets recorded during the 24 hours that preceded discharge home or during the last post-ICU day with recorded data (which corresponds to the 14th day) if the patient had not been discharged, died or re-admitted to the ICU. These observation sets contain the vital signs from the most stable period of the patient's stay, because these data were acquired when patients are at their most "normal" state after discharge from ICU. This set of "normal" data contains $N = 1{,}082$ 5-dimensional vital-sign vectors. Table SM3-2 gives the corresponding means and standard deviations of this subset of data, used to train the model.

Table SM3-2. Vital signs' mean and standard deviation (SD) values for observations recorded in during the most "stable" period considered.

For peer review only

Figure SM3-1.

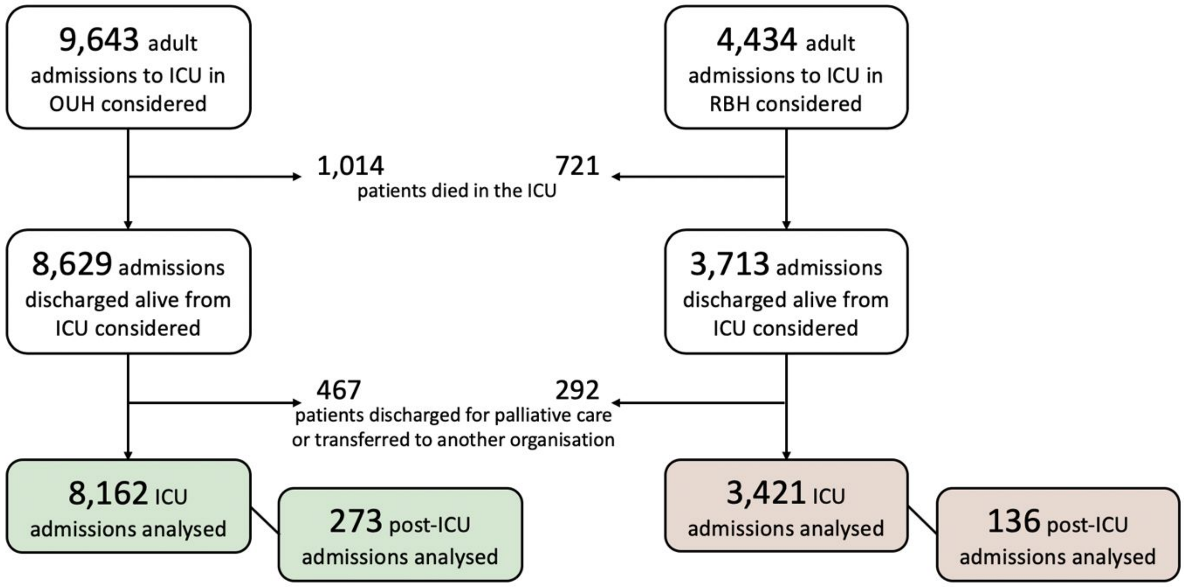

For peer review only

Figure SM3-2.

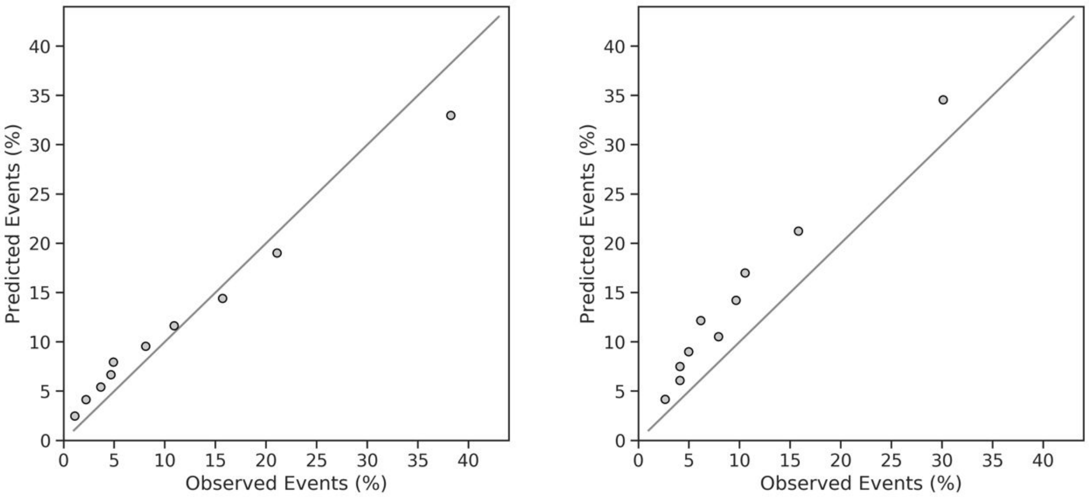

peer review only

Figure SM3-3.

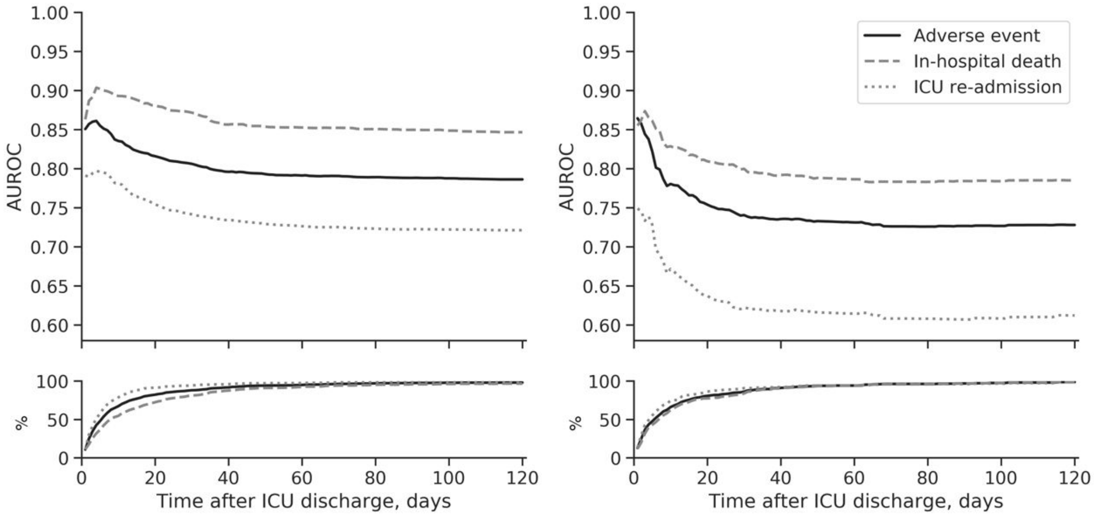

For peer review only

Table SM3-1.

Feature description	Mean	(SD)	Odds ratio	95% CI
**Demographic and administrative information** (3 features)				
Age at admission to ICU	59	(17)	1.305	1.196 to 1.425
Time spend in the ICU, in days	4.3	(19.5)	1.134	1.072 to 1.199
Time from acute hospital admission and ICU admission, in days	4.2	(7.4)	1.038	0.964 to 1.119
**Vital signs related** (13 features)				
Last ventricular heart rate	86	(17)	1.135	1.024 to 1.259
Highest respiratory rate in the last 72 hours	26	(7)	1.129	1.033 to 1.235
Lowest ventricular heart rate in the last 72 hours	70	(16)	1.056	0.927 to 1.202
Highest ventricular heart rate in the first 24 hours	105	(22)	1.039	0.944 to 1.143
Last respiratory rate	18	(5)	1.026	0.943 to 1.116
Lowest ventricular heart rate in the first 24 hours	72	(17)	1.023	0.901 to 1.161
Highest SpO_2 recorded using pulse oximetry in the last 72 hours	100	(1)	1.016	0.944 to 1.094
Lowest respiratory rate in the first 24 hours	12	(3)	1.002	0.925 to 1.085
Lowest temperature in the first 24 hours	37.0	(0.7)	0.984	0.906 to 1.068
Highest temperature in the first 24 hours	35.5	(0.8)	0.981	0.889 to 1.083
Last mean arterial blood pressure	91	(16)	0.921	0.853 to 0.995
Lowest temperature in the last 72 hours	35.5	(0.7)	0.917	0.831 to 1.011
Last total GCS recorded	14	(1)	0.854	0.805 to 0.906
**Laboratory test results related** (24 features)				
Last plasma urea	9.1	(7.1)	1.313	1.186 to 1.453
Last plasma sodium	139	(4)	1.152	1.073 to 1.237
Highest INR in the first 24 hours	1.23	(0.48)	1.063	0.982 to 1.151
Last plasma bilirubin	12.8	(14.8)	1.052	0.984 to 1.124
Highest pH in the first 24 hours	7.43	(0.06)	1.051	0.971 to 1.138
Highest C-reactive protein in the first 24 hours	91	(55)	1.050	0.939 to 1.173
Last plasma lactate	1.16	(0.71)	1.028	0.957 to 1.103
Last INR	1.13	(0.35)	1.027	0.951 to 1.111
Last C-reactive protein	89	(54)	1.009	0.904 to 1.126
Highest platelet count in the first 24 hours	200	(109)	1.006	0.929 to 1.089
Last plasma sodium was in the extremes of the development set range (> or <)	-	-	1.003	0.831 to 1.211
Last plasma creatinine	116	(130)	0.993	0.735 to 1.342
The variability of $AaDO_2$ in the last 72 hours	6.1	(5.1)	0.990	0.908 to 1.080
Highest weight in the first 24 hours	77	(20)	0.989	0.909 to 1.076
Last haematocrit	32	(5)	0.989	0.881 to 1.110
Highest creatinine in the last 24 hours	124	(142)	0.975	0.744 to 1.278

For peer review only

Lowest haemoglobin in the first 24 hours	9.6	(2.0)	0.963	0.864 to 1.075
Highest white blood cell count in the first 24 hours	13.9	(7.0)	0.962	0.893 to 1.036
Lowest plasma potassium in the first 24 hours	3.8	(0.5)	0.951	0.879 to 1.030
Highest plasma creatinine in the last 72 hours	134	(153)	0.950	0.752 to 1.200
Total urine output in the first 24 hours	1772	(1260)	0.937	0.842 to 1.043
Last PaO_2/FiO_2 ratio	43.7	(15.8)	0.904	0.826 to 0.990
Last plasma albumin	28	(6)	0.870	0.798 to 0.949
Total urine output in the last 24 hours	2007	(1293)	0.781	0.703 to 0.867
**Procedures and treatments related (**5 features**)**				
If enteral feeding was used at any point during ICU stay	-	-	1.119	0.946 to 1.323
If a central venous catheter was present at ICU discharge	-	-	1.118	0.957 to 1.306
If an insulin infusion was used in the last 24 hours	-	-	1.055	0.894 to 1.246
If parenteral feeding was used at any point during ICU stay	-	-	1.044	0.799 to 1.363
A dysrhythmia at any point during ICU stay	-	-	1.001	0.999 to 1.003

For peer review only

Table SM3-2.

Vital sign	Mean (SD)
Heart rate, beats per min.	83 (14)
Respiratory rate, breaths per min.	17 (2)
Systolic blood pressure, mmHg	129 (19)
SpO_2, %	97 (3)
Temperature, C	36.3 (0.5)

For peer review only

Reporting checklist for prediction model development/validation.

Based on the TRIPOD guidelines.

Instructions to authors

Complete this checklist by entering the page numbers from your manuscript where readers will find each of the items listed below.

Your article may not currently address all the items on the checklist. Please modify your text to include the missing information. If you are certain that an item does not apply, please write "n/a" and provide a short explanation.

Upload your completed checklist as an extra file when you submit to a journal.

In your methods section, say that you used the TRIPODreporting guidelines, and cite them as:

Collins GS, Reitsma JB, Altman DG, Moons KG. Transparent reporting of a multivariable prediction model for individual prognosis or diagnosis (TRIPOD): The TRIPOD statement.

		Reporting Item	Page Number
**Title**			
	#1	Identify the study as developing and / or validating a multivariable prediction model, the target population, and the outcome to be predicted.	1
**Abstract**			

For peer review only

	#2	Provide a summary of objectives, study design, setting, participants, sample size, predictors, outcome, statistical analysis, results, and conclusions.	3
**Introduction**			
	#3a	Explain the medical context (including whether diagnostic or prognostic) and rationale for developing or validating the multivariable prediction model, including references to existing models.	4
	#3b	Specify the objectives, including whether the study describes the development or validation of the model or both.	4-8
**Methods**			
Source of data	#4a	Describe the study design or source of data (e.g., randomized trial, cohort, or registry data), separately for the development and validation data sets, if applicable.	5
Source of data	#4b	Specify the key study dates, including start of accrual; end of accrual; and, if applicable, end of follow-up.	5
Participants	#5a	Specify key elements of the study setting (e.g., primary care, secondary care, general population) including number and location of centres.	5
Participants	#5b	Describe eligibility criteria for participants.	5
Participants	#5c	Give details of treatments received, if relevant	N/A

For peer review only

Outcome	#6a	Clearly define the outcome that is predicted by the prediction model, including how and when assessed.	6
Outcome	#6b	Report any actions to blind assessment of the outcome to be predicted.	N/A
Predictors	#7a	Clearly define all predictors used in developing or validating the multivariable prediction model, including how and when they were measured	6
Predictors	#7b	Report any actions to blind assessment of predictors for the outcome and other predictors.	N/A
Sample size	#8	Explain how the study size was arrived at.	N/A
Missing data	#9	Describe how missing data were handled (e.g., complete-case analysis, single imputation, multiple imputation) with details of any imputation method.	5
Statistical analysis methods	#10a	If you are developing a prediction model describe how predictors were handled in the analyses.	7-8
Statistical analysis methods	#10b	If you are developing a prediction model, specify type of model, all model-building procedures (including any predictor selection), and method for internal validation.	7-8
Statistical analysis methods	#10c	If you are validating a prediction model, describe how the predictions were calculated.	7-8
Statistical analysis methods	#10d	Specify all measures used to assess model performance and, if relevant, to compare multiple models.	7-8

For peer review only

Statistical analysis methods	#10e	If you are validating a prediction model, describe any model updating (e.g., recalibration) arising from the validation, if done	7-8
Risk groups	#11	Provide details on how risk groups were created, if done.	N/A
Development vs. validation	#12	For validation, identify any differences from the development data in setting, eligibility criteria, outcome, and predictors.	5

Results

Participants	#13a	Describe the flow of participants through the study, including the number of participants with and without the outcome and, if applicable, a summary of the follow-up time. A diagram may be helpful.	5
Participants	#13b	Describe the characteristics of the participants (basic demographics, clinical features, available predictors), including the number of participants with missing data for predictors and outcome.	Table 1
Participants	#13c	For validation, show a comparison with the development data of the distribution of important variables (demographics, predictors and outcome).	Table 1
Model development	#14a	If developing a model, specify the number of participants and outcome events in each analysis.	8
Model development	#14b	If developing a model, report the unadjusted association, if calculated between each candidate predictor and outcome.	9

For peer review only

Model specification	#15a	If developing a model, present the full prediction model to allow predictions for individuals (i.e., all regression coefficients, and model intercept or baseline survival at a given time point).	9
Model specification	#15b	If developing a prediction model, explain how to the use it.	N/A
Model performance	#16	Report performance measures (with CIs) for the prediction model.	9
Model-updating	#17	If validating a model, report the results from any model updating, if done (i.e., model specification, model performance).	N/A
**Discussion**			
Limitations	#18	Discuss any limitations of the study (such as nonrepresentative sample, few events per predictor, missing data).	11
Interpretation	#19a	For validation, discuss the results with reference to performance in the development data, and any other validation data	9-11
Interpretation	#19b	Give an overall interpretation of the results, considering objectives, limitations, results from similar studies, and other relevant evidence.	9-11
Implications	#20	Discuss the potential clinical use of the model and implications for future research	12

For peer review only

Other information

Supplementary information	#21	Provide information about the availability of supplementary resources, such as study protocol, Web calculator, and data sets.	19
Funding	#22	Give the source of funding and the role of the funders for the present study.	13

The TRIPOD checklist is distributed under the terms of the Creative Commons Attribution License CC-BY. This checklist was completed on 11. April 2023 using https://www.goodreports.org/, a tool made by the EQUATOR Network in collaboration with Penelope.ai

For peer review only

Development of an enhanced scoring system to predict ICU re-admission or in-hospital death within 24h using routine patient data from two NHS Foundation Trusts

Journal:	*BMJ Open*
Manuscript ID	bmjopen-2023-074604.R2
Article Type:	Original research
Date Submitted by the Author:	12-Feb-2024
Complete List of Authors:	Pimentel, Marco; University of Oxford, Department of Engineering Science<br>Johnson, Alistair; Massachusetts Institute of Technology, Institute of Medical Engineering & Science<br>Darbyshire, Julie; Oxford University, Department of Primary Care Health Sciences<br>Tarassenko, Lionel; University of Pittsburgh, Engineering Science<br>Clifton, David A.; Univ Oxford, Department of Engineering Science<br>Walden, Andrew; Royal Berkshire NHS Foundation Trust<br>Rechner, Ian; Royal Berkshire NHS Foundation Trust<br>Watkinson, Peter; University of Oxford, Nuffield Department of Clinical Neurosciences<br>Young, Duncan; Oxford University, Nuffield Department of Clinical Neurosciences
**Primary Subject Heading**:	Intensive care
Secondary Subject Heading:	Health informatics
Keywords:	Clinical Decision-Making, Health informatics < BIOTECHNOLOGY & BIOINFORMATICS, Adult intensive & critical care < INTENSIVE & CRITICAL CARE, STATISTICS & RESEARCH METHODS, CLINICAL PHYSIOLOGY

SCHOLARONE™ Manuscripts

I, the Submitting Author has the right to grant and does grant on behalf of all authors of the Work (as defined in the below author licence), an exclusive licence and/or a non-exclusive licence for contributions from authors who are: i) UK Crown employees; ii) where BMJ has agreed a CC-BY licence shall apply, and/or iii) in accordance with the terms applicable for US Federal Government officers or employees acting as part of their official duties; on a worldwide, perpetual, irrevocable, royalty-free basis to BMJ Publishing Group Ltd ("BMJ") its licensees and where the relevant Journal is co-owned by BMJ to the co-owners of the Journal, to publish the Work in this journal and any other BMJ products and to exploit all rights, as set out in our licence.

The Submitting Author accepts and understands that any supply made under these terms is made by BMJ to the Submitting Author unless you are acting as an employee on behalf of your employer or a postgraduate student of an affiliated institution which is paying any applicable article publishing charge ("APC") for Open Access articles. Where the Submitting Author wishes to make the Work available on an Open Access basis (and intends to pay the relevant APC), the terms of reuse of such Open Access shall be governed by a Creative Commons licence – details of these licences and which Creative Commons licence will apply to this Work are set out in our licence referred to above.

Other than as permitted in any relevant BMJ Author's Self Archiving Policies, I confirm this Work has not been accepted for publication elsewhere, is not being considered for publication elsewhere and does not duplicate material already published. I confirm all authors consent to publication of this Work and authorise the granting of this licence.

For peer review only

Title page

Development of an enhanced scoring system to predict ICU re-admission or in-hospital death within 24h using routine patient data from two NHS Foundation Trusts

Authors:

Marco AF Pimentel, Institute of Biomedical Engineering, Department of Engineering Science, University of Oxford, Oxford, UK

Alistair Johnson, Institute of Medical Engineering & Science, Massachusetts Institute of Technology, Cambridge, Massachusetts, USA.

Julie L Darbyshire, Nuffield Department of Clinical Neurosciences, University of Oxford, Oxford, UK

Lionel Tarassenko, Institute of Biomedical Engineering, Department of Engineering Science, University of Oxford, Oxford UK

David A Clifton, Institute of Biomedical Engineering, Department of Engineering Science, University of Oxford, Oxford UK

Andrew Walden, Intensive Care Unit, Royal Berkshire NHS Foundation Trust, Reading, UK

Ian Rechner, Intensive Care Unit, Royal Berkshire NHS Foundation Trust, Reading, UK

Peter J Watkinson, Nuffield Department of Clinical Neurosciences, University of Oxford, Oxford, UK

J Duncan Young, Nuffield Department of Clinical Neurosciences, University of Oxford, Oxford, UK

Corresponding author:
Julie L Darbyshire
Nuffield Department of Primary Care Health Sciences, University of Oxford
Oxford
OX2 6GG
UK
julie.darbyshire@phc.ox.ac.uk

Running head:
Early warning scores after ICU

For peer review only

Descriptor number:
4.6

Word count:
Body: ~3,300
Abstract: 300
Tables: 3
Figures: 2

At a Glance Commentary:

Scientific Knowledge on the Subject
Early Warning Score (EWS) systems are used to identify hospitalised patients at risk of adverse events. These systems almost all use vital signs to determine this risk. Adding further data from electronic patient records may improve the performance of these systems. Patients discharged from an intensive care unit have both a comprehensive electronic patient record and a high risk of future adverse events, so they are an ideal patient group to test if additional data improves the system performance.

What This Study Adds to the Field
The study shows a scoring system incorporating data from a patient's stay in an intensive care unit has better performance than typically-used EWS systems based on vital signs alone. The score developed in this study uses a machine learning method to estimate future risk.

This article has additional files.

For peer review only

Abstract

Rationale. Intensive care units (ICUs) admit the most severely ill patients. Once these patients are discharged from the ICU to a step-down ward, they continue to have their vital signs monitored by nursing staff, with early warning score (EWS) systems being used to identify those at risk of deterioration.

Objectives. We report the development and validation of an enhanced continuous scoring system for predicting adverse events, which combines vital signs measured routinely on acute care wards (as used by most EWSs) with a risk score of a future adverse event calculated on discharge from ICU.

Design. A modified Delphi process identified candidate variables commonly available in electronic records as the basis for a 'static' score of the patient's condition immediately after discharge from the ICU. L1-regularised logistic regression was used to estimate the in-hospital risk of future adverse event. We then constructed a model of physiological normality using vital-sign data from the day of hospital discharge. This is combined with the static score and used continuously to quantify and update the patient's risk of deterioration throughout their hospital stay.

Setting: Data from two NHS Foundation Trusts (UK) were used to develop and (externally) validate the model.

Participants: A total of 12,394 vital-sign measurements were acquired from 273 patients after ICU discharge for the development set, and 4,831 from 136 patients in the validation cohort.

Results. Outcome validation of our model yielded an area under the receiver operating characteristic curve (AUROC) of 0.724 for predicting ICU re-admission or in-hospital death within 24h. It showed an improved performance with respect to other competitive risk scoring systems, including the National EWS (NEWS, 0.653).

Conclusions. We showed that a scoring system incorporating data from a patient's stay in ICU has better performance than commonly-used EWS systems based on vital signs alone.

Keywords: early warning score; intensive care; physiological deterioration; patient monitoring.

Strengths and Limitations

* Over 17,000 vital sign measurements from >400 patients included in the cohort

For peer review only - http://bmjopen.bmj.com/site/about/guidelines.xhtml

* Variable selection informed by systematic review and Delphi process prior to data extraction
* Data extraction variables limited to those available
* Performance assessed using common methology for early warning score systems
* Compound outcome (including in-hospital mortality and ICU re-admission) limits prediction of inevitable death and improves prediction of preventable deaths

For peer review only

Introduction

Patients hospitalised for acute conditions can suffer adverse events such as cardiac arrests or unplanned admission to a higher-acuity area. These events are usually preceded by changes in vital signs some hours before (1–5). Early detection of these changes might prevent some of the subsequent adverse events, so many simple Early Warning Score (EWS) systems have been developed and deployed clinically (6–8). EWS systems are now recommended to be used as part of routine care by the UK National Institute for Health and Clinical Excellence (9–11) and are widely used in other healthcare settings.

EWS systems initially used purpose-designed paper charts (12, 13). An increasing number of healthcare providers now use electronic systems (14, 15) to gather physiological data and calculate scores (16). With the increasing use of electronic patient records, there is scope to develop improved EWS systems that include many more variables than are currently used, potentially leading to increasing predictive accuracy by using more complex algorithms to calculate scores (17).

We hypothesise that an EWS system could better predict adverse events if it included variables from a patient's electronic medical record. As a proof of concept, we studied patients discharged to acute care wards from intensive care units (ICUs). These patients have a detailed electronic patient record (EPR) containing data about their ICU stay, and typically having a high rate of adverse events occurring after discharge to a ward. In addition, there is a large body of literature describing variables that are correlated with adverse events in these patients.

This study describes the development of an enhanced scoring system for predicting adverse events in patients discharged from ICUs which combines (i) routine vital signs measured on acute care wards (as used by most EWS systems) with (ii) a risk score of a future adverse event calculated on discharge from ICU. The study further validates the ability of the proposed scoring system and other published EWS systems to identify patients at risk of death or re-admission to ICU.

Materials and Methods

This study is reported in line with the TRIPOD statement (18).

Regulatory approvals

The project had UK Health Research Authority Confidentiality Advisory Group approval (ref: ECC 7-05(f)/2011), National Research Ethics Service Committee approval (ref: 11/SC/0440) for retrospective data collection and analysis, and National Research Ethics Service Committee approval (ref: 12/SC/0357) for prospective data collection and analysis.

For peer review only - http://bmjopen.bmj.com/site/about/guidelines.xhtml

Data sources

Data from two NHS Foundation Trusts (UK) were used in this study. The Oxford University Hospitals NHS Foundation Trust (OUH) has two adult general ICUs. The Royal Berkshire Hospital NHS Foundation Trust (RBH) in Reading, UK, has a single adult general ICU.

We used routinely-collected data, stored in the ICU computerised information systems (CIS). The OUH and RBH CIS contain all measurements of physiological status, and other relevant clinical information such as patient demographics; details of treatments and interventions; and laboratory test results recorded during a patient's ICU stay. An average of 655 data items are recorded daily for each patient. All the ICUs in this study use a Philips Healthcare CIS (Philips Healthcare, Eindhoven, Netherlands). In addition we used the linked datasets submitted to the UK national comparative audit for ICUs, or ICNARC CMP (19).

During the study period, neither organisation had electronic recording of vital signs for patients in acute care wards outside the ICUs. Vital-sign data were collected prospectively for recruited patients in both organisations for the first 14 days after discharge from ICU. This timeline was chosen because the average duration of hospital stay after discharge from intensive care is around ten days in the UK (https://www.icnarc.org/Our-Audit/Audits/Cmp/Reports/Summary-Statistics). The physiological data from each patient's paper observation chart were entered into an electronic database. To verify the data entry process, 55% of patients' data were double entered.

All analyses were conducted on anonymised data.

Participants

All completed adult admissions to OUH ICUs from June 2006 to December 2015 and to the RBH ICU from April 2010 to December 2015 were used to develop the ICU discharge score. Admissions were only considered when (i) patients were discharged alive from ICU and (ii) the discharge status of the patient episode (or hospital admission) was known (or recorded). We excluded patients discharged from ICU for palliative care or transferred to another organisation. Vital signs data on wards were acquired prospectively after discharge from ICU for a subset of patients who gave consent for this in OUH between April 2013 and December 2014, and in RBH from October 2013 and December 2014.

After exclusion criteria were applied, we split the database into “development” and “validation” sets by organisation (see figure 1), such that (1) we developed and evaluated the model using data from all valid admissions within the OUH group (8,162 admissions) using cross-validation methods, and (2) we performed an

For peer review only - http://bmjopen.bmj.com/site/about/guidelines.xhtml

external validation of the model using data from all valid admissions within the RBH group (3,421 admissions).

Figure 1 (below) shows the application of inclusion/exclusion criteria to patient-admissions in order to derive the final cohorts in both organisations included in this study.

Outcomes

The primary outcome was the first occurrence of either in-hospital death or re-admission to the ICU. For the evaluation of EWS systems, we considered the compound outcome of in-hospital death or ICU re-admission within the next *N* hours of a vital-sign observation, in line with previous studies (20, 21). For the primary outcome, we set *N* = *24* hours*.* We have also evaluated the systems for different values of *N* (as detailed below). Secondary outcomes were in-hospital death or re-admission to ICU, individually.

Predictors

We used a conceptual model in which we estimated the risk of a patient experiencing an adverse event at the point of discharge from ICU from variables recorded during their stay in the ICU. After discharge from ICU, the patient's risk of experiencing an adverse event within the following 24h was calculated using both ward-recorded vital signs and the risk calculated at ICU discharge.

<u>ICU-based feature representation</u>:
We used an evidence-based technique to select candidate variables to calculate the risk of deterioration at ICU discharge. We systematically reviewed of studies reporting a significant ($p < 0.05$) association between a variable recorded during an ICU stay and either in-hospital death or ICU re-admission. The resulting list of candidate variables was reviewed by a panel of five clinical experts in a modified Delphi process who added other variables they expected to be predictive of adverse events. Candidate variables were then limited to those available in our electronic databases. These were either "static" variables (mainly based on demographic information) or time-varying variables recorded repeatedly throughout the patient's stay in the ICU (see Figure 2). To determine the risk of future compound outcome after discharge from the ICU, we derived a total of 161 candidate features from all candidate variables, which were then used for building a prediction model.

<u>Post-ICU feature representation</u>:

All vital-sign observations recorded for 14 days after discharge from ICU in the post-ICU subgroup were collected manually from patient records. Each set of vital signs includes heart rate, systolic blood pressure, respiratory rate, body temperature,

For peer review only - http://bmjopen.bmj.com/site/about/guidelines.xhtml

neurological status assessment using the Alert-Verbal-Painful-Unresponsive (AVPU) scale, peripheral oxygen saturation (SpO_2), a record of whether the patient was receiving supplemental oxygen at the time of the SpO_2 measurement, and the date and time of the observation. Vital-sign measurements are typically recorded every 4 or 6h throughout the patient's stay on the ward (see Figure 2). This was not an interventional study so conventional study size calculations are not appropriate. We have previously successfully developed or validated novelty detection algorithms on samples of 150-200 patients

The final list of candidate variables and features, and procedures for pre-processing (including dealing with missing data) are further described [see Additional file 1].

Model development

To develop the risk scoring system, our approach assumes that at each vital-sign observation performed after discharge from ICU, the patient's current condition can be characterised (or represented) by a single risk estimate. Immediately after ICU discharge, the patient is assigned a risk score (RS_1) estimated from an ICU-based set of features, which is then updated using the abnormalities in their vital signs recorded during subsequent ward care (RS_2).

We therefore built the first model, RS_1, using the features derived from the variables acquired during the patients' stay in the ICU using the development set. We used a L1-regularised logistic regressor for predicting the compound outcome. For the second scoring system, RS_2, we applied a one-class classification method (22) using the vital-sign data recorded after discharge from the ICU, as described previously (23, 24). Further details of the model development are available [see Additional file 1].

An overall risk score, the risk score index (RSI), was subsequently determined using a simple time-dependent linear combination of the two constituent risk scores, such that:

$$\mathrm{RSI} = \beta\left[\left(1 - \frac{t}{Tmax}\right)\mathrm{RS}_1\right] + \left[\left(\frac{t}{Tmax}\right)\mathrm{RS}_2\right] \qquad \text{(Equation 1)}$$

where β is used to adjust the weight of RS_1 with respect to the time since discharge from ICU, t corresponds to the elapsed time (in hours) since the patient was discharged from ICU and has a maximum value of $Tmax$ hours. Further details of the model development and optimisation of the parameters are available [see Additional file 1].

For peer review only - http://bmjopen.bmj.com/site/about/guidelines.xhtml

Model validation and statistical analysis

The discrimination of the first model, RS_1, was assessed using the area under the curve of the receiver operating characteristic (AUROC) metric. Calibration was assessed using a goodness of fit test, the Hosmer-Lemeshow “C” statistic, the Brier score and Cox’s calibration regression (25–27). The performance of this first model was examined both for the compound outcome and each adverse event (in-hospital death and ICU re-admission) individually. The ability of RS_1 to predict future adverse events at increasing intervals from ICU discharge was also examined by calculating the AUROC for future events by day after (ICU) discharge (up to 120 days).

The final model (RSI) was validated using the AUROC for the compound outcome of in-hospital death or ICU re-admission within the next N hours of a vital-sign measurement recorded after ICU discharge, in line with previous studies for evaluating EWS systems (20, 21). We evaluated the model for different values of N, with $N = [12, 24, 36, 48, 72]$ hours. We note that in this case the AUROC represents how well the scoring system RSI discriminates between observation sets followed by an adverse event and those with no subsequent adverse outcome within the next N hours. Therefore, the unit of analysis is a vital-sign set rather than a patient-admission, as performed for the validation of the first model.

We also considered each individual adverse event separately. To understand better the feasibility of implementating the risk scoring systems in this setting, we also evaluated the burden of observation sets “triggered” by the risk scoring system for every correctly identified observation followed by an adverse event within 24h.

We report the cross-validation results using the development dataset. We also report the external validation results using data from the RBH Trust. Confidence intervals were estimated using bootstrap confidence intervals via percentiles, with 500 samples (28).

Comparison with published risk scoring systems

We compared the performance of our proposed risk scoring index, RSI, with that of each model individually (RS_1 and RS_2), and with that of a number of published and clinically-used EWS systems: the modified EWS, or MEWS (29), the standardised EWS, or SEWS (30), the National EWS, or NEWS (21), and our centile-based EWS, or CEWS (31). We detail the components and weightings of the individual EWS systems in Additional file 2.

Patient and Public Involvement statement

The PICRAM study (2011 – 2015) had no embedded PPI in either conceptualisation or delivery of the work.

For peer review only

Results

The application of inclusion and exclusion criteria to patient-admissions in order to derive the final cohorts in both organisations included in this study is shown in Figure SM3-1 [see Additional file 3]. A total of 8,162 admissions to ICU were included in the development dataset. Vital-sign observations were prospectively collected for 273 patients (3.3% from the 8,162 ICU admissions) after discharge from the ICU. The validation dataset included 3,421 ICU admissions, from which 136 (4.0%) had data acquired during the post-ICU period. Table 1 provides a summary of characteristics of the development and validation cohorts derived from the ICU admissions. Both cohorts were similar in terms of demographic and administrative data. The compound outcome rate was slightly higher in the development dataset than that in the validation dataset, considering either all ICU admissions or the sub-group of post-ICU admissions. We also note that the number of in-hospital deaths is higher than the number of re-admissions to ICU. In the development cohort, 12,394 vital-sign observation sets were acquired from the 273 patients after ICU discharge, and 4,831 from the 136 patients in the validation cohort.

For the first model (RS_1), which estimates the risk of future adverse events immediately after ICU discharge, 45 features from the 161 candidate features identified from the systematic review and expert opinion, were retained in the final model. These are listed Table SM3-1 [see Additional file 3]. They comprise largely measures of cardiac or respiratory physiology, renal and hepatic function, plasma electrolytes, measures of inflammation and measures of treatment intensity. Of note, artificial ventilation during the last 24h of ICU admission was associated with a lower risk of adverse outcome. This is because all the ICUs admit ventilated, elective, post-surgical admissions who have a low risk of adverse events and are discharged within 24h of extubation. Calibration plots of the model for both development and validation sets are shown in Figure SM3-2 [see Additional file 3]. Table 2 summarises the performance for the combined outcome and for either adverse event (in-hospital death or ICU re-admission) considered individually.

Table 3 shows the performance of RSI and other baseline scoring systems for predicting observation sets followed by in-hospital death or ICU re-admission within the following 24h. The proposed scoring system showed an increased discrimination ability to predict adverse events within 24h with respect to the other risk scoring systems considered in this study. Using the external validation dataset (RBH), RSI gave an AUROC of 0.724 (95% CI of 0.704-0.741), versus 0.653 (0.621-0.683) for NEWS, and 0.672 (0.648-0.695) for CEWS. Figure 3 shows the AUROC values of the risk scoring systems for predicting the compound outcome within 12, 24, 36, 48, and 72h of a vital-sign measurement. The proposed RSI system consistently shows superior discrimination for each derived outcome.

For peer review only - http://bmjopen.bmj.com/site/about/guidelines.xhtml

Discussion

There are many variants of the original EWS system (6–8), including systems designed for specific patient groups such as children (32, 33) and patients in high-dependency units (34). These systems typically use vital signs to determine the level of risk of an adverse event. Vital signs are used because they are easily-acquired variables, regularly measured in clinical practice, and have a long history of being used to track patients' progression over time. However, point estimates of the risk of future deterioration from single vital-sign measurement sets assume "normal" values that may not be appropriate for a hospital population, and ignore other data that can add to the precision and granularity of the risk estimate. The introduction of electronic recording of vital signs and electronic patient records means that the parsimony and simplicity required by paper-based systems are now less relevant.

For peer review only

Main findings and strengths

This was a proof-of-concept study for developing and validating an enhanced EWS that uses many more electronically held variables than the conventional vital signs, and which combined dynamic and static methods of risk evaluation, as typically used in other prediction disciplines such as meteorology (35) and imaging (36). We chose to study post-ICU patients because they have a detailed electronic record generated during their ICU stay, a high adverse event rate after ICU discharge, and because a significant body of literature exists on variables associated with adverse outcomes. However, the design principles we used in this study can be applied to any acute care patient group where sufficient data are available electronically.

As part of this work we developed a new scoring system for predicting in-hospital mortality and re-admission to ICU from data collected during a patient's ICU stay. A systematic review in 2013 (37) identified seven different published scoring systems that quantify the risk of mortality or re-admission at varying intervals after ICU discharge. However, only two studies verified their systems with an external (independent) validation dataset (38, 39). They obtained AUROC values similar to those found in this study.

RSI is a dynamic system that computes an updated risk estimate every time a new vital sign is recorded. It combines this dynamic risk estimate derived from the routinely-measured vital signs on the acute ward (RS_2) with a "static" risk estimate that is computed immediately after discharge from ICU using data from the ICU CIS (RS_1). As the performance of RS_1 is expected to worsen as the time from discharge from ICU increases because patients' conditions change (see Figure SM3-3), we used a time-dependent function to reduce its contribution over time, with the patient's risk score becoming increasingly determined by vital-signs based RS_2. The combined risk estimates as given by RSI improved the predictive power of our scoring system when compared with RS_2.

In this study, a compound outcome of in-hospital mortality and ICU re-admission was used as opposed to only using in-hospital mortality (20). Patients discharged from ICU with curative intent may die in hospital in spite of full, timely and appropriate care. They may also die because they do not receive the care they need in a timely fashion. Developing a model using in-hospital mortality as the sole outcome would limit the power of such a model, as appropriately-treated survivors, with a re-admission to ICU, would not contribute to the adverse events. Hence, there would be a risk that the resulting model would predict inevitable, rather than preventable, deaths, as also noted by others (40, 41). Our main goal was to develop a system that would identify patients who were likely to respond to earlier intervention of higher acuity care. Whilst ICU re-admission does not capture all the appropriately treated survivors, there is no other marker readily available in hospital electronic records to identify these patients. A similar compound outcome has been used in validation studies of EWSs for the same reason (21, 31, 42). We further note that RSI was considerably better at predicting in-hospital mortality than ICU re-admission (see Table 3). This has also been noted in other studies reporting evaluation of EWS systems (21, 42, 43), where ICU admissions are less reliably identified than in-hospital death.

Limitations

This work has a number of limitations. We studied a very specific group of patients who were at high risk of in-hospital deterioration and who had detailed records of their ICU stay before they were discharged to the ward. We could only study the variables available in the ICU electronic records; hence, some of the candidate variables identified in the systematic review could not be included in the model. In addition, as vital-sign data had to be prospectively collected from consenting patients and transcribed from paper charts, the number of patients in the study, and therefore the number of adverse events, was limited.

We assessed the performance of the combined scoring system developed using the methodology commonly used to assess that of EWS systems. This uses AUROC measures based on paired vital-sign recordings and events within fixed time periods; i.e., derived outcome of occurrence of an adverse event within (e.g.) 24h of a vital-sign measurement. Therefore, AUROC values represent the probability that any randomly-chosen observation followed within the chosen time period by in-hospital death or ICU re-admission has a higher risk score than any randomly-chosen observation not followed by an event in the same time period (44). That is, repeated measurements from the same patient were used for evaluating the performance of scoring systems, which assumes that the scores computed from each observation set for that patient are independent (which is the usual approach when evaluating EWS systems). However, this assumption may not hold in practice; i.e., a vital-sign

For peer review only

measurement at one point in time is likely to be correlated with previous measurements. This, alongside the highly imbalanced dataset (in which the outcome occurs infrequently), gives rise to AUROCs with high values that are not truly comparable with AUROCs from single predictor/single outcome algorithms where the unit of analysis is a patient-admission (45).

An external (independent) dataset was used to validate the risk scoring systems. RSI did not perform as well as expected for the combined outcome. This was primarily because the combined system over-estimated the risk of adverse events in higher-risk patients, possibly reflecting a difference in the patient population at the two hospital sites (46). The ICUs at the OUH admit tertiary referral patients not seen in RBH; hence, risk associated with these patients captured in the scoring system built with the development dataset may not have added explanatory power for patient admissions in the validation dataset.

Finally, we note that the performance of our scoring system for predicting adverse events exceeds that of previously published EWS algorithms. This should, however, be interpreted with caution, as most of these systems were developed and validated on all hospital admissions to acute care areas, and our system was developed on a very specific population.

Conclusions

Scoring systems, such as EWSs, are used to identify hospitalised patients at risk of adverse events. In this study, we developed a bipartite score based on machine learning that encompasses the patient state at the time of ICU discharge, as well as vital signs recorded on the wards at the time the risk score is calculated. We showed that a scoring system incorporating data from a patient's stay in an ICU has better performance than typically-used EWS systems based on vital signs alone.

For peer review only

Ethical Approval and Consent to participate

The project had UK Health Research Authority Confidentiality Advisory Group approval (ref: ECC 7-05(f)/2011), National Research Ethics Service Committee approval (ref: 11/SC/0440) for retrospective data collection and analysis, and National Research Ethics Service Committee approval (ref: 12/SC/0357) for prospective data collection and analysis.

Consent for publication

Not applicable.

Availability of data and materials

Fully anonymised individual participant data may be made available to researchers directly affiliated to an academic institution or charitable organisation who provide an independently-reviewed, methodologically sound proposal. The data will be available for a period starting six months after publication and ending one year later. Only data required to answer the primary question posed by the proposed research will be made available. Researchers should contact Professor Young in the first instance. Other data and outputs from the project (Health Innovation Challenge Fund ref: HICF-0510-006) may be available on request.

Competing interests

JDY, PJW, and LT report grants from Wellcome Trust/UK Department of Health. PJW and LT report grants from National Institute for Health Research, personal fees from Sensyne Health, outside the submitted work. **PJW, DAC and LT are supported by the NIHR Biomedical Research Centre Oxford. MAFP is supported by a Drayson Research Fellowship.**

Funding

This study was supported by the Department of Health and Wellcome Trust through the Health Innovation Challenge Fund (ref: HICF-0510-006). The views expressed in this publication are those of the authors and not necessarily those of the Department of Health & Social Care or Wellcome Trust.

For peer review only - http://bmjopen.bmj.com/site/about/guidelines.xhtml

Author contributions

David A Clifton: Substantial contributions to the conception and design of the work, and analysis and interpretation of data for the work. Revising this manuscript critically for important intellectual content. Final approval of the manuscript. Agreement to be accountable for all aspects of the work.

Julie L Darbyshire: Substantial contributions to the design of the work. Revising this manuscript critically for important intellectual content. Final approval of the manuscript. Agreement to be accountable for all aspects of the work.

Alistair Johnson: Substantial contributions to the acquisition and analysis of data for the work. Revising this manuscript critically for important intellectual content. Final approval of the manuscript. Agreement to be accountable for all aspects of the work.

Marco A F Pimentel: Substantial contributions to the acquisition, analysis and interpretation of data for the work. Revising this manuscript critically for important intellectual content. Final approval of the manuscript. Agreement to be accountable for all aspects of the work.

Ian Rechner: Substantial contributions to the acquisition of data for the work. Revising this manuscript critically for important intellectual content. Final approval of the manuscript. Agreement to be accountable for all aspects of the work.

Lionel Tarassenko: Substantial contributions to the conception and design of the work. Substantial contributions to the acquisition, analysis, and interpretation of data for the work. Drafting the work and revising it critically for important intellectual content. Final approval of the version to be published. Agreement to be accountable for all aspects of the work.

Andrew Walden: Substantial contributions to the acquisition of data for the work. Revising this manuscript critically for important intellectual content. Final approval of the manuscript. Agreement to be accountable for all aspects of the work.

Peter J Watkinson: Substantial contributions to the conception and design of the work. Substantial contributions to the acquisition, analysis, and interpretation of data for the work. Drafting the work and revising it critically for important intellectual content. Final approval of the version to be published. Agreement to be accountable for all aspects of the work.

J Duncan Young: Substantial contributions to the conception and design of the work. Substantial contributions to the acquisition, analysis, and interpretation of data for the work. Drafting the work and revising it critically for important intellectual content. Final approval of the version to be published. Agreement to be accountable for all aspects of the work.

For peer review only

Acknowledgements

Dr Jonathan Burgess, who sadly passed away before seeing the fruits of his labours, undertook much of the early work on the identification of candidate variables in this project.

For peer review only

References

1. Schein RMH, Hazday N, Pena M, et al.: Clinical Antecedents to In-Hospital Cardiopulmonary Arrest. *Chest* 1990; 98:1388–1392

2. McQuillan P, Pilkington S, Allan A, et al.: Confidential inquiry into quality of care before admission to intensive care. *BMJ* 1998; 316:1853–1858

3. Kause J, Smith G, Prytherch D, et al.: A comparison of Antecedents to Cardiac Arrests, Deaths and EMergency Intensive care Admissions in Australia and New Zealand, and the United Kingdom—the ACADEMIA study. *Resuscitation* 2004; 62:275–282

4. Churpek MM, Yuen TC, Huber MT, et al.: Predicting cardiac arrest on the wards: A nested case-control study. *Chest* 2012;

5. Berlot G, Pangher A, Petrucci L, et al.: Anticipating events of in-hospital cardiac arrest. *Eur J Emerg Med* 2004; 11:24–28

6. Morgan R, Williams F, Wright M: An early warning scoring system for detecting developing critical illness. *Clin Intensive Care* 1997; 8:100

7. Smith GB, Prytherch DR, Schmidt PE, et al.: Review and performance evaluation of aggregate weighted “track and trigger” systems. *Resuscitation* 2008;

8. Alam N, Hobbelink EL, van Tienhoven AJ, et al.: The impact of the use of the Early Warning Score (EWS) on patient outcomes: A systematic review. *Resuscitation* 2014; 85:587–594

9. National Institute for Health and Care Excellence (NICE): Acutely ill adults in hospital: recognising and responding to deterioration. *Clin Guidel* 2007;

10. Adam S, Odell M: An acute problem? A report of the National Confidential Enquiry into Patient Outcome and Death. *Nurs Crit Care* 2005; 10:225–227

11. National Institute for Health and Care Excellence (NICE): Sepsis: recognition, diagnosis and early management. *BJU Int* 2018; 121:497–514

12. Christofidis MJ, Hill A, Horswill MS, et al.: Observation charts with overlapping blood pressure and heart rate graphs do not yield the performance advantage that health professionals assume: an experimental study. *J Adv Nurs* 2014; 70:610–624

13. Elliott D, Allen E, Perry L, et al.: Clinical user experiences of observation and response charts: focus group findings of using a new format chart incorporating a track and trigger system. *BMJ Qual Saf* 2015; 24:65–75

14. Smith GB, Prytherch DR, Schmidt P, et al.: Hospital-wide physiological surveillance-A new approach to the early identification and management of the sick patient. *Resuscitation* 2006;

15. Wong D, Bonnici T, Knight J, et al.: SEND: a system for electronic notification and documentation of vital sign observations. *BMC Med Inform Decis Mak* 2015; 15:68

16. Bleyer AJ, Vidya S, Russell GB, et al.: Longitudinal analysis of one million vital

For peer review only

signs in patients in an academic medical center. *Resuscitation* 2011;

17. Green M, Lander H, Snyder A, et al.: Comparison of the Between the Flags calling criteria to the MEWS, NEWS and the electronic Cardiac Arrest Risk Triage (eCART) score for the identification of deteriorating ward patients. *Resuscitation* 2018; 123:86–91

18. Moons KGM, Altman DG, Reitsma JB, et al.: Transparent Reporting of a multivariable prediction model for Individual Prognosis Or Diagnosis (TRIPOD): Explanation and Elaboration. *Ann Intern Med* 2015; 162:W1

19. Intensive Care National Audit & Research Centre (ICNARC): About the CMP. *Accessed from https//www.icnarc.org/Our-Audit/Audits/Cmp/About; Accessed July 2020*

20. Prytherch DR, Smith GB, Schmidt PE, et al.: ViEWS-Towards a national early warning score for detecting adult inpatient deterioration. *Resuscitation* 2010; 81:932–937

21. Smith GB, Prytherch DR, Meredith P, et al.: The ability of the National Early Warning Score (NEWS) to discriminate patients at risk of early cardiac arrest, unanticipated intensive care unit admission, and death. *Resuscitation* 2013; 84:465–470

22. Pimentel MAF, Clifton DA, Clifton L, et al.: A review of novelty detection. *Signal Processing* 2014; 99:215–249

23. Johnson AEW, Burgess J, Pimentel MAF, et al.: Physiological trajectory of patients pre and post ICU discharge. In: 2014 36th Annual International Conference of the IEEE Engineering in Medicine and Biology Society. IEEE; 2014. p. 3160–3163.

24. Pimentel MAF, Clifton DA, Clifton L, et al.: Modelling physiological deterioration in post-operative patient vital-sign data. *Med Biol Eng Comput* 2013;

25. Lemeshow S, Hosmer DW: A review of goodness of fit statistics for use in the development of logistic regression models. *Am J Epidemiol* 1982;

26. Kramer AA, Zimmerman JE: Assessing the calibration of mortality benchmarks in critical care: The Hosmer-Lemeshow test revisited*. *Crit Care Med* 2007; 35:2052–2056

27. Cox DR: Two further applications of a model for binary regression. *Biometrika* 1958; 45:562–565

28. DiCiccio TJ, Efron B: Bootstrap confidence intervals. *Stat Sci* 1996;

29. Gardner-Thorpe J, Love N, Wrightson J, et al.: The value of Modified Early Warning Score (MEWS) in surgical in-patients: A prospective observational study. *Ann R Coll Surg Engl* 2006; 88:571–575

30. Paterson R, MacLeod DCD, Thetford D, et al.: Prediction of in-hospital mortality and length of stay using an early warning scoring system: clinical audit. *Clin Med J R Coll Physicians London* 2006; 6:281–284

31. Watkinson PJ, Pimentel MAF, Clifton DA, et al.: Manual centile-based early warning scores derived from statistical distributions of observational vital-sign data. *Resuscitation* 2018; 129:55–60

For peer review only

32. Akre M, Finkelstein M, Erickson M, et al.: Sensitivity of the Pediatric Early Warning Score to Identify Patient Deterioration. *Pediatrics* 2010; 125:e763–e769

33. Skaletzky SM, Raszynski A, Totapally BR: Validation of a Modified Pediatric Early Warning System Score. *Clin Pediatr (Phila)* 2012; 51:431–435

34. Hravnak M, Devita MA, Clontz A, et al.: Cardiorespiratory instability before and after implementing an integrated monitoring system. *Crit Care Med* 2011;

35. Gneiting T, Ranjan R: Combining predictive distributions. *Electron J Stat* 2013;

36. Akhondi-Asl A, Hoyte L, Lockhart ME, et al.: A logarithmic opinion pool based STAPLE algorithm for the fusion of segmentations with associated reliability weights. *IEEE Trans Med Imaging* 2014;

37. Hosein FS, Bobrovitz N, Berthelot S, et al.: A systematic review of tools for predicting severe adverse events following patient discharge from intensive care units. *Crit Care* 2013;

38. Daly K: Reduction in mortality after inappropriate early discharge from intensive care unit: logistic regression triage. *BMJ* 2001; 322:1274–1274

39. Fernandez R, Serrano JM, Umaran I, et al.: Ward mortality after ICU discharge: A multicenter validation of the Sabadell score. *Intensive Care Med* 2010;

40. Tarassenko L, Clifton DA, Pinsky MR, et al.: Centile-based early warning scores derived from statistical distributions of vital signs. *Resuscitation* 2011; 82:1013–1018

41. Churpek MM, Yuen TC, Edelson DP: Predicting clinical deterioration in the hospital: The impact of outcome selection. *Resuscitation* 2013; 84:564–568

42. Redfern OC, Pimentel MAF, Prytherch D, et al.: Predicting in-hospital mortality and unanticipated admissions to the intensive care unit using routinely collected blood tests and vital signs: Development and validation of a multivariable model. *Resuscitation* 2018; 133:75–81

43. Subbe CP: Validation of a modified Early Warning Score in medical admissions. *QJM* 2001; 94:521–526

44. Jarvis SW, Kovacs C, Briggs J, et al.: Are observation selection methods important when comparing early warning score performance? *Resuscitation* 2015; 90:1–6

45. Romero-Brufau S, Huddleston JM, Escobar GJ, et al.: Why the C-statistic is not informative to evaluate early warning scores and what metrics to use. *Crit Care* 2015; 19:285

46. Subbaswamy A, Schulam P, Saria S: Preventing Failures Due to Dataset Shift: Learning Predictive Models That Transport. *AISTATS 2019 - 22nd Int Conf Artif Intell Stat* 2018;

For peer review only

Additional files

Additional file 1

File name: Additional file 1

File format: .docx

Title: Model development

Description: Detailed description of the methods used to develop the models.

Additional file 2

File name: Additional file 2

File format: .docx

Title: Comparative scoring systems

Description: Description of the components, weightings and details of other scoring systems implemented in this study.

Additional file 3

File name: Additional file 3

File format: .docx

Title: Additional results

Description: Further results of the performance of the models.

For peer review only

Tables Legends

Table 1. Details of the ICU admissions and the subgroups of post-ICU admissions included in the development and validation cohorts. Continuous (numeric) variables are displayed as *median [interquartile range] (mean)*, and count variables are displayed as *counts (%)*. "OUH" refers to admissions from the Oxford University Hospitals NHS Foundation Trust's two adult ICUs. "RBH" refers to admissions from the Royal Berkshire NHS Foundation Trust's single adult ICU. "LoS" denotes length of stay.

Table 2. Performance of the first model (RS_1) on both development and validation datasets for the combined outcome (or adverse event) of in-hospital death or re-admission to ICU at any point after discharge from ICU. Performance for each event separately are also displayed. Cox's calibration regression: for a good calibration, α should be close to 0, and β should be close to 1. AUROC denotes area under the receiver operating characteristic curve, shown with mean (standard deviation, SD).

Table 3. Area under the receiver operating characteristic curve (AUROC), standard deviation and corresponding 95% confidence interval (CI) for the developed risk scoring index (RSI) and other competitive early warning score (EWS) systems, using adverse event (re-admission to ICU or in-hospital death) within 24h of an observation set as the (compound) outcome. Results are shown for each outcome independently using both development and validation datasets.

Figure Legends

Figure 1. Diagram showing the construction of the study cohorts from both organisations (OUH and RBH) included in the study by applying inclusion/exclusion criteria.

Figure 2. Representation of a set of the variables acquired for an example patient included in our study pre (represented with circles) and post discharge from the ICU. Variables include vital signs (dark grey), laboratory tests (lighter grey) and interventions/treatments (white) performed. The grey vertical line marks the patient's ICU discharge timepoint. A more detailed electronic patient record is "generated" during the patient's ICU stay. We also note the different frequency of measurement of the vital signs in the ICU from that on the ward.

For peer review only

Figure 3. Performance of the developed risk scoring system (RSI) and the other competitive scoring systems considered for predicting an adverse event (either re-admission to ICU or in-hospital death) within 12, 24, 36, 48, and 72h of a vital-sign observation set. The left-hand panel shows the performance on the development dataset, the right-hand panel shows the performance on the validation dataset. The performance is represented with the mean AUROC (area under the receiver operating characteristic curve) values.

For peer review only

Table 1.

	Development (OUH)	Validation (RBH)
**ICU admissions included, n**	**8,162**	**3,421**
Age, years	62 [47 – 72] (59)	65 [51 – 75] (62)
Females sex, n	3,381 (41.4%)	1,480 (43.3%)
LoS in ICU, days	1.9 [1.0 – 4.0] (4.2)	2.0 [1.0 – 4.5] (4.2)
Outcome:		
ICU re-admission, n	543 (6.7%)	154 (4.5%)
Time to ICU re-admission, hours	92 [37 – 205] (225)	101 [43 – 283] (340)
In-hospital death, n	462 (5.7%)	218 (6.4%)
Time to in-hospital death, hours	296 [84 – 781] (701)	204 [66 – 600] (540)
Adverse event, n	902 (11.1%)	329 (9.6%)
**Post-ICU admissions included, n**	**273**	**136**
Age, year	64 [51 – 72] (60)	66 [55 – 73] (63)
Females sex, n	85 (31.1)	61 (45)
LoS in ICU, days	1.9 [1.0 – 3.9] (3.5)	1.3 [0.9 – 3.0] (3.4)
Outcome:		
ICU re-admission, n	18 (6.6%)	9 (6.6%)
Time to ICU re-admission, hours	162 [73 – 220] (215)	108 [72 – 202] (153)
In-hospital death, n	6 (2.2%)	4 (2.9%)
Time to in-hospital death, hours	216 [66 – 514] (356)	322 [244 – 365] (288)
Adverse event, n	23 (8.4%)	11 (8.1%)

For peer review only - http://bmjopen.bmj.com/site/about/guidelines.xhtml

Table 2.

	Development dataset		
	**Adverse event**	**In-hospital death**	**ICU re-admission**
Hosmer-Lemeshow statistic	50.81	339.07	224.94
Brier score	0.087	0.050	0.065
Cox's calibration:			
α	-0.009	-0.037	0.013
β	0.988	1.104	0.472
AUROC (SD)	0.782 (0.022)	0.843 (0.022)	0.716 (0.022)
	**Validation dataset**		
Hosmer-Lemeshow statistic	57.35	175.68	275.08
Brier score	0.081	0.058	0.058
Cox's calibration:			
α	0.017	-0.012	0.033
β	0.627	0.475	0.268
AUROC (SD)	0.723 (0.014)	0.780 (0.017)	0.623 (0.019)

For peer review only

Table 3.

	Development dataset		
	Adverse event	In-hospital death	ICU re-admission
MEWS	0.749 (0.022) [0.734 to 0.764]	0.752 (0.055) [0.717 to 0.789]	0.747 (0.021) [0.733 to 0.761]
SEWS	0.754 (0.019) [0.741 to 0.765]	0.785 (0.050) [0.753 to 0.819]	0.744 (0.022) [0.730 to 0.759]
NEWS	0.757 (0.020) [0.742 to 0.771]	0.889 (0.030) [0.867 to 0.912]	0.737 (0.022) [0.721 to 0.753]
CEWS	0.761 (0.021) [0.748 to 0.775]	0.887 (0.029) [0.866 to 0.908]	0.740 (0.022) [0.727 to 0.754]
RS_2	0.767 (0.019) [0.755 to 0.780]	0.859 (0.041) [0.836 to 0.889]	0.745 (0.020) [0.732 to 0.758]
**RSI**	**0.782 (0.018) [0.770 to 0.794]**	**0.931 (0.007) [0.926 to 0.936]**	**0.753 (0.017) [0.743 to 0.764]**
	**Validation dataset**		
MEWS	0.632 (0.032) [0.610 to 0.655]	0.697 (0.054) [0.661 to 0.733]	0.632 (0.033) [0.610 to 0.655]
SEWS	0.678 (0.037) [0.653 to 0.703]	0.678 (0.057) [0.642 to 0.717]	0.681 (0.038) [0.657 to 0.706]
NEWS	0.653 (0.044) [0.621 to 0.683]	0.675 (0.063) [0.630 to 0.719]	0.649 (0.043) [0.622 to 0.679]
CEWS	0.672 (0.036) [0.648 to 0.695]	0.685 (0.061) [0.643 to 0.728]	0.670 (0.036) [0.645 to 0.596]
RS_2	0.693 (0.040) [0.669 to 0.718]	0.681 (0.059) [0.644 to 0.722]	0.693 (0.041) [0.666 to 0.721]
**RSI**	**0.724 (0.027) [0.704 to 0.741]**	**0.725 (0.045) [0.694 to 0.757]**	**0.722 (0.028) [0.702 to 0.741]**

For peer review only

For peer review only

For peer review only

For peer review only - http://bmjopen.bmj.com/site/about/guidelines.xhtml

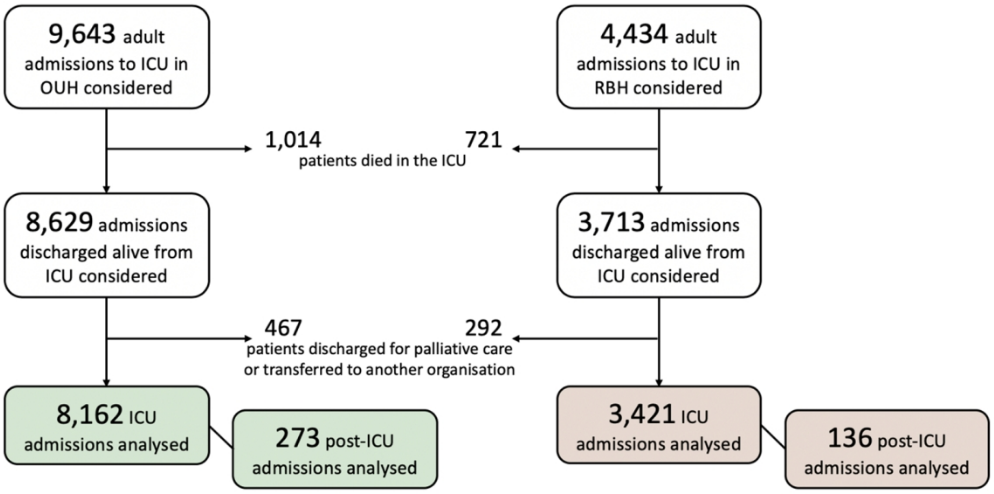

Figure 1. Diagram showing the construction of the study cohorts from both organisations (OUH and RBH) included in the study by applying inclusion/exclusion criteria.

715x360mm (57 x 57 DPI)

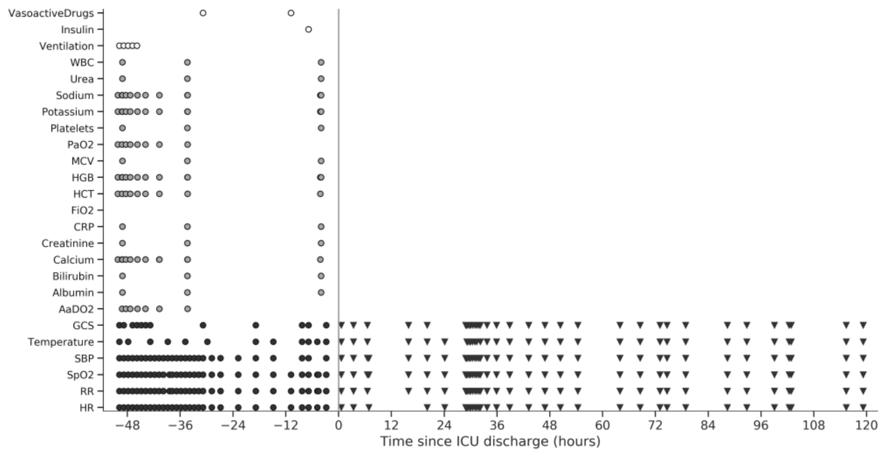

Figure 2. Representation of a set of the variables acquired for an example patient included in our study pre (represented with circles) and post discharge from the ICU. Variables include vital signs (dark grey), laboratory tests (lighter grey) and interventions/treatments (white) performed. The grey vertical line marks the patient's ICU discharge timepoint. A more detailed electronic patient record is "generated" during the patient's ICU stay. We also note the different frequency of measurement of the vital signs in the ICU from that on the ward.

966x515mm (138 x 138 DPI)

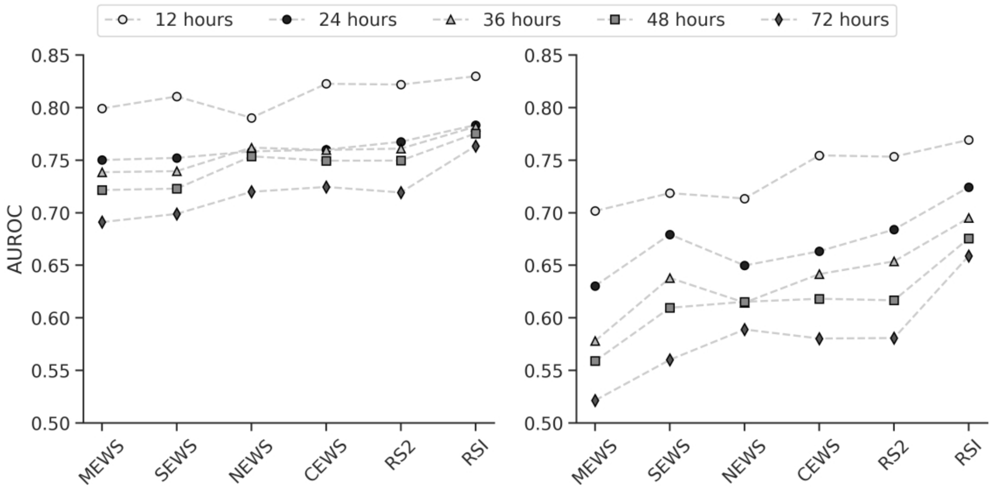

Figure 3. Performance of the developed risk scoring system (RSI) and the other competitive scoring systems considered for predicting an adverse event (either re-admission to ICU or in-hospital death) within 12, 24, 36, 48, and 72h of a vital-sign observation set. The left-hand panel shows the performance on the development dataset, the right-hand panel shows the performance on the validation dataset. The performance is represented with the mean AUROC (area under the receiver operating characteristic curve) values.

662x331mm (138 x 138 DPI)

Additional file 1 – Model development

We describe in more detail the methods used to develop the model in this section.

Predictors

Our proposed approach uses a conceptual model in which we estimate the risk of a patient experiencing an adverse event immediately after discharge from ICU. This risk score (RS_1) is estimated from variables recorded during their stay in the ICU. After discharge from ICU, the patient's risk of experiencing an adverse event within the next 24 hours is updated in a time-varying manner (RS_2), using those measurements of the vital signs that are routinely measured during subsequent ward care.

For peer review only

ICU-based feature representation:

To estimate the risk of a patient experiencing an adverse event immediately after discharge from ICU, we considered candidate variables that were available electronically in our databases. The final list of candidate variables included the following physiological variables:

- Heart rate (HR), measured in beats per minute
- Respiratory rate (RR), in breaths per minute
- Systolic (SBP), diastolic and mean blood pressure, in mmHg
- Temperature, in degrees Celsius
- Peripheral oxygen saturation (SpO_2), in %
- Level of consciousness, using the Glasgow Coma Scale (GCS)

The following laboratory test results were included in the list:

- Alveolar-arterial oxygen partial pressure gradient ($AaDO_2$), in kPa
- Albumin, in g/L
- Bilirubin (total), in μmol/L
- Calcium (adjusted), in mmol/L
- Creatinine, in mmol/L
- C-reactive protein (CRP), in mg/L
- Haematocrit (HCT), in %
- Haemoglobin (HGB), in g/dL
- Lactate, in mmol/L
- Mean corpuscular volume (MCV), in fL
- Ratio of partial pressure of oxygen : fraction of inspired oxygen (PaO_2/FiO_2), in kPa
- pH
- Platelet count, in 10^9 cells/L

- Potassium, in mmol/L
- Sodium, in mmol/L
- Urea, measured in mmol/L
- Urine output, in mL
- White blood cell count (WBC), in 10^9 cells/L

The list also included demographic and administrative-based variables, such as age at admission, gender, number of hours between hospital admission and ICU admission, and the Index of Multiple Deprivation score (1), derived for patients with a valid postcode. We included variables related to procedures and treatments, such as the number of vasoactive drugs administered, total fluid balance, administration of insulin, enteral and parenteral nutrition feeding, and the use of mechanical ventilation, tracheostomy and central venous catheters.

To determine the risk of future compound outcome after discharge from the ICU, we derived several features from all candidate variables acquired during the patients' stay in the ICU. These features are based on the extremes of the variables considered. We generated maximum, minimum and variability (as given by the standard deviation) features for the physiological variables and laboratory test results from different periods of the ICU stay (including the first 24 hours of the patient in the ICU, their last 72, 48, and 24 hours, and/or their entire ICU stay). Additional dichotomous variables were generated if the values were in the upper or lower 5th percentile of the observed corresponding ranges, in order to account for potential non-linear associations of the variables with the adverse outcomes. Procedure and treatment variables were converted to dichotomous features for indicating whether a given procedure was performed or not, or whether a given medication was administered or not, over the entire ICU stay or in the last 24 hours of the ICU stay. This procedure resulted in 161 candidate features (including features from demographic information) for building a prediction model.

<u>Post-ICU feature representation</u>:

All vital-sign observations performed after discharge from ICU, as part of routine patient monitoring on acute care wards and collected prospectively for this study, were considered for analysis. Each set of vital signs include heart rate, systolic blood pressure, respiratory rate, body temperature, neurological status assessment using the Alert-Verbal-Painful-Unresponsive (AVPU) scale, peripheral oxygen saturation from pulse oximetry (SpO_2), a record of whether the patient was receiving supplemental oxygen at the time of the SpO_2 measurement, and the date and time of the observation. Vital-sign measurements are typically recorded every 4 or 6 hours throughout the patient's stay on the ward. At each measurement timestamp, in case of an incomplete vital-sign observation set, we used the most recent value of each variable (i.e., by carrying forward the last measurement).

For peer review only

Pre-processing and missing data

We identified obvious deviations from expected distributions and ranges of the data features using frequency graphs for all numerical and dichotomous features. Possible physiological ranges for the numerical features were defined according to clinical review and expert panel expertise, and values outside these ranges were not included in the analyses. Missing values were imputed with median and mode values from the feature distributions of numerical and dichotomous features, respectively. While other methods were considered, such as multiple imputation, the use of the median and mode was simpler and was deemed sufficient for this work in which the amount of missing data was low.

All numerical features were then standardised using a zero-mean, unit-variance transformation (i.e., using the mean and standard deviations from the feature distributions). This prevents features with relatively small changes in their units of measurement (such as temperature) from being dominated by features with relatively large changes (such as blood pressure), thus ensuring that all features have similar dynamic ranges.

For both imputation and normalisation, the parameters' values (for the median, mode, mean and standard deviation) found for the development dataset were used.

ICU-based model (RS_1)

We used all candidate features derived from the variables acquired during the patients' stay in the ICU to build the first model. A L1-regularised logistic regression using the "glmnet" package in Matlab (Mathworks, Natick, Massachusetts, USA) for predicting the compound outcome (in-hospital death or re-admission to ICU). L1-regularisation shrinks the less important features' coefficients to zero thus effectively removing those features that are deemed to be uninformative to the outcome variable. We estimated the regularisation parameter using LASSO (Least Absolute Shrinkage and Selection Operator) regression in a 5-fold cross-validation on the development set.

Vital-sign based model (RS_2)

For the second scoring system, RS_2, we used the vital signs (heart rate, respiratory rate, systolic blood pressure, oxygen saturation, and temperature) recorded in hospital after discharge from the ICU. For this model, rather than a *supervised learning approach*, we considered a novelty detection method (or one-class classification method), which does not require an outcome variable for development (2). This is useful where event rates are extremely low, or total sample sizes are constrained. Similar approaches have been used in our previous research (3–6) and underpin a commercial, clinically-used system, described in (7).

For peer review only

We considered the construction of a multivariate, (possibly) multimodal model of normality, based on the vital-sign observation sets recorded during the 24 hours that preceded discharge home. If the patient had not died, was not re-admitted to the ICU and was not discharged by the 14th post-ICU day, we used recorded data on that day. These observation sets are thus assumed to contain the vital-sign values from the most stable period of the patient's hospital stay, because these data were acquired when patients are at their most "stable" after discharge from ICU. This set of "normal" data contains $N = 1{,}082$ 5-dimensional vital-sign vectors, $\mathbf{X} = \{\mathbf{x_1}, \dots, \mathbf{x_N}\} \in \mathbb{R}^5$, which were subsequently used for the construction of our model of normality.

A kernel density estimate, or KDE (2) was used to estimate the probability density function (pdf) of the set of five vital signs. This is in a non-parametric technique where no *a priori* assumptions about the form of the underlying probability distribution are made. Our notation follows that reported previously (5, 6). The data pdf $p(\mathbf{x})$ was estimated using the $N = 1{,}082$ set of observations as shown in (SM1-1).

$$p(\mathbf{x}|\mathbf{x}_i, \sigma) = \frac{1}{N(2\pi)^{D/2}\sigma^D} \sum_{i=1}^{N} \exp\left(\frac{-|\mathbf{x}-\mathbf{x}_i|^2}{2\sigma^2}\right) \qquad \text{(SM1-1)}$$

This is a weighted sum of Gaussian kernels, each with identical variance σ^2 (i.e., isotropic kernels), centred on the observation sets $\mathbf{x_1}, \dots, \mathbf{x_N}$.

The nearest-neighbour method was used to estimate the variance. Briefly, this method involves determining the squared Euclidean distance (Δ) for each observation i to its 10 nearest neighbours (*NNs*), as shown in (SM1-2).

$$\Delta_i = \frac{1}{10} \sum_{j \in \{NNs\}} \left\| \mathbf{x}_i - \mathbf{x}_j \right\| \qquad \text{(SM1-2)}$$

This quantity, Δ, is then used to estimate the variance σ^2, as shown in (SM1-3).

$$\sigma = \frac{1}{N} \sum_{i=1}^{N} \Delta_i \qquad \text{(SM1-3)}$$

Estimation of the underlying pdf of normal vital-sign data provides a means of quantifying the degree to which a given set of observations is abnormal. The likelihood $p(\mathbf{x}|\mathbf{x}_i, \sigma)$, a measure which represents the probability of observing a set of measurements given a pdf, can be used for this purpose. Thus, we define the novelty score as in (SM1-4).

$$z(\mathbf{x}) = -\log p(\mathbf{x}|\mathbf{x}_i, \sigma) \qquad \text{(SM1-4)}$$

For normal data, the new observation $\mathbf{x}$ will be similar to previously-seen normal observations $\mathbf{x}_i$, and so the likelihood will be high. Consequently, the negative log-likelihood will be low, and so the novelty score $z(\mathbf{x})$ will be low. Conversely, for abnormal data, the data will be dissimilar and the likelihood will be low, and consequently the novelty score $z(\mathbf{x})$ will be high. This procedure ensures that the

For peer review only

novelty score can be interpreted as most early warning scores (EWS), in that high scores are associated with higher abnormality of the vital signs.

In short, this model allows the generation of a score corresponding to an assessment of whether a vital-sign set should be deemed "stable" with respect to the development dataset (i.e., the observation sets used for training the model). By extension, it further allows the model to estimate the degree of abnormality of a given vital-sign observation set.

Risk Score Index (RSI)

An overall risk score, the risk score index (RSI), was subsequently determined using a simple time-dependent linear combination of the two constituent risk scores, such that:

$$\mathrm{RSI} = \beta\left[\left(1 - \frac{t}{Tmax}\right)\mathrm{RS}_1\right] + \left[\left(\frac{t}{Tmax}\right)\mathrm{RS}_2\right] \quad \text{(Equation 1)}$$

where t corresponds to the elapsed time (in hours) since the patient was discharged from ICU and has a maximum value of $Tmax$ hours. The time-dependent weighting function allows the contribution of RS_1 to gradually decrease with time after discharge from the ICU. The patient's risk of future adverse events becomes increasingly determined by the values of the vital-sign measurements taken on the acute ward (i.e., RS_2). The parameter β is used to adjust the weight of RS_1 with respect to the time since discharge from ICU, and the parameter $Tmax$ corresponds to the time at which RS_1 no longer affects RSI; i.e., when $t \geq Tmax$, then $\mathrm{RSI} = \mathrm{RS}_2$. The parameters β and $Tmax$ were determined using a grid-search algorithm with 3-fold cross-validation, by defining a grid of possible values of $\beta = [0.1,\ 0.2,\ 0.3, \ldots, 200]$ and $Tmax = [12, 24, 36, \ldots, 336]$. We selected the values of β and $Tmax$ that corresponded to the highest mean area under the curve of the receiver operating characteristic (AUROC) value across all cross-validation folds, using the compound outcome of in-hospital death or ICU re-admission within the next 24 hours of a vital-sign observation.

During the development of RSI, values of $\beta = 100.2$ and $Tmax = 96$ hours, for the linear time-dependent weighting function (see Equation 1), were obtained and used to calculate the risk scoring index.

Model validation and statistical analysis

To validate the first model, RS_1, its discrimination and calibration were analysed. Discrimination is defined as the ability of the model to separate non-event patients from patients who had an adverse event after ICU discharge, and was assessed

For peer review only

using the AUROC metric. Calibration assesses the degree of correspondence between the estimated probability of occurrence of an adverse event and that actually observed. This was tested using a goodness of fit test, the Hosmer-Lemeshow "C" statistic (8). When the predicted probability of adverse events of the prognostic model differs significantly from the observed pattern, the calibration ability of the model is deemed to be poor. As the Hosmer-Lemeshow test does not measure the magnitude of miscalibration and is sensitive to sample size (9), calibration was also assessed with the Brier score and Cox's calibration regression. The latter assesses the degree of miscalibration by fitting a logistic regression of observed survival to the predicted log-odds of survival from the risk model (10). The performance of the first model was examined both for the compound outcome and each adverse event (in-hospital death and ICU re-admission) individually. The ability of the first model to predict future adverse events at increasing intervals from ICU discharge was also examined by calculating the AUROC for future events by day after discharge (up to 120 days).

The final model, RSI, was validated using the AUROC for the derived outcome of in-hospital death or ICU re-admission within the next *N* hours of a vital-sign observation/measurement performed after ICU discharge, in line with previous studies for evaluating EWS systems (11, 12). We evaluated the model for different values of *N*, with $N = [12, 24, 36, 48, 72]$ hours. We note that in this case the AUROC represents how well the scoring system RSI discriminates between observation sets followed by an adverse event and those with no subsequent adverse outcome within the next *N* hours. Therefore, the unit of analysis is a vital-sign set rather than a patient-admission, as performed for the validation of the first model.

We also considered each individual adverse event separately. To further understand the feasibility of implementation of the risk scoring systems in this setting, the burden of observation sets "triggered" for every correctly identified observation followed by an adverse event within 24 hours by the risk scoring system was also evaluated.

We report the cross-validation results using the development dataset. This gives an estimate of how our models perform on a random set of samples from the OUH Trust that were not used for developing the model. We also report the external validation results using data from the RBH Trust. Confidence intervals were estimated using bootstrap confidence intervals via percentiles with 500 samples (13).

References

1. GOV.UK (National Statistics): English indices of deprivation 2015. *Accessed from https//www.gov.uk/government/statistics/english-indices-of-deprivation-2015; Accessed January 2018*
2. Pimentel MAF, Clifton DA, Clifton L, et al.: A review of novelty detection. *Signal Processing* 2014; 99:215–249

For peer review only

3. Watkinson PJ, Barber VS, Price JD, et al.: A randomised controlled trial of the effect of continuous electronic physiological monitoring on the adverse event rate in high risk medical and surgical patients. *Anaesthesia* 2006;

4. Clifton DA, Wong D, Clifton L, et al.: A large-scale clinical validation of an integrated monitoring system in the Emergency Department. *IEEE J Biomed Heal Informatics* 2013;

5. Johnson AEW, Burgess J, Pimentel MAF, et al.: Physiological trajectory of patients pre and post ICU discharge. In: 2014 36th Annual International Conference of the IEEE Engineering in Medicine and Biology Society. IEEE; 2014. p. 3160–3163.

6. Pimentel MAF, Clifton DA, Clifton L, et al.: Modelling physiological deterioration in post-operative patient vital-sign data. *Med Biol Eng Comput* 2013;

7. Orphanidou C, Clifton D, Shahab Khan, et al.: Telemetry-based vital sign monitoring for ambulatory hospital patients. In: 2009 Annual International Conference of the IEEE Engineering in Medicine and Biology Society. IEEE; 2009. p. 4650–4653.

8. Lemeshow S, Hosmer DW: A review of goodness of fit statistics for use in the development of logistic regression models. *Am J Epidemiol* 1982;

9. Kramer AA, Zimmerman JE: Assessing the calibration of mortality benchmarks in critical care: The Hosmer-Lemeshow test revisited*. *Crit Care Med* 2007; 35:2052–2056

10. Cox DR: Two further applications of a model for binary regression. *Biometrika* 1958; 45:562–565

11. Prytherch DR, Smith GB, Schmidt PE, et al.: ViEWS-Towards a national early warning score for detecting adult inpatient deterioration. *Resuscitation* 2010; 81:932–937

12. Badriyah T, Briggs JS, Meredith P, et al.: Decision-tree early warning score (DTEWS) validates the design of the National Early Warning Score (NEWS). *Resuscitation* 2014; 85:418–423

13. DiCiccio TJ, Efron B: Bootstrap confidence intervals. *Stat Sci* 1996;

For peer review only

Additional file 2 – Comparative scoring systems

In this section, we described the details of other scoring systems used in this study and how they were implemented using the information included in datasets. We also detail the components and weightings of the individual scores, as they were implemented in this work.

The early warning score (EWS) systems evaluated in this study include the modified EWS, or MEWS (1), the standardised EWS, or SEWS (2), the National EWS, or NEWS (3, 4) and the centile-based EWS, or CEWS (5). Their components and weightings are shown in tables SM2-1, SM2-2, SM2-3 and SM2-4, respectively. The core set of variables that are used in these scoring systems comprises heart rate (measured in beats per minute), respiratory rate (in breaths per minute), systolic and diastolic blood pressure (in mmHg), body temperature (in °C), oxygen saturation (denoted "SpO_2", in %), an assessment of neurological status (using the Alert-Verbal-Painful-Unresponsive or AVPU scale), a flag for whether the patient was receiving supplementary oxygen at the time of the SpO_2 measurement (denoted "Inspired O_2"), and the age to the patient at admission to hospital. At each observation set (or measurement timestamp), in case of an incomplete vital-sign observation set, we used the most recent value of each variable. Remaining missing data were dealt with as with the proposed scoring system (see section 2 in the main manuscript).

Table SM2-1. Modified Early Warning Score (see main text for units of the variables included).

Table SM2-2. Standardised Early Warning Score (see main text for units of the variables included).

Table SM2-3. National Early Warning Score (see main text for units of the variables included).

Table SM2-4. Centile-based Early Warning Score (see main text for units of the variables included).

For peer review only

Table SM2-1.

Modified Early Warning Score (MEWS)

Variable	Score 3	2	1	0	1	2	3
Heart rate	≤ 39	40 – 50	51 – 59	60 – 100	101 – 110	111 – 129	≥ 130
Resp. rate		≤ 8		9 – 14	15 – 20	21 – 29	≥ 30
Temperature		≤ 35.0		35.1 – 38.0		38.1 – 39.5	≥ 39.6
Systolic BP	≤ 69	70 – 80	81 – 100	101 – 149	150 – 169	170 – 179	≥ 180
AVPU scale				A	V	P	U

Table SM2-2.

Standardised Early Warning Score (SEWS)

Variable	Score 3	2	1	0	1	2	3
Heart rate	≤ 29	30 – 39	40 – 49	50 – 99	100 – 109	110 – 129	≥ 130
Resp. rate	≤ 8			9 – 20	21 – 30	31 – 35	≥ 36
Temperature	≤ 33.9	34.0 – 34.9	35.0 – 35.9	36.0 – 37.9	38.0 – 38.9	≥ 39.0	
Systolic BP	≤ 69	70 – 79	80 – 99	100 – 199		≥ 200	
SpO_2	≤ 84	85 – 89	90 – 92	≥ 93			
AVPU scale				A	V	P	U

Table SM2-3.

National Early Warning Score (NEWS)

Variable	Score 3	2	1	0	1	2	3
Heart rate	≤ 40		41 – 50	51 – 90	91 – 110	111 – 130	≥ 131
Resp. rate	≤ 8		9 – 11	12 – 20		21 – 24	≥ 25
Temperature	≤ 35.0		35.1 – 36.0	36.1 – 38.0	38.1 – 39.0	≥ 39.1	
Systolic BP	≤ 90	91 – 100	101 – 110	111 – 219			≥ 220
SpO_2	≤ 91	92 – 93	94 – 95	≥ 96			
Inspired O_2		Any O_2		Air			
AVPU scale				A			V, P, U

Table SM2-4.

Centile-based Early Warning Score (CEWS)

Variable	Score 3	2	1	0	1	2	3
Heart rate	≤ 42	43 – 49	50 – 53	54 – 104	105 – 112	113 – 127	≥ 128
Resp. rate	≤ 7	8 – 10	11 – 12	13 – 21	22 – 23	24 – 28	≥ 29
Temperature	≤ 35.4		35.5 – 35.9	36.0 – 37.3	37.4 – 38.3		≥ 38.4
Systolic BP	≤ 83	84 – 90	91 – 100	101 – 157	158 – 167	168 – 184	≥ 185
SpO_2	≤ 84	85 – 90	91 – 93	≥ 94			
Inspired O_2		Any O_2		Air			
AVPU scale				A	V		P, U

References

1. Gardner-Thorpe J, Love N, Wrightson J, et al.: The value of Modified Early Warning Score (MEWS) in surgical in-patients: A prospective observational study. *Ann R Coll Surg Engl* 2006; 88:571–575
2. Paterson R, MacLeod DCD, Thetford D, et al.: Prediction of in-hospital mortality and

For peer review only

length of stay using an early warning scoring system: clinical audit. *Clin Med J R Coll Physicians London* 2006; 6:281–284
3. Smith GB, Prytherch DR, Meredith P, et al.: The ability of the National Early Warning Score (NEWS) to discriminate patients at risk of early cardiac arrest, unanticipated intensive care unit admission, and death. *Resuscitation* 2013; 84:465–470
4. Royal College of Physicians (RCP): National Early Warning Score (NEWS). *Stand Assess acute-illness Sev NHS* 2012; 47
5. Watkinson PJ, Pimentel MAF, Clifton DA, et al.: Manual centile-based early warning scores derived from statistical distributions of observational vital-sign data. *Resuscitation* 2018; 129:55–60

For peer review only

Additional file 3 – Additional results

In this section, we describe in more detail some of the results of the study.

ICU-based model (RS_1)

The features computed from the data acquired during the patients' ICU stay that were selected for the first model are detailed in Table SM3-1. A total of 45 features were included in the model from the 161 candidate features.

Calibration plots of the model for both development and validation sets are shown in Figure SM3-1. Figure SM3-2 shows the AUROC values achieved by the first model (RS_1) for predicting the adverse event (combined outcome) within an increasingly high number of days after discharge from ICU. The curves of the AUROC values for each outcome, in-hospital death or ICU re-admission, separately, are also shown.

Table SM3-1. Features computed from information acquired during the patients' ICU stay used in the first model, and the corresponding odds ratio and 95% confidence interval (95% CI) for the odds ratio. The normalisation parameters for each feature (mean and standard deviation, SD) are also shown.

Figure SM3-1. Calibration curves for the first model (RS_1) for predicting a future adverse event after ICU discharge. The left-hand panel shows the calibration curve on the development dataset, the right-hand panel shows the calibration curve of the developed model on the validation dataset. The grey line depicts an ideal calibration, representing a perfect correspondence between the observed events and the predicted events for each decile of score.

Figure SM3-2. Performance of the first model (as given by the AUROC) for prediction of cumulative in-hospital deaths, cumulative ICU re-admissions, and cumulative adverse events up to a cut-off day after ICU discharge. The left-hand panel shows the results using the development dataset, and the right-hand panel shows the performance using the validation dataset. The bottom graphs show the proportion of each event (N) over time for the three outcomes considered.

Vital-sign based model (RS_2)

For the second scoring system, we considered the construction of a multivariate, (possibly) multimodal model of normality, based on the vital-sign observation sets recorded during the 24 hours that preceded discharge home or during the last post-ICU day with recorded data (which corresponds to the 14th day) if the patient had not

For peer review only

been discharged, died or re-admitted to the ICU. These observation sets contain the vital signs from the most stable period of the patient's stay, because these data were acquired when patients are at their most "normal" state after discharge from ICU. This set of "normal" data contains $N = 1{,}082$ 5-dimensional vital-sign vectors. Table SM3-2 gives the corresponding means and standard deviations of this subset of data, used to train the model.

Table SM3-2. Vital signs' mean and standard deviation (SD) values for observations recorded in during the most "stable" period considered.

For peer review only

Figure SM3-1.

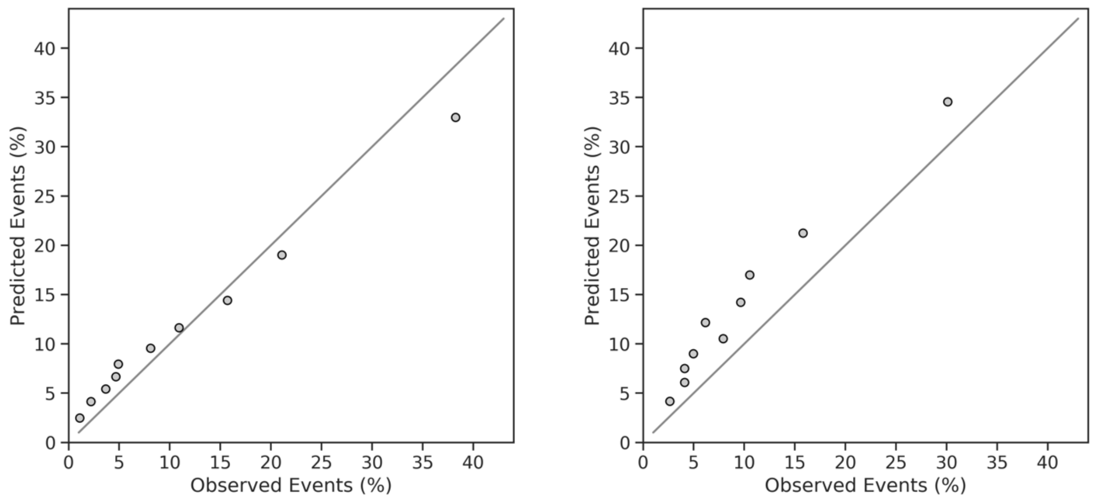

peer review only

Figure SM3-2.

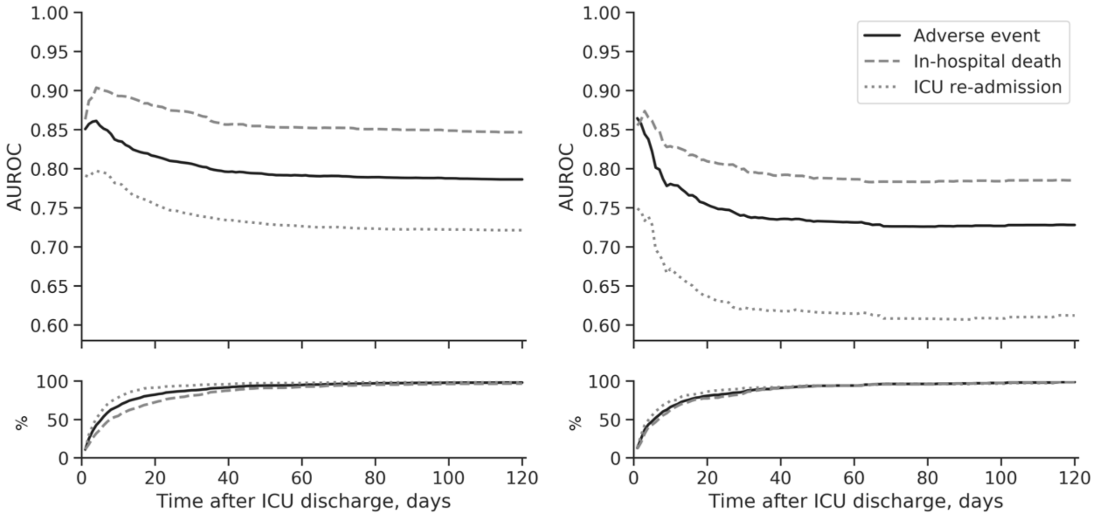

For peer review only

Table SM3-1.

Feature description	Mean (SD)		Odds ratio	95% CI
**Demographic and administrative information (**3 features**)**				
Age at admission to ICU	59	(17)	1.305	1.196 to 1.425
Time spend in the ICU, in days	4.3	(19.5)	1.134	1.072 to 1.199
Time from acute hospital admission and ICU admission, in days	4.2	(7.4)	1.038	0.964 to 1.119
**Vital signs related (**13 features**)**				
Last ventricular heart rate	86	(17)	1.135	1.024 to 1.259
Highest respiratory rate in the last 72 hours	26	(7)	1.129	1.033 to 1.235
Lowest ventricular heart rate in the last 72 hours	70	(16)	1.056	0.927 to 1.202
Highest ventricular heart rate in the first 24 hours	105	(22)	1.039	0.944 to 1.143
Last respiratory rate	18	(5)	1.026	0.943 to 1.116
Lowest ventricular heart rate in the first 24 hours	72	(17)	1.023	0.901 to 1.161
Highest SpO_2 recorded using pulse oximetry in the last 72 hours	100	(1)	1.016	0.944 to 1.094
Lowest respiratory rate in the first 24 hours	12	(3)	1.002	0.925 to 1.085
Lowest temperature in the first 24 hours	37.0	(0.7)	0.984	0.906 to 1.068
Highest temperature in the first 24 hours	35.5	(0.8)	0.981	0.889 to 1.083
Last mean arterial blood pressure	91	(16)	0.921	0.853 to 0.995
Lowest temperature in the last 72 hours	35.5	(0.7)	0.917	0.831 to 1.011
Last total GCS recorded	14	(1)	0.854	0.805 to 0.906
**Laboratory test results related (**24 features**)**				
Last plasma urea	9.1	(7.1)	1.313	1.186 to 1.453
Last plasma sodium	139	(4)	1.152	1.073 to 1.237
Highest INR in the first 24 hours	1.23	(0.48)	1.063	0.982 to 1.151
Last plasma bilirubin	12.8	(14.8)	1.052	0.984 to 1.124
Highest pH in the first 24 hours	7.43	(0.06)	1.051	0.971 to 1.138
Highest C-reactive protein in the first 24 hours	91	(55)	1.050	0.939 to 1.173
Last plasma lactate	1.16	(0.71)	1.028	0.957 to 1.103
Last INR	1.13	(0.35)	1.027	0.951 to 1.111
Last C-reactive protein	89	(54)	1.009	0.904 to 1.126
Highest platelet count in the first 24 hours	200	(109)	1.006	0.929 to 1.089
Last plasma sodium was in the extremes of the development set range (> or <)	-	-	1.003	0.831 to 1.211
Last plasma creatinine	116	(130)	0.993	0.735 to 1.342
The variability of $AaDO_2$ in the last 72 hours	6.1	(5.1)	0.990	0.908 to 1.080
Highest weight in the first 24 hours	77	(20)	0.989	0.909 to 1.076
Last haematocrit	32	(5)	0.989	0.881 to 1.110
Highest creatinine in the last 24 hours	124	(142)	0.975	0.744 to 1.278

For peer review only

Lowest haemoglobin in the first 24 hours	9.6	(2.0)	0.963	0.864 to 1.075
Highest white blood cell count in the first 24 hours	13.9	(7.0)	0.962	0.893 to 1.036
Lowest plasma potassium in the first 24 hours	3.8	(0.5)	0.951	0.879 to 1.030
Highest plasma creatinine in the last 72 hours	134	(153)	0.950	0.752 to 1.200
Total urine output in the first 24 hours	1772	(1260)	0.937	0.842 to 1.043
Last PaO_2/FiO_2 ratio	43.7	(15.8)	0.904	0.826 to 0.990
Last plasma albumin	28	(6)	0.870	0.798 to 0.949
Total urine output in the last 24 hours	2007	(1293)	0.781	0.703 to 0.867
**Procedures and treatments related (**5 features**)**				
If enteral feeding was used at any point during ICU stay	-	-	1.119	0.946 to 1.323
If a central venous catheter was present at ICU discharge	-	-	1.118	0.957 to 1.306
If an insulin infusion was used in the last 24 hours	-	-	1.055	0.894 to 1.246
If parenteral feeding was used at any point during ICU stay	-	-	1.044	0.799 to 1.363
A dysrhythmia at any point during ICU stay	-	-	1.001	0.999 to 1.003

For peer review only

Table SM3-2.

Vital sign	Mean (SD)
Heart rate, beats per min.	83 (14)
Respiratory rate, breaths per min.	17 (2)
Systolic blood pressure, mmHg	129 (19)
SpO_2, %	97 (3)
Temperature, C	36.3 (0.5)

For peer review only

Reporting checklist for prediction model development/validation.

Based on the TRIPOD guidelines.

Instructions to authors

Complete this checklist by entering the page numbers from your manuscript where readers will find each of the items listed below.

Your article may not currently address all the items on the checklist. Please modify your text to include the missing information. If you are certain that an item does not apply, please write "n/a" and provide a short explanation.

Upload your completed checklist as an extra file when you submit to a journal.

In your methods section, say that you used the TRIPODreporting guidelines, and cite them as:

Collins GS, Reitsma JB, Altman DG, Moons KG. Transparent reporting of a multivariable prediction model for individual prognosis or diagnosis (TRIPOD): The TRIPOD statement.

		Reporting Item	Page Number
**Title**			
	#1	Identify the study as developing and / or validating a multivariable prediction model, the target population, and the outcome to be predicted.	1
**Abstract**			

For peer review only - http://bmjopen.bmj.com/site/about/guidelines.xhtml

	#2	Provide a summary of objectives, study design, setting, participants, sample size, predictors, outcome, statistical analysis, results, and conclusions.	3
**Introduction**			
	#3a	Explain the medical context (including whether diagnostic or prognostic) and rationale for developing or validating the multivariable prediction model, including references to existing models.	4
	#3b	Specify the objectives, including whether the study describes the development or validation of the model or both.	4-8
**Methods**			
Source of data	#4a	Describe the study design or source of data (e.g., randomized trial, cohort, or registry data), separately for the development and validation data sets, if applicable.	5
Source of data	#4b	Specify the key study dates, including start of accrual; end of accrual; and, if applicable, end of follow-up.	5
Participants	#5a	Specify key elements of the study setting (e.g., primary care, secondary care, general population) including number and location of centres.	5
Participants	#5b	Describe eligibility criteria for participants.	5
Participants	#5c	Give details of treatments received, if relevant	N/A

For peer review only

Outcome	#6a	Clearly define the outcome that is predicted by the prediction model, including how and when assessed.	6
Outcome	#6b	Report any actions to blind assessment of the outcome to be predicted.	N/A
Predictors	#7a	Clearly define all predictors used in developing or validating the multivariable prediction model, including how and when they were measured	6
Predictors	#7b	Report any actions to blind assessment of predictors for the outcome and other predictors.	N/A
Sample size	#8	Explain how the study size was arrived at.	N/A
Missing data	#9	Describe how missing data were handled (e.g., complete-case analysis, single imputation, multiple imputation) with details of any imputation method.	5
Statistical analysis methods	#10a	If you are developing a prediction model describe how predictors were handled in the analyses.	7-8
Statistical analysis methods	#10b	If you are developing a prediction model, specify type of model, all model-building procedures (including any predictor selection), and method for internal validation.	7-8
Statistical analysis methods	#10c	If you are validating a prediction model, describe how the predictions were calculated.	7-8
Statistical analysis methods	#10d	Specify all measures used to assess model performance and, if relevant, to compare multiple models.	7-8

For peer review only

Statistical analysis methods	#10e	If you are validating a prediction model, describe any model updating (e.g., recalibration) arising from the validation, if done	7-8
Risk groups	#11	Provide details on how risk groups were created, if done.	N/A
Development vs. validation	#12	For validation, identify any differences from the development data in setting, eligibility criteria, outcome, and predictors.	5

Results

Participants	#13a	Describe the flow of participants through the study, including the number of participants with and without the outcome and, if applicable, a summary of the follow-up time. A diagram may be helpful.	5
Participants	#13b	Describe the characteristics of the participants (basic demographics, clinical features, available predictors), including the number of participants with missing data for predictors and outcome.	Table 1
Participants	#13c	For validation, show a comparison with the development data of the distribution of important variables (demographics, predictors and outcome).	Table 1
Model development	#14a	If developing a model, specify the number of participants and outcome events in each analysis.	8
Model development	#14b	If developing a model, report the unadjusted association, if calculated between each candidate predictor and outcome.	9

For peer review only

Model specification	#15a	If developing a model, present the full prediction model to allow predictions for individuals (i.e., all regression coefficients, and model intercept or baseline survival at a given time point).	9
Model specification	#15b	If developing a prediction model, explain how to the use it.	N/A
Model performance	#16	Report performance measures (with CIs) for the prediction model.	9
Model-updating	#17	If validating a model, report the results from any model updating, if done (i.e., model specification, model performance).	N/A
**Discussion**			
Limitations	#18	Discuss any limitations of the study (such as nonrepresentative sample, few events per predictor, missing data).	11
Interpretation	#19a	For validation, discuss the results with reference to performance in the development data, and any other validation data	9-11
Interpretation	#19b	Give an overall interpretation of the results, considering objectives, limitations, results from similar studies, and other relevant evidence.	9-11
Implications	#20	Discuss the potential clinical use of the model and implications for future research	12

For peer review only

Other information

Supplementary information	#21	Provide information about the availability of supplementary resources, such as study protocol, Web calculator, and data sets.	19
Funding	#22	Give the source of funding and the role of the funders for the present study.	13

The TRIPOD checklist is distributed under the terms of the Creative Commons Attribution License CC-BY. This checklist was completed on 11. April 2023 using https://www.goodreports.org/, a tool made by the EQUATOR Network in collaboration with Penelope.ai

For peer review only
